# Environmental determinants of islet autoimmunity (ENDIA): a pregnancy to early life cohort study in children at-risk of type 1 diabetes

**DOI:** 10.1186/1471-2431-13-124

**Published:** 2013-08-14

**Authors:** Megan AS Penno, Jennifer J Couper, Maria E Craig, Peter G Colman, William D Rawlinson, Andrew M Cotterill, Timothy W Jones, Leonard C Harrison

**Affiliations:** 1 Discipline of Paediatrics, School of Paediatrics and Reproductive Health, University of Adelaide, Adelaide, SA 5005, Australia; 2 Endocrine and Diabetes Department, Women’s and Children’s Hospital, North Adelaide, SA 5006, Australia; 3 Virology Division, Department of Microbiology, South Eastern Area Laboratory Services, Prince of Wales Hospital, Randwick, NSW 2031, Australia; 4 School of Women’s and Children’s Health, University of New South Wales, Sydney, NSW 2052, Australia; 5 Institute of Endocrinology and Diabetes, The Children’s Hospital at Westmead, Westmead, NSW 2145, Australia; 6 Discipline of Paediatrics and Child Health, University of Sydney, Sydney, NSW 2006, Australia; 7 Royal Melbourne Hospital, Parkville, Victoria 3050, Australia; 8 SOMS Faculty of Medicine, and BABS Faculty of Science, University of New South Wales, Sydney, NSW 2052, Australia; 9 Mater Health Services, South Brisbane, Queensland 4101, Australia; 10 Princess Margaret Hospital Department of Endocrinology and Diabetes, School of Paediatrics and Child Health; Telethon Institute of Child Health Research, University of Western Australia, Perth, WA 6008, Australia; 11 Walter and Eliza Hall Institute, Parkville, Victoria 3050, Australia

**Keywords:** Type 1 diabetes, Islet autoimmunity, Beta cell, Pregnancy, Infancy, Microbiome, Insulin resistance, Immunity, Virus, Systems biology

## Abstract

**Background:**

The incidence of type 1 diabetes has increased worldwide, particularly in younger children and those with lower genetic susceptibility. These observations suggest factors in the modern environment promote pancreatic islet autoimmunity and destruction of insulin-producing beta cells. The Environmental Determinants of Islet Autoimmunity (ENDIA) Study is investigating candidate environmental exposures and gene-environment interactions that may contribute to the development of islet autoimmunity and type 1 diabetes.

**Methods/design:**

ENDIA is the only prospective pregnancy/birth cohort study in the Southern Hemisphere investigating the determinants of type 1 diabetes in at-risk children. The study will recruit 1,400 unborn infants or infants less than six months of age with a first-degree relative (i.e. mother, father or sibling) with type 1 diabetes, across five Australian states. Pregnant mothers/infants will be followed prospectively from early pregnancy through childhood to investigate relationships between genotype, the development of islet autoimmunity (and subsequently type 1 diabetes), and prenatal and postnatal environmental factors. ENDIA will evaluate the microbiome, nutrition, bodyweight/composition, metabolome-lipidome, insulin resistance, innate and adaptive immune function and viral infections. A systems biology approach will be used to integrate these data. Investigation will be by 3-monthly assessments of the mother during pregnancy, then 3-monthly assessments of the child until 24 months of age and 6-monthly thereafter. The primary outcome measure is persistent islet autoimmunity, defined as the presence of autoantibodies to one or more islet autoantigens on consecutive tests.

**Discussion:**

Defining gene-environment interactions that initiate and/or promote destruction of the insulin-producing beta cells in early life will inform approaches to primary prevention of type 1 diabetes. The strength of ENDIA is the prospective, comprehensive and frequent systems-wide profiling from early pregnancy through to early childhood, to capture dynamic environmental exposures that may shape the development of islet autoimmunity.

**Trial registration:**

Australia New Zealand Clinical Trials Registry ACTRN12613000794707.

## Background

### The rising incidence of T1D and gene-environment interaction

Type 1 diabetes (T1D), one of the most common childhood-onset chronic diseases, is associated with enormous human and economic costs. The incidence of T1D is increasing worldwide [1] with a younger age of onset described in European and Australian populations [2,3]. In the 1980s the mean adjusted incidence rate of T1D in Australia was ~11 per 100,000 person-years [4,5]. By 2006, this had increased to 21.7 per 100,000 person-years, with a 4.1% increase in the diagnosis of children less than 15 years of age from 1999 to 2006 [3].

The doubling in incidence of T1D in Australia over the past two decades is consistent with a major role for the modern environment in T1D pathogenesis. International evidence to support this includes: (a) the reduced relative frequency of high risk genotypes in newly diagnosed children; (b) a less than 40% concordance of T1D in monozygotic twins; (c) discrepancies in disease incidence among genetically similar populations living in different regions, and (d) migration studies that show T1D incidence increases as populations move from low-risk to high-risk areas [6].

The HLA region on chromosome 6p contributes approximately half of the genetic susceptibility to T1D, yet the relative frequency of high risk HLA class II genotypes in children presenting with T1D has decreased as the incidence of the disease has increased [7]. Moreover, the recognition that over 60 gene loci are associated with T1D has led to speculation that T1D is a disease with discrete genetic subtypes whereby susceptibility genes interact differently with environmental exposures [8]. Variation in gene-environment interactions may explain different effects of environmental exposures in different populations at-risk of T1D. For example, caesarean section increases the risk of progression from islet autoimmunity to T1D in individuals with T1D susceptible *IFIH1* genotypes [9], whereas the early introduction of cow’s milk may increase risk of islet autoimmunity only in some at-risk genotypes [10]. As the immunological processes associated with the development of islet autoantibodies and T1D occur months to years prior to the onset of clinical disease, a long asymptomatic period provides the opportunity for prediction and, potentially, prevention [11].

Prospective studies, including our own, reveal the development of islet autoimmunity may occur at any point during the first years of life [12] through to adolescence [13], although recent data pooled from three large international cohorts have indicated the median age at seroconversion is 2.1 years [14]. Once persistent islet autoimmunity has developed the risk of progressing to T1D by 15 years of age is 12.7% in children with a single islet autoantibody, 61.6% in children with two islet autoantibodies and 79.1% in children with three islet autoantibodies compared with a 0.4% risk in children with no islet autoantibodies [14]. Moreover, progression to T1D is faster in children who: (a) seroconvert prior to three years of age, (b) have human leukocyte antigen (HLA) genotype DR3/DR4-DQ8, and (c) are female [14].

The aim of the Environmental Determinants of Islet Autoimmunity (ENDIA) study is to identify environmental factors, and gene-environment interactions, that contribute to and protect against the development of islet autoimmunity and progression to T1D in children genetically at-risk of T1D. We will follow 1,400 infants with a first-degree relative (FDR) with T1D prospectively from early pregnancy into childhood to investigate relationships between prenatal and postnatal environmental factors, and the development of islet autoimmunity and subsequent T1D. ENDIA will evaluate the microbiome, nutrition, bodyweight/composition, metabolome-lipidome, insulin resistance, innate and adaptive immune function and viral infections. A systems biology approach will integrate multi-omics analyses to explore hypotheses and mechanisms underlying the development of islet autoimmunity.

### The microbiome and islet autoimmunity

#### Contributors to aberrant development of the microbiome

Humans live in a symbiotic relationship with trillions of microorganisms collectively known as the commensal microbiome [15]. The establishment of the microbiome begins at birth when the neonate is exposed to microorganisms derived from the mother and immediate environment [16]. The composition of the microbiome changes rapidly during early life until it is similar to that of an adult by two years of age [17].

Several environmental factors contribute to shaping the composition of microbiome including genotype, mode of delivery, antibiotic use and microbial exposure in early life, time of first fever, nutrition and weight gain in early life [18,19]. Equally, contemporary changes in these factors have also been linked to T1D. For example, the proportion of Australian deliveries by caesarean section has increased from 21% in 1998 to 31% in 2007 [20]. Vaginally delivered infants acquire bacterial communities resembling their own mother’s vaginal microbiome whereas infants delivered by caesarean section harbour communities similar to those found on the skin [16]. This change in the initial microbiome may lead to alternative microbial succession patterns that persist over time and contribute to variations in normal physiology and/or to disease risk [16]. A meta-analysis of international observational studies showed a 20% increase in the incidence of T1D in children delivered by caesarean section [17]. As a second example, the prevalence of overweight and obesity, currently at 34-50% [21] in pregnancy and 27% [22] in childhood in Australia, has increased over the last 20 years. With excessive weight gain in pregnancy, Bifidobacterium counts are lower in the mother’s breast milk, which in turn impacts upon the microbiome of the infant [17]. Early childhood weight gain is associated with an increased risk of islet autoimmunity [12] while childhood obesity is preceded by lower counts of Bifidobacterium at six and 12 months of age [17]. Finally, antibiotic use, which has a direct effect on the gut microbiome [18], has increased in children aged over two in the last 10 years in Australia, although data during the first two years of life are lacking [20].

#### The gut microbiome and host immune system

The development of the mucosal immune system, and maturation of the systemic immune system, depends on bacterial colonisation of the mucosa [23]. A normal mucosal immune system is necessary to generate regulatory T cells (Treg) in response to oral antigens [24]. The essential role of the mucosal immune system in maintaining immune homeostasis is illustrated by the effect of a germ-free versus a conventional ‘dirty’ environment on the incidence of spontaneous autoimmune diabetes in the non-obese diabetic (NOD) mouse, the best animal model of T1D. The incidence of spontaneous diabetes in NOD mice differs greatly between colonies around the world and is inversely correlated with exposure to microbial infection [25]. The high incidence of diabetes in NOD mice housed under pathogen-free conditions is reduced by conventional conditions of housing and feeding [26,27]. Under ‘dirty’ conditions, bacterial colonisation of the intestine is accompanied by maturation of mucosal immune function [28]. Emerging evidence indicates that the gut microbiome differs in composition and function between children at risk for T1D and case controls [29]. Supplementary to the microbiome, breast milk contains growth factors, cytokines and other immunomodulatory agents that promote functional maturation of the intestinal mucosa and mucosal immune system. Breast milk also contains endogenous insulin [30], probably the key autoantigen in T1D [31], and ‘oral tolerance’ to insulin conceivably might protect against the development of T1D.

#### Changes in the gut microbiome associated with islet autoimmunity and T1D

Experimental diabetes models show that specific bacteria and their products program Treg and T helper-17 (Th17) cell development [32]. However, it is yet to be confirmed if and how human gut microbes contribute to the development of islet autoimmunity. Two small European studies involving a total of 17 children with islet autoimmunity and 17 age/gender/HLA genotype matched controls reported decreased microbial diversity in stools from children with islet autoimmunity [33,34], while metagenomic analysis revealed that children with islet autoimmunity have less butyrate-producing and mucin-degrading bacteria [35]. This has led to speculation that the normal process during the first two years of life by which a healthy microbiome becomes more diverse and stable, is not seen in children who develop islet autoimmunity and T1D. Socio-economic and cultural factors may play a role in shaping the diversity of the microbiome. For example, the incidence of T1D in Finland is 41.4 per 100,000 people per year, yet in neighbouring Russian Karelia it is only 7.4 per 100,000 people per year, despite these populations sharing a genetically similar background [36]. Karelian children show significantly higher infection rates than their Finnish peers, which alters the composition of the gut microbiome [37]. The challenge is to determine how these alterations relate to the pathogenesis of T1D.

#### Therapeutic implications of the microbiome for T1D

If the microbiome is shaped by the early environment and if specific changes in the microbiome are associated with susceptibility to T1D, then in theory it should be possible to re-engineer the microbiome and reduce the incidence of T1D. This might be achieved in several ways, for example by reducing the frequency of caesarean births, avoiding unnecessary administration of antibiotics, promoting breast feeding, modifying the early infant diet, avoiding excessive weight gain and administering pre- and pro-biotics. Administration of probiotics to NOD mice has been shown to reduce their incidence of spontaneous autoimmune diabetes [38].

### Effects of weight gain on the development of islet autoimmunity and T1D

#### Pregnancy weight gain and perinatal risk factors influencing islet autoimmunity

In a retrospective study, higher maternal body mass index (BMI) before pregnancy and weight gain >15 kg during pregnancy predicted risk of islet autoimmunity in offspring with the high-risk HLA genotype, DR4-DQ8/DR3-DQ2 [39]. No prospective data during pregnancy have been published, however, perinatal risk factors have been analysed in the German BabyDiab Study. Birth weight in the middle tertile (3,250-3,700 g) was associated with increased islet autoimmunity risk, as was maternal HbA1c greater than 7% [40]. The presence of islet autoantibodies in cord blood may also be a marker of reduced risk for T1D in offspring [41,42].

#### Weight gain and nutrition during childhood

The rise in childhood incidence of T1D parallels the overweight/obesity epidemic in Western childhood populations. Some evidence exists for an effect of early childhood weight gain on risk of islet autoimmunity. In the first Australian birth cohort of at-risk children (Australian BabyDiab study) weight z-score and body mass index (BMI) z-score were continuous predictors of risk of islet autoimmunity [12]. Similarly, in the at-risk Diabetes Autoimmunity Study in the Young (DAISY) birth cohort followed in Colorado, height velocity from age two was associated with risk of islet autoimmunity [43]. However, children in the German BabyDiab cohort showed no effect of weight gain on risk of islet autoimmunity [44]. Younger age of T1D onset is inconsistently associated with higher BMI at diagnosis [45,46]. Retrospective case control studies from Scandinavia link increased linear growth and weight gain in early childhood with T1D [47].

Prospective birth cohorts show mixed data on the duration of exclusive breastfeeding and effect of solid food exposure on risk of islet autoimmunity [48-54]. An early analysis from the Trial to Reduce IDDM in the Genetically at-Risk (TRIGR) study suggests that feeding a casein hydrolysate formula to Finnish infants at-risk of T1D following cessation of breastfeeding reduced risk of islet autoimmunity [55]. Recent evidence from the DIASY study shows that both early and late first exposure to any solid food predicts development of T1D, leading to speculation that early exposure to solid foods evokes an abnormal response in the immature gut; while late exposures may relate to the cessation of breastfeeding before solid foods introduction so that the protective effects of breast milk are lost before the introduction of foreign food antigens [56].

#### Links between weight gain, systemic inflammation and insulin resistance

Weight gain is associated with markers of innate immune system activation and chronic low-grade inflammation, which have been linked to insulin resistance. Previous studies have shown that pro-inflammatory CD11c + adipose tissue macrophages impair insulin-stimulated glucose uptake by human adipocytes, providing a possible mechanistic link between obesity and insulin resistance [57]. In addition, adipose tissue macrophages metabolize lipid and may initiate adaptive immune responses by adipose tissue T cells, which are activated in adipose tissue in obesity. Infants born small for gestational age with catch up growth have accelerated weight gain and insulin resistance in early life. Appropriate for gestational age but premature babies also have reduced insulin sensitivity [58]. The mechanism is unknown but underscores the importance of prospective studies from pregnancy in at-risk children.

### Innate and adaptive immune function and risk of islet autoimmunity and T1D

#### Pro-inflammatory mediators and anti-inflammatory dietary components in T1D

Circulating pro-inflammatory cytokines are increased at diagnosis in T1D [59-61] and, in a nested case–control study of 67 children enrolled in the Viruses in the Genetically at Risk (VIGR) birth cohort study (27 with islet antoantibodies; 40 age-matched antibody negative controls), pro-inflammatory cytokines and chemokines were increased in children with islet autoimmunity [62]. Whilst longitudinal studies before diagnosis are lacking, the DAISY Study found that serum C-reactive protein concentrations predicted progression to islet autoimmunity in early childhood [63]. Chronic low-grade inflammation in the mother has been linked to insulin resistance in the offspring. Thus, concentrations of serum pro-inflammatory advanced glycation end products (AGEs) at the end of healthy pregnancies were associated with lower adiponectin and higher insulin levels in the offspring [64]. We will measure both AGEs in pregnancy and AGEs and adiponectin in the offspring in ENDIA.

The omega-3 fatty acids docosahexaenoic acid (DHA) and eicosapentaenoic acid (EPA) reduce inflammatory cytokines and inflammatory prostaglandins, both of which contribute to the initial islet lesion. Accordingly, higher intake of DHA and EPA might lower the risk of T1D, but definite evidence is lacking. Cod liver oil, omega-3 fatty acids and vitamin D taken during pregnancy or infancy have been associated with a lower incidence of T1D in a case control study [65] and increased dietary omega-3 fatty acid intake in infancy is weakly associated with a lower risk of islet autoimmunity [66]. Therefore, protective effects of these natural anti-inflammatory dietary components may occur during pregnancy, during infancy, or both. DHA and EPA inhibit prostaglandin E2 (PGE_2_) synthase with equivalent or greater potency than other fatty acid analogues.

Vitamin D is an anti-inflammatory steroid with multiple immunomodulatory effects that promotes immune tolerance. The vitamin D receptor (VDR) is expressed by all cells of the immune system, while activated macrophages and dendritic cells express 1α hydroxylase, the enzyme that converts 25-hydroxyvitamin D3 (25 OHD) to its metabolically active form, 1,25 OHD. Polymorphisms in both the *VDR* and the gene that encodes 1α hydroxylase, *CYP27B1* are associated with T1D risk [67-69]. Two Northern European cross-sectional studies have shown an inverse relationship between vitamin D supplementation in infancy and T1D incidence [65,70], but the DAISY Study found no relationship between 25 OHD levels and risk of islet autoimmunity or progression to T1D [71]. Population 25 OHD levels have fallen over the last 50 years with urbanisation, avoidance of or less access to sunlight, decreased intake of oily fish and a lower recommended daily intake of vitamin D. In Australia, vitamin D insufficiency is common in pregnancy [72].

#### Adaptive immune function

Aberrant immune responses to self-antigens are controlled by tolerance mechanisms, including suppression by Treg cells. The impaired generation or function of Treg cells, or resistance to their action, could contribute to the development of autoimmune disease [73]. Indeed, an imbalance between Treg and effector T cells has been linked to T1D [74]. Natural CD4+ Treg are generated in the thymus during development and induced CD4+ Treg (iTreg) by ‘tolerogenic’ conditions of antigen presentation postnatally in the periphery, especially in the mucosa. The microbiome is essential for development of the mucosal immune system, which in turn is required for the generation of iTreg. Treg are defined by high expression of CD25 and stable expression of the transcription factor FOXP3, which is critical to maintain expression of genes required for suppressor function and prevent expression of genes required for effector function [75]. FOXP3 is also expressed transiently by activated T cells but its stable expression in Treg is associated with demethylation at the *FOXP3* locus. We have identified another marker of stable Treg, namely peptidase inhibitor 16 (CD359), which will be used to enumerate Treg in the ENDIA Study [75].

In addition to conventional Treg, we have identified a sub-population of CD4^+^ T cells with antigen-activated suppressor function that are required to prevent autoimmune diabetes in the NOD mouse model of T1D [76]. These cells are characterised by high expression of CD52 and exert suppression by releasing soluble CD52, which ligates inhibitory Siglec receptors. We observed that the frequency and function of CD52^hi^ CD4^+^ T cells in response to the islet autoantigen, glutamic acid decarboxylase 65 (GAD65), was impaired in children at risk for T1D [76]. The characterisation of islet autoantigen-specific T cells, including CD52^hi^ CD4^+^ T cells, will be a critical component of the ENDIA Study.

Enhanced innate immunity promoted by the environment may drive adaptive immunity by impairing regulatory and stimulating pathogenic T cell immunity. In the ENDIA Study, we will measure cytokines and chemokines produced in whole blood *in vitro* in response to innate immune (TLR agonists) stimuli and adaptive immune (islet [proinsulin, GAD65] and control [childhood vaccines]) antigens [77].

### Viral infection is associated with islet autoimmunity and T1D

Viruses might trigger the onset of islet autoimmunity and/or promote the progression of established islet autoimmunity. Prospective studies from birth are required to obtain evidence in support of these possibilities; direct proof of a causal role for viruses will be much harder to obtain. In a meta-analysis, we recently reported evidence for a role for enterovirus (EV) at the clinical onset of T1D [78], with an odds ratio (OR) ~10 for EV infection [79]. We have also found a significant association between EV infection and islet autoimmunity [78], with an OR of ~4 in children with EV infection. Data on the role of EV infection as an initiator [80,81] or accelerator [82] of islet autoimmunity in longitudinal studies of genetically-predisposed children are conflicting, although some of these differences may be related to the use of different techniques for determining EV infection. The temporal relationship between EV infection and development of islet autoimmunity needs to established, as well as the interaction between EV infection and other environmental determinants. For example, it has been reported that the effect of EV infection on the development of islet autoimmunity may be increased by early exposure to cow’s milk [83]. In particular, the effect of maternal exposure to the virus prenatally and of breastfeeding duration has not been examined. We found that EV infection is more frequent in recent-onset T1D in patients with co-existing vitamin D deficiency (unpublished data).

Rotavirus (RV) infection, the most common cause of infant diarrhoea, has been associated temporally with the first appearance of islet autoantibodies [84], and molecular mimicry has been demonstrated between human T cell epitopes in RV and pancreatic islet autoantigens [85]. The live RV vaccines RotaRix (GlaxoSmithKline) and RotaTeq (Merck/CSL) are now available to all Australian infants aged 6–32 weeks, with a 2008–2009 uptake rate of >80% [86] compared with <50% in US [87]. It will therefore be important to document if RV immunization alters the development of islet autoimmunity and T1D incidence. The lack of direct evidence for viral infection in causing T1D is partly methodological but also suggests multiple other and possibly ubiquitous environmental factors are at play.

### Omics studies in the development of islet autoimmunity and T1D

One of the challenges for interventional studies to prevent T1D is a lack of biomarkers that reliably correlate with disease activity. Consequently, study endpoints are limited to assessments of beta-cell function and/or diagnosis of T1D following months to years of intervention [74]. Changes in the dynamic metabolome, lipidome and/or proteome may provide suitable and sensitive biomarkers, whilst providing insight into the underlying mechanisms that initiate autoimmunity. For example, reduced levels of succinic acid at birth and reduced phospholipids and triglycerides in childhood, as measured within the blood metabolome, were reported to precede islet autoimmunity in children who progressed to T1D [88]. The same study also identified elevated serum glutamic acid prior to the appearance of GAD autoantibodies, leading to speculation that an increased glutamate load on the beta cells may increase activity of GAD65, a major beta-cell antigen, potentially triggering the autoimmune process. More recently, children who later developed islet autoimmunity and progressed to T1D were shown to have a distinct cord blood lipidomic profile with reduced major choline-containing phospholipids, implicating choline metabolism in pregnancy as a factor associated with progression to T1D, although not necessarily with development of islet autoimmunity per se [89].

Environmental factors are likely to modify the DNA template by chemical and other epigenetic changes. An epigenome-wide association study (EWAS) of DNA methylation profiles of purified CD14^+^ monocytes (an immune effector cell type relevant to T1D pathogenesis) from 15 pairs of monozygous twins discordant for T1D revealed 132 CpG sites that were differentially methylated in accordance with T1D status [90]. By genome-wide methylation analysis of human T cell subsets we identified previously unrecognised hypomethylated promoter regions regulated by FOXP3 in natural Treg [91]. Importantly, significantly less methylation was observed in many of these gene promoters in Treg from children with islet autoantibodies compared to healthy controls, pointing to widespread alterations in the epigenetic landscape. The ENDIA Study will allow the epigenetic landscape to be scanned across time.

### ENDIA in the context of international studies investigating the environment and T1D risk

To our knowledge there are no other studies in the Southern Hemisphere or South-East Asia investigating environmental determinants of T1D from pregnancy through early life. Internationally, the ENDIA Study will differ from The Environmental Determinants of Diabetes in the Young (TEDDY) [92], the TRIGR [93] and German BabyDiab [94] studies in that:

• ENDIA includes children on the basis of family history, irrespective of HLA genotype. This is relevant as the rising incidence of T1D is accounted for by individuals with lower risk HLA genotypes.

• Prospective samples will be collected for analysis of the microbiome from the gut (represented by stool), oral cavity, nares, skin and vagina during pregnancy and from breast milk during lactation (including colostrum) and 3-monthly in early childhood. Pregnancy and the first two years of life are critical periods for the development and establishment of the microbiota, underscoring the importance of prospective studies from pregnancy.

• Nutritional status and intake will be documented prospectively during pregnancy (from the first trimester) and lactation [95].

• Weight gain and physical activity (using validated tools [96]) will be measured prospectively during pregnancy.

• Nutritional status and intake in childhood will be documented by daily diary until weaning and then by multi-pass 24-h food recalls [97,98] until 24 months, and from two years by the Australian Toddler Eating Survey [99].

• Fasting blood samples will be collected in childhood to measure insulin resistance.

• The Australian population differs in many ways from those in the USA and Europe, e.g. in diet, UV exposure, and immunisation status (Australia has a much higher RV vaccine uptake than in USA).

### Hypothesis and objectives

#### Hypotheses

The unifying hypothesis is that gene-environment interactions during prenatal and postnatal development drive the development of islet autoimmunity and T1D in children at-risk for T1D.

The specific hypotheses are:

1. The maternal microbiome during pregnancy and lactation differs in composition, diversity and functional products between mothers whose offspring do and do not develop islet autoimmunity and T1D.

2. The microbiome differs in composition, diversity and functional products in children who do and do not develop islet autoimmunity and T1D during the first three years of life.

3. Accelerated weight gain during pregnancy, and accelerated weight gain and insulin resistance during the first three years of life, is associated with an increased risk of islet autoimmunity.

4. Viral infection during pregnancy and first three years of life modifies the risk of islet autoimmunity and T1D.

#### Objectives

1. To follow 1,400 children who have a first-degree relative (FDR) with T1D during pregnancy and early life to determine HLA genotype together with multiple susceptibility genes, changes in the microbiome, weight gain, metabolome-lipidome, insulin sensitivity, nutritional status, inflammatory markers, the timing and frequency of viral infections, and the relationships between genetic and environmental determinants.

2. To determine the relationship between changes in the microbiome and prenatal and postnatal exposures, including weight gain, metabolome-lipidome, insulin sensitivity, nutritional status, and viral infection, and the development of persistent islet autoimmunity in children with a FDR with T1D.

The long-term objective is to follow T1D at-risk children into adolescence to determine the relationship between genotype, the microbiome and the environment, and the development of islet autoimmunity and T1D.

## Methods/design

### Summary of design

This is a prospective cohort study of the offspring of 1,400 mothers who have T1D, or a FDR with T1D, from pregnancy through childhood. Recruitment and consent will occur during pregnancy. The first investigation occurs as soon as feasible after the mother has given consent and investigation is 3-monthly throughout the pregnancy. At birth there is investigation of the mother and child and the child is then followed 3-monthly for two years, and 6-monthly thereafter. The primary outcome measure is persistent islet autoimmunity. The secondary outcome measure is the development of T1D. An overview of the study activities across time is shown in Table 1.

**Table 1:** Study design overview

**Sample (parameter)**	**Description**	**Purpose**	**1**^**st **^**trimester †**	**2**^**nd **^**trimester**	**3**^**rd **^**trimester**	**1-2 days postnatal**	**3-5 days postnatal**	**3 m**	**6 m**	**9, 15, 21, 30 m**	**12, 18, 24, 36 m**	**Proband**
Informed consent	-	Informed consent	*									*
Demographics (including ancestry, socioeconomic status)	-	Maternal	*ψ									
		Paternal	*ψ									
		Infant				*ψ						
		Proband										*
Medical/obstetric history	-	Maternal	*	*	*							
		Paternal	*ψ									
		Proband										*
		OGTT results			*							
		Pregnancy complications					*ψ					
		Labour and delivery					*ψ					
		Congenital abnormalities					*ψ					
Anthropometry	-	Height, Weight, BMI	*	*	*		*					
		Weight z, length z, BMI z				*		*	*	*	*	
		Waist circumference				*		*	*	*	*	
		Lifestyle questionnaire	*	*	*			*	*	*	*	
Blood	A: Serum (red)	AutoAbs: Melbourne			*	*^Δ^		*	*	*	*	
		AutoAbs: Adelaide			*	*^Δ^		*	*	*	*	
		Resistance: adiponectin, hsCRP, C-peptide, insulin, glucose	*	*	*	*^Δ^		*	*	*	*	
		Metabolome/lipidome	*	*	*	*^Δ^		*	*	*	*	
	B: Serum (red)	Vitamin D (25 OHD) / tTGAb / C-peptide / glucose	25 OHD tTGAb	25 OHD tTGAb	25 OHD tTGAb	25 OHD tTGAb C-peptideΔ		25 OHD tTGAb	tTGAb	25 OHD tTGAb	tTGAb glucose (12, 24, 36 m)	
	C: LH (green)	Cytokines/chemokines (innate immunity)						*	*	*	*	
	D: K-EDTA (purple)	Buffy coat (epigenome)	*	*	*	*^Δ^		*	*	*	*	
		RBC	*	*	*	*^Δ^		*	*	*	*	
		Virology	*	*	*	*^ΔΩ^		*	*	*	*	
		DHA, EPA, resolvins, protectins, prostaglandin E2	*	*	*	*^Δ^		*		*		
		Advanced glycation end products	*	*	*	*^Δ^			*		*	
		Haemoglobin A1C (HbA1c)	*††	*††	*††	*^Δ^				*ϕ	*ϕ	
	E: K-EDTA (purple)	HLA typing				*^Δ^						*
	F: CPT sodium citrate (blue/black)	PBMCs (adaptive immunity)				*^Δ^			*	*	*	
Urine	Maternal: urine pot Infant: collection bag	Metabolome/lipidome	*	*	*		*	*	*	*	*	
		Advanced glycation end products	*	*	*		*		*		*	
Stool	Maternal: EasySampler Infant: stool collection kit	Microbiome	*	*	*		*	*	*	*	*	
		Virology	*	*	*		*	*	*	*	*	
		Metabolome/lipidome	*	*	*		*	*	*	*	*	
Saliva	Oragene kit	SNP typing						*				*
Tongue dorsum	ESwab swab	Microbiome	*	*	*		*	*	*	*	*	
Buccal mucosa	ESwab swab	Microbiome	*	*	*		*	*	*	*	*	
Throat	UTM	Virology	*	*	*		*	*	*	*	*	
Nasal	ESwab swab	Microbiome	*	*	*		*	*	*	*	*	
Skin (inner elbows)	ESwab swab	Microbiome			*	* m&i	* m&i	* m&i	* m&i	*	*	
Vagina	ESwab swab	Microbiome			*							
Colostrum (day 0–3)	Colostrum collection kit	Microbiome				*						
		Metabolome/lipidome				*						
Breast milk	Milk collection kit	Microbiome					*	*ζ	*ζ	*ζ	*ζ	
		Metabolome/lipidome					*	*ζ	*ζ	*V3 ζ		
Diet	Maternal diet evaluation tools	DQES version 2			*			*ζ				
	Infant diet evaluation tools	Infant diary (birth-6 m)						*	*			
		Infant diary (6-12 m)								* V3	* V4	
		24 hour recall (12 – 24 months)								* V5, V7	* V6	
		Australian Toddler Eating Survey (>24 months)								* V9	* V8, V10	

**†** if available.

ψ or at first opportunity.

**††** if mother has T1D.

ϕ if persistent islet autoantibodies are detected.

^Δ^ cord blood.

^Ω^ using Guthrie Card if cord blood sample not taken.

“m&i” maternal and infant.

ζ if breast feeding.

### Estimation of target population

The population in the five states (New South Wales (NSW), Western Australia (WA), Queensland (Qld), Victoria (Vic) and South Australia (SA)) participating in ENDIA is 20.8 million, i.e. ~92% of Australia’s total population, and of these 3,500,000 men and 3,500,000 women aged 15–40 years [100]. Conservatively, there are 90,000 people with T1D in Australia, of whom ~15,000 are aged 0–14 years and ~45,000 aged 15–40 years, affecting men and women equally. Therefore, the prevalence of T1D among Australian women aged 15–40 years is 22,500/3,500,000 or 0.6%. Every year 250,000 women give birth in Australia, of whom 0.6% (n = 1,500) have T1D. There will also be 1,500 new fathers each year with T1D. Thus, approximately 3,000 births annually are to a parent with T1D. In contrast, the prevalence of siblings with T1D is relatively low. The incidence of T1D in children aged 0–14 is 22 per 100,000 person years [3]. Of 250,000 newborns each year in Australia, approximately half (125,000) will have a sibling. If the majority of those have a sibling aged 0–14 years, only ~28 will have T1D. Therefore, there are approximately 3,000 potential participants per annum of whom we aim to recruit 400–475 per year.

### Determination of sample size

We estimate recruitment of 1,400 participants from the five states over three years, followed for a median of two years with a 9% rate of persistent islet autoimmunity. Maximum total dropout /lost to follow-up rate is estimated at 15% with the majority of dropouts occurring in the first six months of follow-up. These estimates are based on data from the Australian BabyDiab [12] and TRIGR [93] at-risk birth cohorts in which dropout rates were 15% and 7%, respectively.

Using nQuery Advisor, with 600 participants above the median and 600 below (1200 total after 15% drop out from 1,400), for a given exposure variable, power is 90% to detect a difference between survival (i.e. no islet autoimmunity) of 93% in one group and survival of 88% in the other – with 108 events (islet autoimmunity) or 9% of the total sample of 1,200 required [101]. For examining interactions between uncorrelated exposure variables with approximately 375 participants per combination of above/below the median, the power is 77% to detect a difference in survival (no islet autoimmunity) between 93% and 87% (70 events in 600 participants required).

### Recruitment, retention and withdrawal of participants

#### Inclusion criteria

The inclusion criteria for ENDIA are an unborn child with an FDR with T1D, targeting the mother for recruitment in first, second or third trimester of pregnancy, or an infant less than six months of age with a FDR with T1D.

#### Exclusion criteria

The only exclusion criterion is the incapacity for the pregnant woman (or in the case of postnatal recruitment, the child’s primary caregiver) to understand the requirements of her and/or her child’s participation. This may be due to illiteracy, an intellectual disability or a mental illness.

#### Withdrawal criteria

Participants will be withdrawn from the study if they develop T1D in the case of the child, or request to discontinue participation in the case of the mother on behalf of herself and the child. The reason for withdrawal from the study will be captured on the case report form. Every effort will be made to continue to follow all participants enrolled in the study except for those who withdraw consent. Participants who are withdrawn from the study will be contacted by telephone annually to determine their T1D status.

#### Recruitment

Participants will be identified during pregnancy or within six months of birth in NSW, WA, Qld, Vic and SA. FDRs will be mothers, fathers or siblings with T1D. Since majority will be a mother or a father with T1D, pregnant women with T1D or whose partner has T1D are the main focus of recruitment. Eligible participants will be identified from the following sources: (1) prospective mothers attending public and private antenatal clinics prior to delivery, (2) prospective parents attending public and private diabetes clinics, (3) pregnant mothers of existing patients attending paediatric diabetes clinics, (4) diabetes organisations, and (5) local and national mainstream advertising.

Private endocrinologists and obstetricians known to manage T1D in young adults will be targeted with visits from research staff and personalised letters, brochures and posters. Paediatric T1D clinics will recruit families in which a sibling has T1D. Our research team includes endocrinologists and a perinatologist, all with substantial experience in recruitment of participants during pregnancy.

State and national diabetes networks and advocacy groups will be used to promote the study, as well as a dedicated website (http://www.endia.org.au) and social media such as Facebook (https://www.facebook.com/endiastudy) and Twitter. All advertising material, media releases and other articles intended for the general public will be approved by the relevant Human Research Ethics Committees in each state prior to distribution.

#### Participant retention

Our past experience indicates that families affected by T1D are highly motivated to participate in research. The previous Australian BabyDiab birth cohort in SA and Victoria recruited 548 children followed over a median of 5.7 years, while the participant retention rate in the NSW arm of the TRIGR study was 87% after five years (80% planned) [93]. This high rate is attributed to frequent family phone contacts, newsletters, distribution of calendars with reminders and incentives such as birthday cards, the set-up of parent’s groups for T1D families and active assistance with surveillance of the health of the participating child. Similar strategies will be implemented in ENDIA.

Participant engagement in the study will be central to high retention rates. More than half of people within the target population (men and women aged 18–34) have a Facebook page [102]. The site will be monitored daily and updated regularly to maintain relevancy. We will also use Facebook to link with existing T1D networks and advocacy groups, including JDRF International, JDRF Australia and Diabetes Australia, thereby generating further awareness of the study.

### Study activities

#### Pregnancy visits 1 and 2: first and second trimesters

The following will be undertaken before 13 weeks gestation and again before 26 weeks gestation:

• Documentation of maternal and paternal demographics including medical and obstetric history

• Maternal anthropometry: height, weight, BMI

• Collection of blood sample for investigations defined in Table 1

• Collection of nasal, buccal, tongue and throat swabs, urine and stool specimens for microbiome studies

• Administration of the Pregnancy Lifestyle Questionnaire and Pregnancy Physical Activity Questionnaire [96]

• Documentation of proband history of T1D and other significant medical history

• Blood and saliva will also be collected from the proband on one occasion for HLA genotyping and SNP analysis, respectively

#### Pregnancy visit 3: third trimester

Between 24 and 38 weeks gestation investigations will be as for first/second trimester PLUS

• Documentation of results of the oral GTT, as routinely performed in pregnancy

• Explanation by research nurse (trained by study dietician) as to how to complete the DQES version 2 (DQESv2) questionnaire for nutrition analysis [95]

• Vaginal and skin swabs for analysis of the microbiome

• Documentation of pregnancy complications: hypertension, preeclampsia, HELLP, hyperemesis gravidarum

#### Birth and week 1 of life

The following will be undertaken across two study visits within the first week of life. The first, termed the B1 visit, will be within the first two days post-partum; the second, termed the B2 visit, will be 3–5 days post-partum.

• Collection of cord blood samples for investigations defined in Table 1

• Completion of the documentation of the pregnancy complications, and /or of any congenital abnormalities in the child

• Documentation of birth date, gestation, gender, birth weight, placental weight, birth length, APGAR score, method of delivery and any complications of the birth, perinatal and neonatal periods

• Record of maternal weight gain during pregnancy

• Viral studies, including serology and qPCR, will be performed from urine taken at B2 visit

• Collection of colostrum samples at B1 and B2 visits for microbiome studies

• Collection of skin swabs from infant and mother B1 and B2 visits for microbiome studies

• Collection of meconium (first nappy) at B1 visit and stool samples, nasal, buccal, tongue and throat swabs from infant at B2 visit, all for microbiome studies

#### Follow-up visits at 3, 6, 9, 12, 15, 18, 21, 24, 30 and 36 months of age

The following will be performed once:

• Saliva sampling from the infant for SNP genotype

• Documentation of maternal nutrition during lactation using the DQESv2 questionnaire at three months

The following will be undertaken at each visit:

• Collection of blood sample for investigations outlined in Table 1. If a child develops persistent islet autoimmunity, as defined above, HbA1c will be also measured 6-monthly

• Anthropometrics: length (height at 30 and 36 months), weight, BMI, waist circumference (at umbilicus)

• Collection of nasal, buccal, tongue, throat and skin swabs and stool samples for microbiome studies

• Nutrition and Lifestyle assessments (Refer to Additional file 1)

### Data collection, sampling procedures and laboratory investigations

A detailed description of the data collection, sampling procedures and laboratory investigations can be found in Additional file 1. Data collection tools developed specifically for the ENDIA Study evaluating infant feeing, maternal lifestyle in pregnancy, and maternal/infant lifestyle postpartum are provided in Additional files 2, 3 and 4, respectively.

### Analysis of the primary outcome measure

The primary outcome measure is islet autoimmunity defined as elevation of ≥ one islet autoantibody on consecutive tests. Time-to-event analyses will explore the effect of variables on the risk of islet autoimmunity. Unadjusted and adjusted Hazard Ratios (HR) will be calculated using parametric and non-parametric (proportional hazard) survival models. The models will account for right censored data due to variable length of follow up and loss of follow up of participants. Variables measured over time (continuous and categorical) will be entered into survival models as time-dependent covariates as these measurements will be repeatedly obtained from individual participants. Adjusted HRs will be used to analyse the development of islet autoimmunity while controlling for associated variables as we have described [12]. Viral infections will also be modelled using survival analysis techniques. A fixed (time-independent) covariate will be used to test for associations with antenatal/postnatal viral infections/events, and time-dependent variables will be constructed to indicate exposure to viral infections throughout the follow-up period for each child.

Logistic regression analysis will be used to compare the relationship between presence or absence of islet autoimmunity and other variables. For analysis of nested case controls and longitudinal changes in the microbiome, islet autoimmune and non-islet autoimmune participants will be matched for age, gender, HLA type, ethnicity, proband relationship, parity, mode of delivery and gestation/birth weight.

A systems biology approach will be used to reveal inter-relationships between prenatal and postnatal environmental exposures that may condition the microbiome, metabolome and epigenome – these include maternal T1D, nutrition, weight gain, physical activity and viral infection during pregnancy; and mode of delivery, accelerated weight gain, nutrition, immune function, early fever, antibiotic use and viral infection during early life.

### Safety parameters

#### Ethics committee review

In accordance with the Australian National Statement [103], in line with the ICH Guideline for Good Clinical Practice, the investigators have obtained written approval of the study protocol and Participant Information and Consent Form from the Human Research Ethics Committee (HREC) prior to commencement of the study. The study has been registered with the Australian New Zealand Clinical Trials Registry (ANZCTR) with registration number ACTRN12613000794707.

#### Subject information and consent

As this study involves mothers and children aged less than 12 years, the mother will be invited to provide written consent for herself and the child. The informed consent to participate in the study must be obtained by the investigator, or a person designated by the investigator, in accordance with the ICH Guidelines for Good Clinical Practice. Written informed consent will be obtained for the child using the HREC approved Participant Information and Consent Form. The parent/guardian will be advised in a timely manner of any new information that may be relevant to their willingness to participate.

#### Monitoring

The Investigator(s)/institution(s) will permit study-related monitoring, audits, HREC review, and direct access to case record forms, source documents and study files at all study sites for monitoring and audit purposes, at reasonable times, during the course of the study and after completion. The Project Manager will undertake an annual visit to at all five study sites to review case record forms, source documents, sample storage and study files. The investigators will participate in regular teleconferences and an annual face-to-face meeting.

#### Adverse event reporting

The investigators are responsible for conduct of the study in accordance with GCP regulations, which includes the recording and reporting of adverse events observed during and after the study. For all adverse events, the severity, onset, duration, determination of seriousness, action taken, any treatment given, outcome, and the investigator’s assessment of the relationship to study procedure will be recorded by the investigator. The most likely physical adverse event will be related to venipuncture. Reaction to venipuncture will be elicited by direct questioning of the mother or the primary caregiver. There is also potential for adverse psychological events associated with anxiety and grief the parents are likely to feel if their child develops islet autoimmunity. Each state has investigator(s) experienced in counselling families about the significance of islet autoimmunity and knowledgeable about the care of children with T1D. The investigators have combined experience in following over 1,000 children for the development of islet autoimmunity over the last 20 years.

### Data handling and record keeping

#### Data protection and confidentiality

All provided biological samples will be labelled with a re-identifiable study number. The electronic study information will be stored in a dedicated, limited access clinical registry developed by the Melbourne eResearch Group of the University of Melbourne. The registry features electronic Case Report Forms and integrated electronic questionnaires for real-time data processing. Key capabilities support for fine grained access control systems with strong data encryption, secure data enclaves with advanced data management as well as secure back-up of the data entered. As such, the registry is compliant with international standards for electronic systems in clinical studies.

## Discussion

The central aim of the ENDIA Study is to identify the gene-environment interactions occurring during prenatal and/or postnatal development that drive the development of islet autoimmunity and T1D in children genetically at-risk of T1D. Recruitment will begin prospectively during pregnancy with 3-monthly follow-up until two years of age then 6-monthly thereafter in an ongoing cohort study. The cohort will be well characterised in terms of demographics, maternal and proband medical history, obstetric history, parturition and anthropometry. An in-depth analysis of maternal and infant nutrition will inform a comprehensive sampling strategy involving the longitudinal collection of blood and DNA for immune and multi-omics analyses, and stool, urine, breast milk, oral cavity, nares and skin samples/swabs for microbiome analysis. The rapid change of the microbiome after birth in relation to prenatal and postnatal exposures, geographical variations and the onset of islet autoimmunity before two years of age underscores the importance of studying the cohort from pregnancy into early childhood with frequent sampling during the first two years of life, and analysis in relation to the environmental, cultural and genetic influences that are likely to be relevant to an Australian population.

Defining modifiable host and environment factors that initiate and/or promote destruction of insulin-producing cells in early life will have enormous implications for individuals with and at risk of T1D and their families, and for the health care system given the annual cost of approximately $5,000 per person with T1D in Australia [104]. In the short term, outcome data generated from this study aims to provide education programs for T1D risk reduction during pre-conception, pregnancy and early infancy in families with children at risk. In the medium term, the translation of data for gene-environment interactions may allow testing of families for T1D susceptibility genes and education about their relative risk of environmental factors according to genotype. In the longer term, outcomes of the ENDIA Study may lead to a means of primary prevention of T1D before the autoimmune process begins.

## Abbreviations

AGEs: Advanced glycation end products; BMI: Body mass index; DHA: Docosahexaenoic acid; ENDIA: Environmental determinants of islet autoimmunity; EPA: Eicosapentaenoic acid; EV: Enterovirus; FDR: First-degree relative; GADAb: Glutamic acid decarboxylase 65 autoantibodies; HLA: Human leukocyte antigen; IA2Ab: Tyrosine phosphatase-like insulinoma antigen autoantibodies; IAA: Insulin autoantibodies; NOD: non-obese diabetic; PGE2: Prostaglandin E2; RV: Rotavirus; T1D: Type 1 diabetes; Treg: Regulatory T cell; tTGAb: Tissue transglutaminase autoantibodies; ZnT8Ab: Beta cell-specific zinc transporter 8 autoantibodies.

## Competing interests

The authors declare that they have no competing interests.

## Authors’ contributions

The ENDIA Study writing group is comprised of MP, JC, MC, PC, WR, AC, TJ and LH. All assume responsibility for the production of this manuscript. The study was conceived by JC and LH. The study was designed by JC, LH, MC, PC and WR. The study Project Manager is MP. State Principal Investigators are JC (SA), MC (NSW), TJ (WA), PC (Vic) and AC (Qld). PB is an additional Principal Investigator. The Associated Investigators are FC, JD, MM, GM, AN and JW. The Significant Collaborators are CD, JF, KN and RS. All authors have read and approved the final manuscript.

## Authors’ information

ENDIA Study Group:

Peter A Baghurst^1^, Simon C Barry^2^, Fergus J Cameron^3^, Jodie M Dodd^4^, Chris Duran^5^, Josephine M Forbes^6^, Maria Makrides^2,7^, Grant Morahan^8^, Karen E Nelson^9^, Alison J Nankervis^10^, Richard O Sinnott^5^, John M Wentworth^11,12^

^1^School of Population Health, University of Adelaide, Adelaide, SA 5005

^2^Discipline of Paediatrics, School of Paediatrics and Reproductive Health, University of Adelaide, Adelaide, SA 5005

^3^Department of Endocrinology and Diabetes and Centre for Hormone Research, Royal Children’s Hospital and Murdoch Children’s Research Institute, Parkville, Victoria 3052

^4^School of Obstetrics and Gynaecology, University of Adelaide, Adelaide, SA 5005

^5^Department of Computing and Information Systems, University of Melbourne, Parkville, Victoria 3010

^6^Mater Medical Research Institute, South Brisbane, Queensland 4101

^7^Women’s and Children’s Health Research Institute, King William Road, North Adelaide, SA, 5006

^8^Centre for Diabetes Research, Western Australian Medical Research Institute, Perth, WA 6000

^9^J. Craig Venter Institute, Rockville, MD 20850 USA

^10^Royal Women’s Hospital, Parkville, Victoria 3052

^11^Royal Melbourne Hospital, Parkville, Victoria 3050

^12^Walter and Eliza Hall Institute, Parkville, Victoria 3050

## Pre-publication history

The pre-publication history for this paper can be accessed here:

http://www.biomedcentral.com/1471-2431/13/124/prepub

## Supplementary Material

Additional file 1 A detailed description of the data collection, sampling procedures and laboratory investigations employed in the ENDIA study. Click here for file

Additional file 2 The ENDIA Infant Feeding Diary developed for this study. Click here for file

Additional file 3 The ENDIA Maternal Lifestyle in Pregnancy questionnaire developed for this study. Click here for file

Additional file 4 The ENDIA Maternal/Infant Lifestyle Postpartum questionnaire developed for this study. Click here for file
