# Supplementary material for: Environmental determinants of islet autoimmunity (ENDIA): a pregnancy to early life cohort study in children at-risk of type 1 diabetes
Source: BMC Pediatr. 2013 Aug 14;13:124. doi: 10.1186/1471-2431-13-124 (PMC3751791; doi:10.1186/1471-2431-13-124)
Supplement: Additional file 1 — A detailed description of the data collection, sampling procedures and laboratory investigations employed in the ENDIA study. [file 1471-2431-13-124-S1.pdf]

ADDITION FILE 1

1. Data collection and sampling procedures

1.1 Demographics and medical history

Demographic information for the mother and father, as well as the proband sibling where applicable, will be collected at the first available opportunity. Documented information will include date of birth, country of birth, ancestry, individual income, highest level of education, employment status, gender, birth weight (if known) and gestation at birth (if known). Socioeconomic class will be scored as previously described [1]. Medical history will be taken using a prompted list of 28 common medical conditions including thyroid disease and autoimmune disorders amongst others. Other significant illnesses, previous hospitalisations and/or allergies will also be recorded. Obstetric history will document past pregnancy history, histories of live births and whether the conception of the participating infant was assisted through reproductive technologies.

1.2 Anthropometry

Accurate height and weight measurements during pregnancy will be taken using calibrated stadiometers and adult scales. For infants less than two year of age, length will be measured using an infantometer or length board as described [2] and weight measured using infant scales. Beyond two years of age, height will be measured using a standing stadiometer and weight using an adult scale. Weight and length/height measurements after birth and BMI after two years will be converted to z-scores [3].

1.3 Venous blood

Blood will be appropriately collected and stored for both immediate and future testing requirements. An overview of the blood sampling strategy is given in Table 1 of the Article with a detailed schematic representation provided in **Additional File Figure 1**. Venous blood will be drawn using winged butterfly blood-sampling system in accordance with the WHO Guidelines [4] with minimum tubing length and appropriate gauge size for the participant (adult or paediatric). Emla® or LMX4® lignocaine 4% cream may be used to reduce discomfort in children. The evacuated blood collection tubes required in ENDIA have been coded A – F:

ADDITION FILE 1

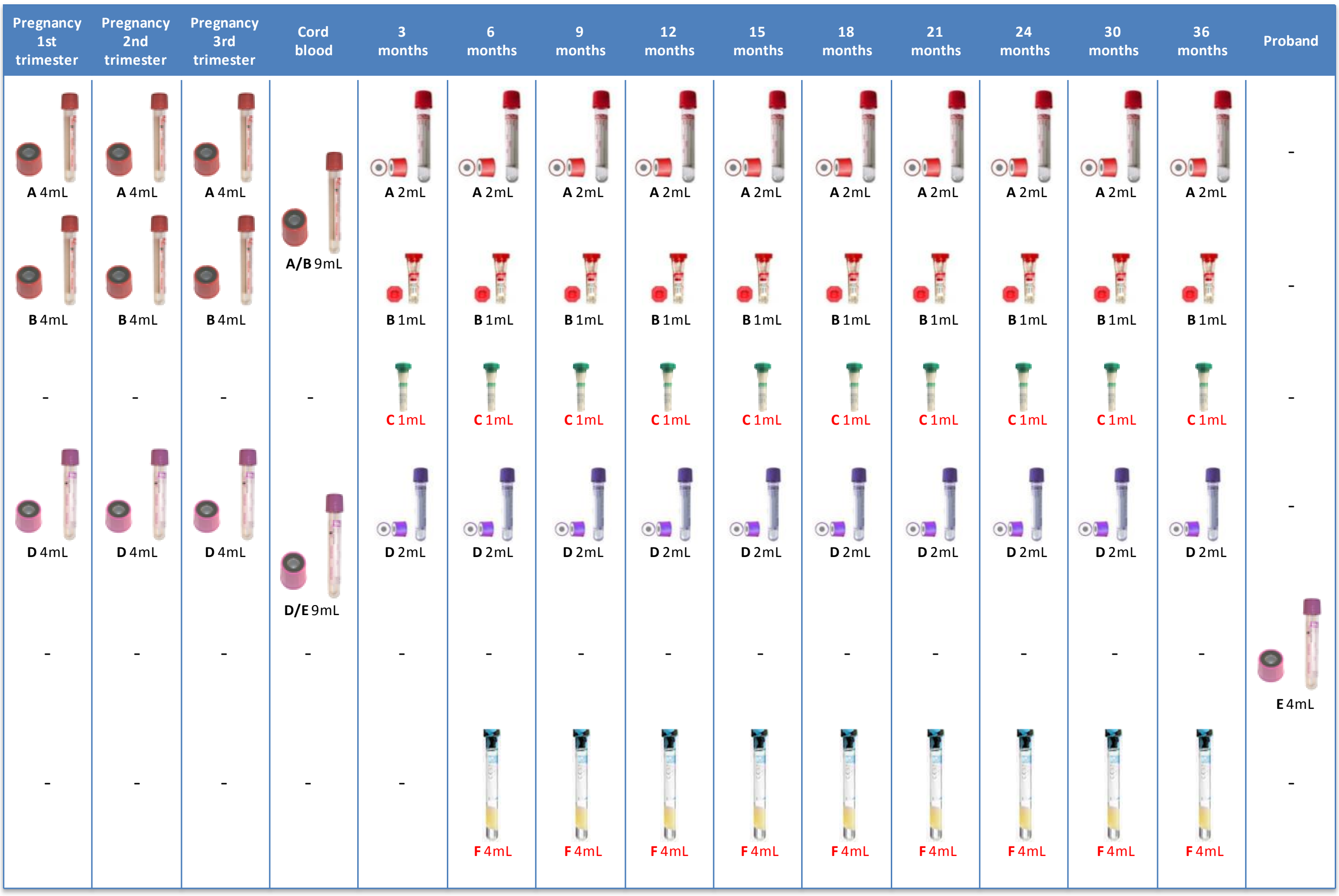

Additional File Figure 1: Schematic representation of the blood tubes collected at each ENDIA study visit.

A. Serum tube to be processed within one hour of collection. Aliquots will be stored at -80°C for batch analysis of islet autoantibodies, adiponectin, hsCRP, insulin and metabolomic studies. Extra aliquots will be stored for purposes yet to be determined.

B. Serum tube to be sent directly to local pathology services for immediate analysis of 25OHD, tissue transglutaminase antibodies (tTGAb), and/or glucose as required for the study visit.

C. Lithium heparin tube for whole blood cytokine and chemokine assays as described previously [5-7].

D. K-EDTA plasma tube to be processed within one hour of collection. Prior to centrifugation, a small aliquot of whole blood may be drawn off for determination of HbA1c using high performance liquid chromatography (HPLC). After centrifugation, plasma will be divided into aliquots and stored at -80°C for viral studies and analysis of CHA, EPA, resolvins, PGE_2 and AGEs. Extra aliquots will be stored for purposes

yet to be determined. Buffy coat will be collected for epigenetic analysis. Erythrocytes will also be collected and stored at -80°C.

E. K-EDTA plasma tube for direct storage of whole blood at -80°C for HLA genotyping.

F. BD Vacutainer® CPT™ Cell Preparation Tube (BD Diagnostics, Dubai) for the isolation of peripheral blood mononuclear cells (PBMCs). The PBMC layer will be washed and counted by hemocytometer before being divided into externally threaded cryovials and stored frozen in liquid nitrogen in an appropriate storage medium. Plasma taken from the CPT tubes, which contain sodium citrate as the anticoagulant, will also be stored for purposes yet to be determined.

1.4 Cord blood

Mixed arterial and venous cord blood will be collected by midwives and/or obstetricians at the time of delivery. A “Cord Blood Collection Kit” containing a 9 mL evacuated serum tube, a 9 mL evacuated K-EDTA plasma tube and standardised instructions for the attending medical professional will be issued to the mother during her third trimester to take with her to the hospital when in labour. After birth, the cord will be clamped with forceps and cut as per standard practice. Whilst the placenta is *in situ*, the forceps on the cord will be loosened allowing the cord blood to flow into a sterile sample pot. The blood will then be transferred to the provided tubes and stored at 4°C until processed.

The 9 mL serum tube will be processed as follows:

- After centrifugation, a 1 mL aliquot of serum will be sent to the local pathology service for immediate analysis of 25OHD, tTGAb and C-peptide (equivalent to Tube B outlined above).
- Remaining serum will be aliqoted and stored at -80°C for batch analysis of islet autoantibodies, adiponectin, hsCRP, insulin and metabolomic studies (equivalent to Tube A outlined above). Extra aliquots will be stored for purposes yet to be determined.

The 9 mL K-EDTA plasma tube should be processed as follows:

- Prior to centrifugation, a small aliquot will be drawn off for determination of HbA1c using HPLC as described for Tube D as outlined above.

- A further 4 mL should be transferred to a clean K-EDTA plasma tube for direct storage of whole blood at -80°C for HLA genotyping (equivalent to Tube F as outlined above).
- After centrifugation the remaining material will be aliquoted and stored at -80°C for viral studies and analysis of DHA, EPA, resolvins, PGE_2 and AGEs. Extra aliquots will be stored for purposes yet to be determined. Buffy coat will be collected for epigenetic analysis. Erythrocytes will also be collected and stored at -80°C. This is equivalent to Tube D outlined above.

Participation in the ENDIA study will not prevent families from storing or donating cord blood through private or public cord blood banks if they wish to do so.

1.5 Saliva

Saliva will be collected for SNP genotyping using Oragene.DNA devices (DNA Genotek, Ontario, Canada). The OG-250 Oragene.DNA Disc Format device will be used for children and the OG-500 Oragene.DNA Tube Format device for adults as per the manufacturer's instructions. Samples are stable at room temperature.

1.6 Swabbing

Swabs for analysis of the microbiome will be collected using ESwab (Copan, Brescia, Italy) as per the manufacturer's recommendations. The ESwab is a certified DNase/RNase free liquid-based collection and transport system successfully used for microbiome studies [8]. Throat swabs for viral studies will be collected using the UTM collection system (Copan). All swabs will be placed on ice immediately following collection and stored at -80°C within one hour.

1.7 Urine

All participants will be provided with an insulated ENDIA cooler bag for the storage and transport of urine, stool and breast milk samples, collected in the participant's home.

During pregnancy, women will be given a "Maternal Urine Collection Kit" consisting of a 70 mL sterile urine jar, a sanitising wipe, gloves and instructions to collect the first morning void.

For infant urine collection, caregivers will be provided with an “Infant Urine Collection Kit” containing a Liv Pedia urine collection bag (Livingston, Rosebery, NSW, Australia), a sterile urine jar, a skin sanitising wipe, gloves and instructions to collect the sample on the morning of the scheduled appointment. Once children are capable of using a toilet the sample can be collected directly in the jar. Participants will be instructed to place the sample inside the ENDIA cooler bag and store the bag in the home refrigerator until the scheduled appointment time. After centrifugation, samples will be stored as aliquots at -80°C for downstream analysis of the metabolome and AGEs. Extra aliquots will be kept for purposes yet to be determined.

1.8 Stool

During pregnancy, maternal stool samples will be collected using the EasySampler collection device (Coverthem Limited, Hampshire, UK). These flushable stool pans have been reported to increase sampling compliance whilst reducing contamination with toilet water [9], which is imperative for studies of the microbiome. Women will be provided with a “Maternal Stool Collection Kit” consisting of an EasySampler device, a faecal collection jar, Ziploc bags, gloves and instructions to collect the sample in the 24 hours prior to the scheduled appointment. Following collection, participants will be advised to double bag the faecal collection jar, place the jar inside the ENDIA cooler bag and store it in the home refrigerator until the scheduled appointment time. This collection method has been implemented successfully for the analysis of the faecal microbiome [10].

In the third trimester visit prior to the baby’s birth, mothers will be provided with a Ziploc bag for collection of the nappy (diaper) containing baby’s first bowel movement, the meconium. For subsequent study visits, caregivers will be provided with an “Infant Stool Collection Kit” containing a faecal collection jar, Ziploc bags, gloves and instructions to scoop a stool sample from the baby’s nappy or potty in the 24 hours preceding the scheduled appointment. As with the maternal stool samples, infant stools should be double bagged, placed inside the ENDIA cooler bag and stored in the home refrigerator until the scheduled appointment time.

All received stool samples will be processed in a Biosafety Level II cabinet or laminar flow hood to reduce potential contamination of the faecal microbiome. Sterile scalpel blades will be used to transfer portions of stool into sterile externally threaded cryovials. Samples will be

stored at -80°C for analysis of the microbiome, metabolome and virology studies. Extra aliquots will be kept for purposes yet to be determined.

1.9 Colostrum and breast milk

The procedure for collecting colostrum and breast milk has been developed so as to be performed independently by mothers. Colostrum will be collected on two occasions within five days of giving birth, first between 0-2 days and then between 3-5 days. Mothers will be provided with a “Colostrum Collection Kit” consisting of a sterile water wipe, a sterile 5 mL syringe, a sterile 5 mL screw-cap jar, gloves and standardised instructions for the hand expression of breast milk using the Marmet technique [11]. Mothers will be requested to wipe the breast area with the wipe to reduce bacteria residing on the skin and allow to air dry. Colostrum will be expressed using gloved hands, drawing droplets directly from the nipple into the syringe and transferring to the jar. Samples should be stored in the refrigerator prior to processing. Breast milk samples will be requested at all subsequent study visits for as long as the infant continues to breast feed. Mothers will be provided with “Breast Milk Collection Kit” containing a sterile water wipe, a sterile 70 mL jar, gloves and standardised instructions to collect the sample on the morning of the study appointment as per the Marmet technique [11]. After cleaning the breast, milk samples should be expressed directly into the sterile jar using gloved hands. Samples will be placed in the ENDIA cooler bag and stored in the home refrigerator until the scheduled appointment time.

Received colostrum/breast milk samples will be processed in a Biosafety Level II cabinet or laminar flow hood to reduce potential contamination of the microbiome. The jar will be inverted five times to re-distribute separated layers prior to aliquoting into sterile screw-cap tubes. Samples will stored at -80°C for analysis of the microbiome and metabolome. Extra aliquots will be kept for purposes yet to be determined.

1.10 Nutrition

No systematic dietary advice will be given either during the pregnancy or follow-up after birth. The assessment of maternal/infant nutrition will be in five phases:

1. During pregnancy, at the third trimester the mother will complete a DQESv2 food frequency questionnaire [12]. The questionnaire comprises a food list of 74 items divided in four categories: (i) cereal foods, sweets and snacks; (ii) dairy products,

meats and fish; (iii) fruit, and (iv) vegetables. A separate set of questions covers intake of alcoholic beverages. Questionnaires will be analysed by the Cancer Council of Victoria, Australia. Output data will include: water, energy, fat (total), protein, carbohydrate (total), sugars, starch and dextrins, fibre, cholesterol, sodium, potassium, calcium, phosphorus magnesium, iron, zinc, retinol equivalent, retinol, thiamine, beta-carotene equivalent, riboflavin, niacin, niacin equivalent, vitamin C, alcohol, total saturated fatty acids, total monounsaturated fatty acids, total polyunsaturated acids, individual fatty acids, carotenoids, glyacemic index and glycaemic load.

2. During the first 12 months of life, the mother/caregiver will complete a weekly feeding diary developed specifically for the ENDIA study (ENDIA Infant Feeding Diary: **Additional file 2**). The diary will record: (i) feeding at birth, (ii) duration of exclusive breastfeeding and maternal antibiotic usage during lactation, (iii) age at introduction of cow's milk-based infant formulae, (iv) commercial brand of infant formulae, (v) exposure to drinks other than milk and water including fruit and vegetable juices, (vi) exposure to solid foods including the types/brands of foods and amounts consumed, (vii) food and beverage heating/cooking methods, (viii) antibiotic, supplement and other medication usage in the infant, (ix) vaccinations in the infant, and (x) general health of the infant including fevers. Diaries will be checked by the Research Nurses and discussed during the study appointments at the three, six, nine and 12 month visits. A standardised data capture form integrated into the ENDIA clinical registry will be used to extract information on first exposures to vegetables, fruit, eggs, diary, wheat, rice, soy, corn, oats, barley, nuts, bovine meat, non-bovine meat and fish/shellfish. Subsequent in-depth analysis of the diaries will calculate kcals, trace elements and vitamin intake as well as AGEs content.
3. If the mother is breastfeeding she will complete another DQESv2 at three months to evaluate maternal nutrition during lactation.
4. From 12 months, the mother will complete three multi-pass 24 hour food recall interviews for the infant every three (maximum) to six (minimum) months as described elsewhere [13]. The food recall data measures intake of kcals, trace elements and vitamins. Ideally, the recalls will be taken to cover two weekdays and one weekend day. One of the three recalls will be conducted face-to-face with the Research Nurse during a study visit, and the other two via telephone.
5. Once the child is aged 24 months, the Australian Toddler Eating Survey version 1 [14] will be completed by the mother at the 24, 30 and 36 months visits.

1.11 Lifestyle and physical activity

The lifestyle questionnaire will capture aspects of the maternal diet not evaluated by the DQESv2, including caffeine consumption, supplement usage, antibiotic usage in pregnancy, smoking, household residents, household pets, infant sleeping arrangements and infant socialisation. This questionnaire will be offered at all visits during pregnancy to monitor the foetal environment (ENDIA Pregnancy Lifestyle Questionnaire: **Additional File 3**) and after birth (ENDIA Postnatal Lifestyle Questionnaire: **Additional File 4**) to capture potential environmental changes relevant to the development of islet autoimmunity and/or T1D.

The Pregnancy Physical Activity Questionnaire (PPAQ), a validated and published questionnaire [15], will evaluate maternal physical activity during pregnancy measured as "metabolic equivalents" (MET) per week. The questionnaire will be completed at each trimester.

2. Laboratory investigations

2.1 Genomic DNA

DNA will be extracted from cord blood with a Qiagen HLA typing kit. PBMCs (~1 million/ml blood) will be stored from each visit in 'RNAlater' (Ambion, Life Technologies, Carlsbad, CA) and RNA purified with a Picopure RNA kit (Applied Biosystems, Life Technologies). HLA-A and B (class I) and class II typing will be performed by nucleotide sequencing [16]. DNA will be typed for the 50 SNPs most highly associated with T1D, including IDDM2/insulin -23Hph1, as reported by the T1D Genetics Consortium [17], as well as for CYP27B1 and VDR genes.

2.2 Autoantibodies

Autoantibodies to insulin (IAA), glutamic acid decarboxylase 65 (GADAb), tyrosine phosphatase-like insulinoma antigen (IA2Ab), beta cell-specific zinc transporter 8 (ZnT8Ab) and tissue transglutaminase (tTGAb) will be measured. Transient autoantibodies detected in infants less than 9 months of age when the mother has T1D, attributable to transplacental transfer, will not be defined as islet autoimmunity

IAA will be measured by fluid phase radiobinding assay as described [18] with an inter-assay CV <15%, and will include a measure of binding affinity. High IAA affinity has been strongly associated with both progression to multiple antibodies and progression to T1D [19].

ZnT8Ab [20] will be measured by precipitation of ^{35}S-methionine labelled recombinant human ZnT8 protein. Results will be expressed in arbitrary units in comparison to positive and negative controls. The assay had a sensitivity 64% and specificity 100% in the 2010 Diabetes Autoantibody Standardization Program (DASP) serum exchange.

In previous studies, our research team has measured GADAb and IA2Ab by precipitation of ^{35}S-methionine-labelled recombinant human GAD65 and IA2 [18, 21]. In the 2010 DASP, the GADAb and IA2Ab assays scored 82% and 64% sensitivity and 99% and 100% specificity, respectively. We have recently implemented an ELISA for measuring GADAb and IA2Ab (RSR Ltd, Cardiff, UK). GADAb and IA2Ab assays scored 87% and 88% for sensitivity and 99% and 100% for specificity, respectively (unpublished data). tTGAb will be measured at each participating site using test-specific thresholds for antibody positivity.

2.3 Microbiome

Microbiome analysis of longitudinal samples in pregnancy and childhood will be performed on stool, throat, buccal, gingival, vaginal, colostrum and breast milk at the J. Craig Venter Institute, Gaithersberg, MD, USA. 16S rDNA sequencing will be performed using a standard high-throughput 16S PCR amplification pipeline and 454 GS FLX + sequencing. 96 samples per full run of 454 will generate an average of 10,000 reads per sample. With current 454 sequencing capacity of 1 million reads per run (or 500,000 reads/half run), we anticipate generating up to 10,000 16S sequences for each sample. To accommodate this PCR will be performed on all samples for the targeted 16S region. For metagenomic sequencing, the HiSeq 2000 sequencing platform (Illumina, San Diego, CA) will be used producing up to 4 Gbp of sequence per sample. One lane of Hi-seq produces roughly 40Gbp of data, yielding 8Gbp/sample. Assuming that average genome size of a bacterial species is 4Mbp, this amount of sequencing will provide ~2000x coverage of sequence data per genome on average (8Gbp/4Mbp = 2000).

The metagenomic study of the human virome is hampered by (i) viruses accounting for a very small fraction of the human microbiota, and (ii) a lack of conserved genes across all viral species to evaluate viral diversity, analogous to 16S rDNA bacterial profiling. To

improve viral detection sensitivity we will purify virion particles using published protocols [22] for in-depth sequence analysis. Specifically, this approach will target both DNA/RNA viruses and also select low abundance viral species and facilitate isolation of full length or nearly full length unclassified or novel viral species. We will then apply random hexamer priming and amplification [23] for direct amplification of genetic material from the sample and a hybrid sequencing strategy using both 454 and Illumina-HiSeq platforms for in-depth viral sequence analysis.

2.4 Insulin resistance

The HOMA measure of insulin resistance will be calculated as: [(fasting glucose x fasting insulin) / 22.5]. As neither HOMA nor the alternative quantitative insulin sensitivity check index (QUICKI) are clearly superior measures of insulin sensitivity compared to fasting insulin alone in children [24] both fasting insulin and HOMA will be used in the analysis. Total and high molecular weight serum adiponectin will be measured using an ELISA (ALPCO diagnostics, Salem, NH) as we have described [25].

2.5 Inflammatory mediators

The total phospholipid fraction of the lipid extract from erythrocytes will be separated by thin-layer chromatography and methylated. Fatty acid methyl esters will then be separated and quantified by capillary gas chromatography [26]. Prostaglandin E2 will be measured using stable isotope dilution HPLC tandem mass spectrometry at the South Australia Neonatal Screening Centre. 25OHD will be measured by local pathology services using the laboratory’s standard method. High sensitivity C-reactive protein (hsCRP) will be measured by a near infrared particle immunoassay method with IMMAGE Immunochemistry Systems Reagent (Beckman Coulter Inc, CA). For analysis of AGEs, carboxymethyllysine will be measured in urine and plasma by HPLC and by in-house ELISA [27]. Methylglyoxal will be measured by HPLC. Soluble receptor for AGEs (RAGE) will be measured by ELISA.

2.6 Treg subsets and immune function

Cytokines and chemokines will be measured on small volumes of plasma by Luminex bead assay before and after incubation of heparinised blood in the absence or presence of Toll-like receptor 2 (Pam-3-Cys), 3 (poly I:C), 4 (lipopolysaccharide-LPS), 5 (flagellin) and 9 (CpG DNA) agonists, a T-cell receptor agonist monoclonal Ab OKT3, and the T-cell antigens

Diptheria Pertussis Tetanus Toxoid, proinsulin and GAD65, as previously described [5-7]. Analytes will include reported markers of inflammation in T1D (IL-1β, TNF-α, IL-6). Treg cell subsets will be defined in PBMCs as described previously [28], using multi parameter (six colour) flow cytometry.

2.7 Viral infection

DNA/RNA will be extracted from plasma samples using the automated Kingfisher System (Thermo Scientific, Waltham, MA). The 5´ UTR and VP1 genes of the EV genome will be amplified by nested RT-PCR using established methods [29, 30] and samples will also be analysed by multiplex PCR to detect other common viruses including Cytomegalovirus (CMV), Epstein-Barr virus (EBV), Herpes Simplex virus (HSV) and Varicella Zoster virus (VZV) [31, 32]. Stool samples will be tested for EV, RV and Norovirus. Positive samples are repeated for confirmation and PCR products bi-directionally sequenced with an ABI 3730 DNA analyser using BigDye terminator chemistry (Applied Biosystems, Life Technologies). Phylogenetic analysis will be carried out on the 5'UTR and VP1 sequences of EV PCR-positive strains to determine virus genotypes [33], sequences will be submitted to Genebank and compared with published sequences and Australian isolates [33, 34]. The multiplex PCR assay will also be used to test for viruses in neonates using newborn screening cards, as we have described [35]. Nucleic acids will be extracted from the blood spot (Qiagen, Hilden, Germany) and samples processed using the same multiplex PCR method. Serum samples will be tested for serological evidence of infection for EV, HSV, VZV, EBV, CMV and RV.

3. References

1. **Census of Population and Housing: Socio-Economic Indexes for Areas (SEIFA), Australia - Data only , 2006** [http://www.abs.gov.au/AUSSTATS/abs@.nsf/DetailsPage/2033.0.55.0012006]
2. Shorr IJ: **How to Weigh and Measure Children**. New York: United Nations, Dept. of Technical Co-operation for Development and Statistical Office; 1986.
3. **CDC growth charts: United States** [http://www.cdc.gov/growthcharts/]
4. World Health Organisation: **WHO guidelines on drawing blood: best practices in phlebotomy**. Geneva, Switzerland: WHO Document Production Services; 2010.
5. Petrovsky N, Harrison LC: **Diurnal rhythmicity of human cytokine production: a dynamic disequilibrium in T helper cell type 1/T helper cell type 2 balance?** *J Immunol* 1997, **158**(11):5163-5168.
6. Petrovsky N, Kyvik KO, Bonnevie-Nielsen V, Beck-Nielsen H, Green A, Harrison LC: **Evidence from twins for acquired cellular immune hyperactivity in type 1 diabetes**. *Immunology* 2002, **106**(4):584-589.
7. Antonelli A, Fallahi P, Ferrari SM, Pupilli C, d'Annunzio G, Lorini R, Vanelli M, Ferrannini E: **Serum Th1 (CXCL10) and Th2 (CCL2) chemokine levels in children**

with newly diagnosed Type 1 diabetes: a longitudinal study. *Diabetic Medicine* 2008, **25**(11):1349-1353.
8. Bai G, Gajer P, Nandy M, Ma B, Yang H, Sakamoto J, Blanchard MH, Ravel J, Brotman RM: **Comparison of storage conditions for human vaginal microbiome studies**. *PLoS One* 2012, **7**(5):e36934.
9. Ahlquist DA, Schwartz S, Isaacson J, Ellefson M: **A stool collection device: the first step in occult blood testing**. *Ann Intern Med* 1988, **108**(4):609-612.
10. Partty A, Kalliomaki M, Endo A, Salminen S, Isolauri E: **Compositional development of Bifidobacterium and Lactobacillus microbiota is linked with crying and fussing in early infancy**. *PLoS One* 2012, **7**(3):e32495.
11. Marmet C: **Manual expression of breast milk using the Marmet technique**. In. Edited by The Lactation Institute. Encino, CA; 2003.
12. Hodge A, Patterson AJ, Brown WJ, Ireland P, Giles G: **The Anti Cancer Council of Victoria FFQ: relative validity of nutrient intakes compared with weighed food records in young to middle-aged women in a study of iron supplementation**. *Aust N Z J Public Health* 2000, **24**(6):576-583.
13. Hendrie GA, Golley RK: **Changing from regular-fat to low-fat dairy foods reduces saturated fat intake but not energy intake in 4-13-y-old children**. *Am J Clin Nutr* 2011, **93**(5):1117-1127.
14. Duncanson K, Burrows T, Collins C: **Study protocol of a parent-focused child feeding and dietary intake intervention: the feeding healthy food to kids randomised controlled trial**. *BMC Public Health* 2012, **12**:564.
15. Chasan-Taber L, Schmidt MD, Roberts DE, Hosmer D, Markenson G, Freedson PS: **Development and validation of a Pregnancy Physical Activity Questionnaire**. *Med Sci Sports Exerc* 2004, **36**(10):1750-1760.
16. Hemminki K, Li X, Sundquist J, Sundquist K: **Familial association between type 1 diabetes and other autoimmune and related diseases**. *Diabetologia* 2009, **52**(9):1820-1828.
17. Barrett JC, Clayton DG, Concannon P, Akolkar B, Cooper JD, Erlich HA, Julier C, Morahan G, Nerup J, Nierras C *et al*: **Genome-wide association study and meta-analysis find that over 40 loci affect risk of type 1 diabetes**. *Nature Genetics* 2009, **41**(6):703-707.
18. Colman PG, Steele C, Couper JJ, Beresford SJ, Powell T, Kewming K, Pollard A, Gellert S, Tait B, Honeyman M *et al*: **Islet autoimmunity in infants with a Type I diabetic relative is common but is frequently restricted to one autoantibody**. *Diabetologia* 2000, **43**(2):203-209.
19. Achenbach P, Lampasona V, Landherr U, Koczwara K, Krause S, Grallert H, Winkler C, Pfluger M, Illig T, Bonifacio E *et al*: **Autoantibodies to zinc transporter 8 and SLC30A8 genotype stratify type 1 diabetes risk**. *Diabetologia* 2009, **52**(9):1881-1888.
20. Wenzlau JM, Moua O, Sarkar SA, Yu L, Rewers M, Eisenbarth GS, Davidson HW, Hutton JC: **SlC30A8 is a major target of humoral autoimmunity in type 1 diabetes and a predictive marker in prediabetes**. *Ann N Y Acad Sci* 2008, **1150**:256-259.
21. Couper JJ, Beresford S, Hirte C, Baghurst PA, Pollard A, Tait BD, Harrison LC, Colman PG: **Weight gain in early life predicts risk of islet autoimmunity in children with a first-degree relative with type 1 diabetes**. *Diabetes Care* 2009, **32**(1):94-99.
22. Allen LZ, Ishoey T, Novotny MA, McLean JS, Lasken RS, Williamson SJ: **Single virus genomics: a new tool for virus discovery**. *PLoS One* 2011, **6**(3):e17722.
23. Djikeng A, Halpin R, Kuzmickas R, Depasse J, Feldblyum J, Sengamalay N, Afonso C, Zhang X, Anderson NG, Ghedin E *et al*: **Viral genome sequencing by random priming methods**. *BMC Genomics* 2008, **9**:5.

24. Cutfield WS, Hofman PL: **Simple fasting methods to assess insulin sensitivity in childhood**. *Horm Res* 2005, **64 Suppl 3**:25-31.
25. Pena AS, Belobrajdic DP, Wiltshire E, Gent R, Hirte C, Couper J: **Adiponectin relates to smooth muscle function and folate in obese children**. *Int J Pediatr Obes* 2010, **5**(2):185-191.
26. Makrides M, Hawkes JS, Neumann MA, Gibson RA: **Nutritional effect of including egg yolk in the weaning diet of breast-fed and formula-fed infants: a randomized controlled trial**. *American Journal of Clinical Nutrition* 2002, **75**(6):1084-1092.
27. Coughlan MT, Forbes JM: **Temporal increases in urinary carboxymethyllysine correlate with albuminuria development in diabetes**. *Am J Nephrol* 2011, **34**(1):9-17.
28. Sadlon TJ, Wilkinson BG, Pederson S, Brown CY, Bresatz S, Gargett T, Melville EL, Peng K, D'Andrea RJ, Glonek GG *et al*: **Genome-wide identification of human FOXP3 target genes in natural regulatory T cells**. *J Immunol* 2010, **185**(2):1071-1081.
29. Craig ME, Howard NJ, Silink M, Rawlinson WD: **Reduced frequency of HLA DRB1*03-DQB1*02 in children with type 1 diabetes associated with enterovirus RNA**. *J Inf Dis* 2003, **187**(10):1562-1570.
30. Craig ME, Robertson P, Howard NJ, Silink M, Rawlinson WD: **Diagnosis of enterovirus infection by genus-specific PCR and enzyme-linked immunosorbent assays**. *J Clin Microbiol* 2003, **41**(2):841-844.
31. Chow SS, Craig ME, Jones CA, Hall B, Catteau J, Lloyd AR, Rawlinson WD: **Differences in amniotic fluid and maternal serum cytokine levels in early midtrimester women without evidence of infection**. *Cytokine* 2008, **44**(1):78-84.
32. McIver CJ, Jacques CF, Chow SS, Munro SC, Scott GM, Roberts JA, Craig ME, Rawlinson WD: **Development of multiplex PCRs for detection of common viral pathogens and agents of congenital infections**. *J Clin Microbiol* 2005, **43**(10):5102-5110.
33. Nolan MA, Craig ME, Lahra MM, Rawlinson WD, Prager PC, Williams GD, Bye AM, Andrews PI: **Survival after pulmonary edema due to enterovirus 71 encephalitis**. *Neurology* 2003, **60**(10):1651-1656.
34. Sanders SA, Herrero LJ, McPhie K, Chow SS, Craig ME, Dwyer DE, Rawlinson W, McMinn PC: **Molecular epidemiology of enterovirus 71 over two decades in an Australian urban community**. *Arch Virol* 2006, **151**(5):1003-1013.
35. Howard J, Hall B, Brennan LE, Arbuckle S, Craig ME, Graf N, Rawlinson W: **Utility of newborn screening cards for detecting CMV infection in cases of stillbirth**. *J Clin Virol* 2009, **44**(3):215-218.
