# Supplementary material for: Environmental determinants of islet autoimmunity (ENDIA): a pregnancy to early life cohort study in children at-risk of type 1 diabetes
Source: BMC Pediatr. 2013 Aug 14;13:124. doi: 10.1186/1471-2431-13-124 (PMC3751791; doi:10.1186/1471-2431-13-124)
Supplement: Additional file 2 — The ENDIA Infant Feeding Diary developed for this study. [file 1471-2431-13-124-S2.pdf]

Affix Participant Clinical Label Here

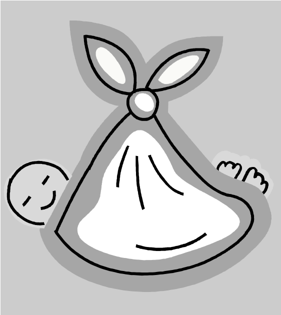

ENDIA

environmental determinants of islet autoimmunity

Infant Feeding Diary

Birth – 6 months

ENDIA: ENVIRONMENTAL DETERMINANTS OF ISLET AUTOIMMUNITY

INFANT FEEDING DIARY

Principal Investigator: Prof. Jenny Couper
University Department of Paediatrics • Women's and Children's Hospital
North Adelaide, SA 5006
Phone +61 8 8161 6242 • Fax +61 8 8161 7759

Table of Contents

About the ENDIA study 1

Instructions on completing the ENDIA Feeding Diary 2

Week-by-week feeding records 7

Contacting the ENDIA study staff 164

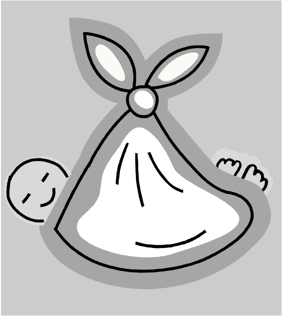

About the ENDIA study

Thank you to you and your baby for participating in the ENDIA study – Australia's largest study into understanding the causes of Type 1 Diabetes

The ENDIA study is looking into what contributes to the development of Type 1 Diabetes so we can find ways to prevent it. Type 1 Diabetes in children is twice as common as it was 20 years ago. This is because our environment has changed and at-risk children are more likely to develop Type 1 Diabetes.

If we can understand exactly what in the environment is harmful or protective, we can modify the environment to try and prevent Type 1 Diabetes. We already know that a child's genes can increase their risk of developing Type 1 Diabetes. However, we think that there are a number of things in the environment that make Type 1 Diabetes more likely to happen in children who have these genes that increase their risk for the disease.

We are interested in the foods that babies consume over the first 26 weeks (6 months) of life to see whether any specific foods or feeding patterns make it more or less likely that an at-risk child could develop Type 1 Diabetes.

By completing this food diary you are making a very important contribution to Type 1 Diabetes research in Australia.

Instructions on completing the ENDIA Infant Feeding Diary

IMPORTANT: This Infant Feeding Diary is not intended to provide you or your family with nutritional advice for your baby. We simply want to capture dietary information about your child.

Any foods that are listed within this Infant Feeding Diary should be considered as examples only, not a guideline as to when a particular food should be introduced.

If you have any questions regarding your baby's diet please speak to your child health nurse, doctor or other professional.

Answer each question as best you can. Estimate if you are not sure – a guess is better than not answering a question that does apply to you! Otherwise, we will consider that your baby did not receive that item in question at all.

If your baby is regularly cared for by someone else, it is important that you ask this person/people to give you the information to answer the questions.

We recommend you complete the Infant Feeding Diary on the same day each week. For example, on the same day your baby was born. This will help you to remember to complete the Infant Feeding Diary. If you do forget for a week or two, please go back and answer the questions, doing your best to remember what foods and drinks your baby was given. The completion date at the top of each week refers to the date you completed the questions.

Complete the tick boxes like this: ☑
Do not place crosses in boxes that don't apply to your baby. Instead, leave the answer blank.

How to answer specific questions

Footnote A: Instructions for Question 1

For the following question:

1. *In the past 7 days, how often was your baby feed each item listed below on average? Consider everyone that has feed the baby, even hospital staff, and include snacks and night-time feeds.*

		Number feeds per day	*Number of days given*	*Not given*
1.1	*Breast milk via breast feeding*	_____	_____	☐
1.2	*Expressed breast milk*	_____	_____	☐
1.3	*Formula*	_____	_____	☐
1.4	*Water*	_____	_____	☐
...	*...*	*...*	*...*	*...*

Please indicate the **average** number of times your baby was given the item each day, and the number of days you baby received the item over the last week. Complete both columns.

For example, if your baby had an average of 8 breast feeds per day every day this week, you would write "8" in the first column and "7" in the second column of the "Breast milk via breast

feeding" row. If you baby received expressed breast milk once on two different days this week, you would write "1" in the first column and "2" in the second column of the "Expressed breast milk in a bottle" row.

If your baby did not receive the item listed, tick the box in the third column.

Footnote B: Instructions for Question 4

For the following question:

4. *The following question relates to occasions when your baby consumed drinks other than breast milk, formula or plain water. This includes cow's milk or milk from other non-dairy sources as well as fruit juices, vegetable juices, cordials, soft drinks, etc. Please state what the drink(s) were, the brand of the product (where applicable) and how much your baby consumed on occasions these drinks were provided. Drink additives should also be included*

Examples of your answers may include:

Pura full fat milk – 100 mL<br>Freshly squeezed orange juice (homemade) – 50 mL<br>Golden Circle Junior Fruit Apple Juice – 150 mL<br>Banana smoothie (at café) – 200 mL<br>Milo made with Pura full fat milk – 2 teaspoons of Milo in 200 mL of milk

In this question, please include any drink additives that you baby may have received this week. For example, Aptamil® Feed Thickener, Nestle Milo, Bickford's Strawberry Milk Mix, etc. Provide as much information about the drinks and/or drink additives as you can. By providing complete product names, including the brand, we can easily look up the nutritional information about this drink or drink additive. By indicating how much your baby drank, we can accurately measure the nutrients he/she has consumed.

Footnote C: Instructions for Question 5

For the following part of Question 5:

5. *The following questions relate to occasions when your baby consumed solid foods this week. Please indicate which of the follow foods your baby received and, on each occasion your baby was offered that food, the average amount he/she consumed with respect to a "spoon" or "mound" code as indicated in the Instructions.*

If your baby received a mixture of foods listed in the provided table (for example, pureed vegetables and fruits mixed together), indicate that your baby consumed both "vegetables" and "fruit". **Please remember to check the ingredients of commercial baby foods as many savoury flavours still contain fruit such as apple.**

Please use the "spoon" and "mound" codes as shown on the next two pages. These diagrams show life-sized representations of serving sizes. If, for example, the "mound" your baby consumed was more than M4 but less than M5, please indicate "M4 + M3" in the space provided. **For food mixtures, estimate the quantity that corresponded to each food in the mixture using the measurements**. Please try to estimate all foods using these measurement codes, even for things such as food sachets and biscuits.

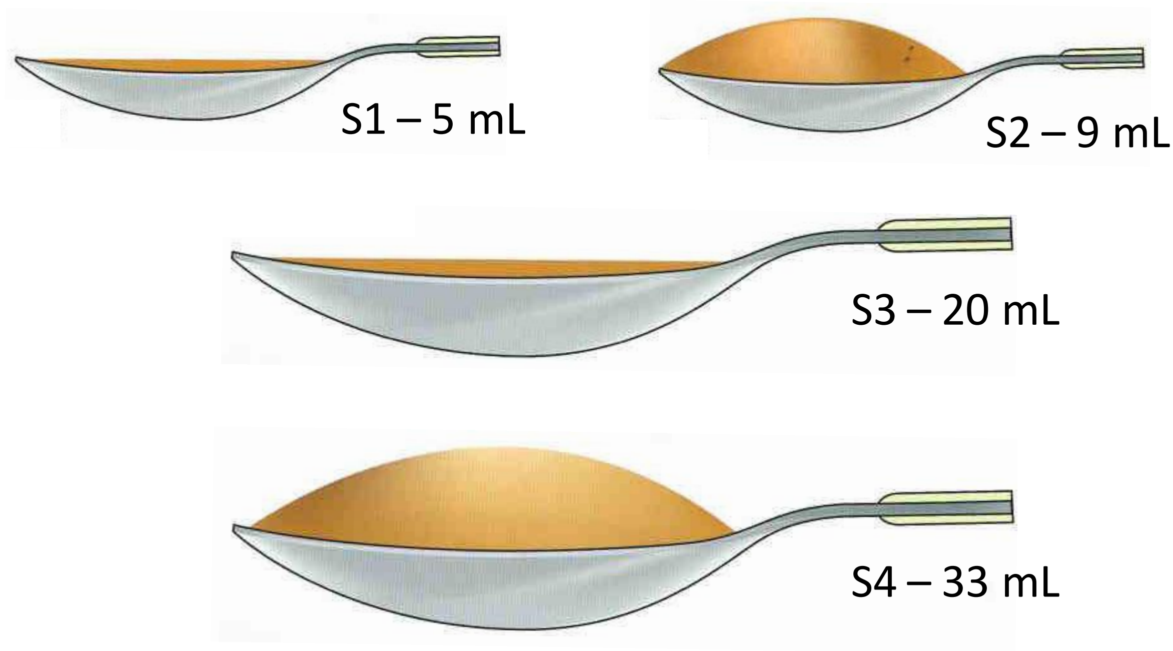
S1 – 5 mL
S2 – 9 mL
S3 – 20 mL
S4 – 33 mL

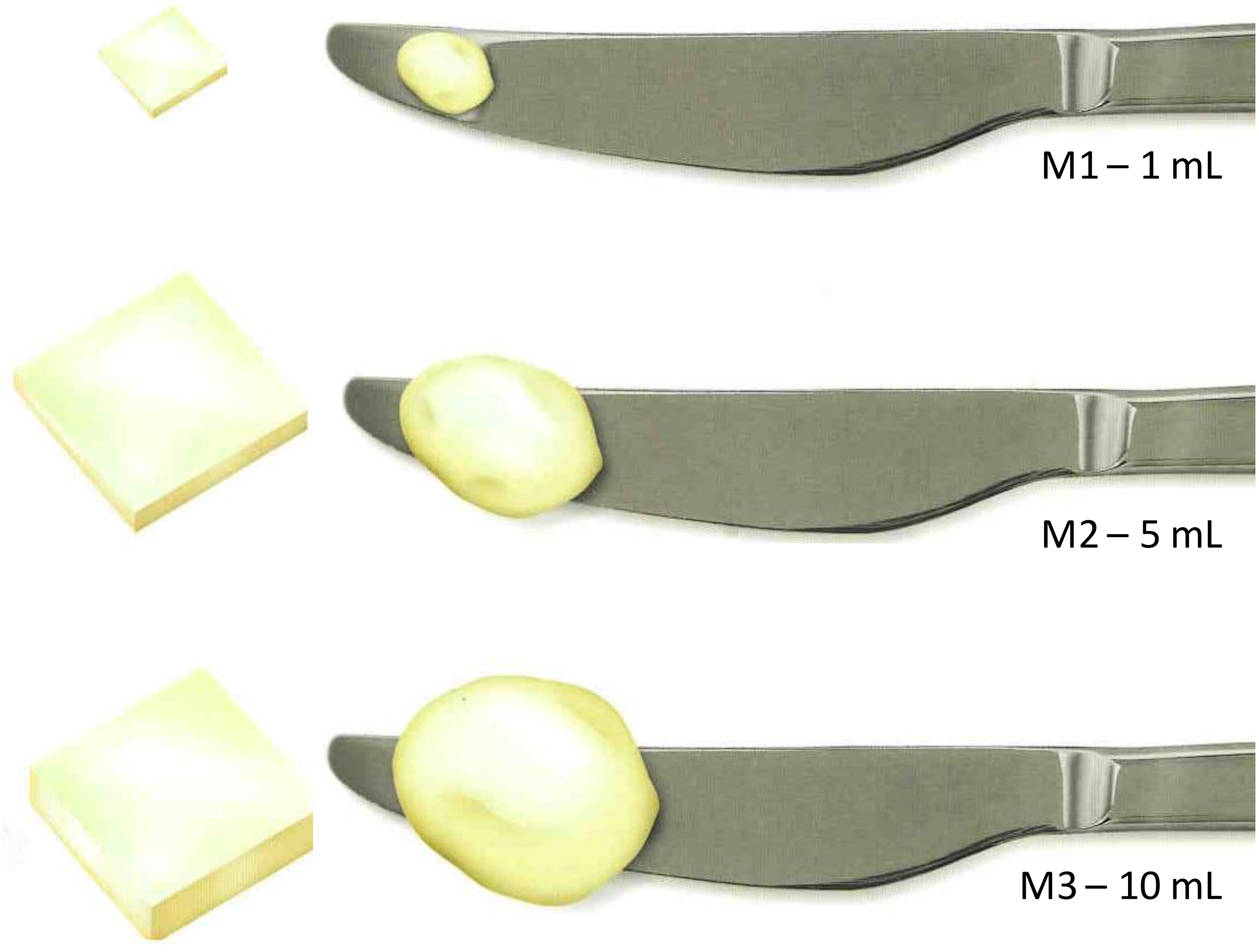
M1 – 1 mL
M2 – 5 mL
M3 – 10 mL

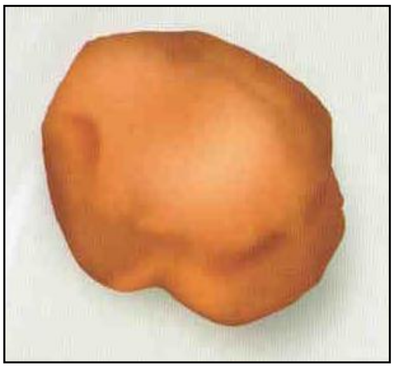

M4 – 1 tablespoon

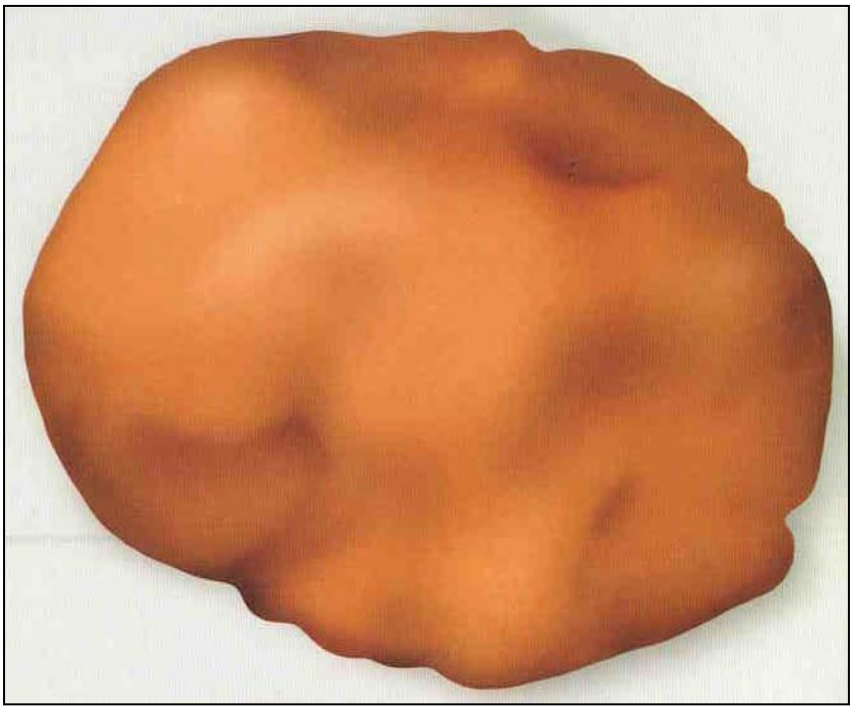

M7 – ¾ cup

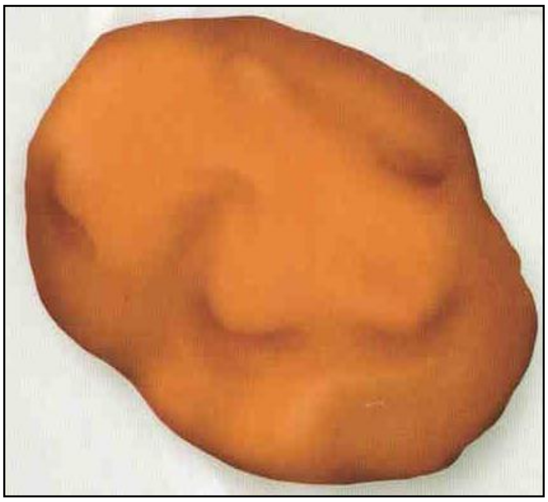

M5 – ¼ cup

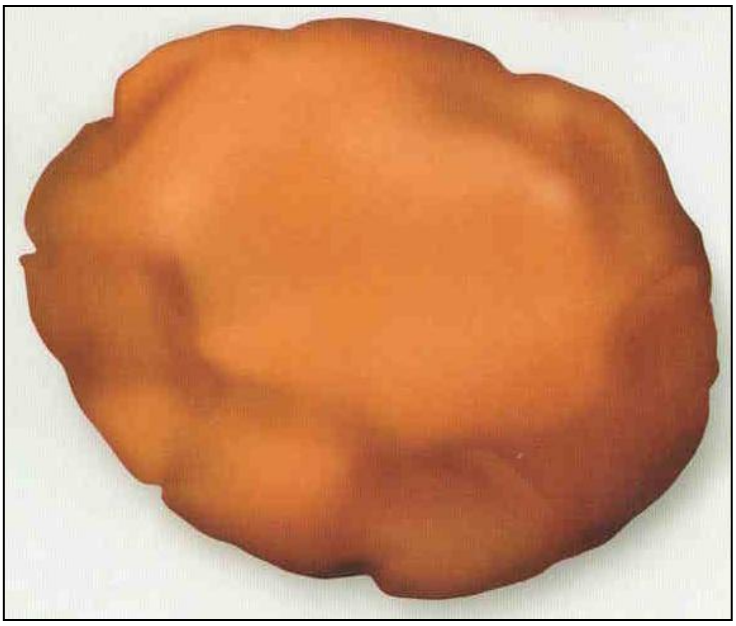

M6 – ½ cup

Footnote D: Instructions for Question 5

For the following part of Question 5:

If your baby consumed any of the solid foods outlined above, please provide some additional information about what those foods were. This may include the name of the food, the brand of the product (where applicable) and whether the food was modified (for example: low-fat, added calcium, wholemeal etc.). Please also list any foods your baby consumed that do not fall in the above categories.

Examples of your answers may include:

Vegetables/fruit: pureed pumpkin; Rafferty's Garden spinach/apple/broccoli/pea sachet<br>Dairy: Dairy Farmers full fat Vanilla yoghurt<br>Wheat: WonderWhite Hi Fibre white bread without crust<br>Rice: Baby Mum Mum vegetable rice rusk<br>Meat: Chicken<br>Unlisted foods: Rabbit

Footnote E – Instructions for Question 7

7. *The following questions relate to occasion when your baby was given supplements and medications. Please state the brand, what the volume of a "dose" was (e.g. 5 mL, 1 tablet, etc.), how many doses were given each day and on how many days it was given this week.*

The following are examples of medication/supplements that should be and need not be included when considering this question. NOTE: any medication not on the exclusion list (even if it is not specified on the "include" list) should also be recorded:

Exclude	Include
• Paracetomol (e.g. Panadol, Dymadon)<br>• Ibuoprofen (e.g. Nurofen, Advil)<br>• Teething gel (e.g. Bonjela)<br>• Cough mixture<br>• Medicated nappy rash cream (e.g. Bepathen, Daktozin)<br>• Colic relievers (e.g. Infants' Friend, Infant Calm)<br>• Homeopathic remedies	• Any doctor prescribed medications (excluding those listed left)<br>• All antibiotics whether they were prescribed by a doctor or not<br>• Fibre supplements<br>• Vitamin supplements (e.g. Pentavite, Vita-Gummies)

In the column labelled "Days", please include the number of days your child was given the supplement/medication <u>THIS WEEK</u>. For example, if your child took some medication on the last two days of this week, but will continue to be given the medication for the next three days of next week, write "2" in the "Days" column for this week and "3" in the column for next week.

Your baby is 1 week old

Completion date: ____/____/____

0. While you were in hospital or birthing centre did your baby consume any of the following:

		Yes	No	Unsure
0.1	Breast milk	☐	☐	☐
0.2	Formula	☐	☐	☐
0.3	Water	☐	☐	☐
0.4	Glucose containing solution[ϕ]	☐	☐	☐

ϕ Includes oral glucose solutions for pain relief and/or sugar water

1. In the past 7 days, how often was your baby feed each item listed below on average? Consider everyone that has feed the baby, even hospital staff. Include snacks and night-time feeds.[A]

		Number feeds per day	Number of days given	Not given
1.1	Breast milk via breast feeding	_____	_____	☐
1.2	Expressed breast milk	_____	_____	☐
1.3	Formula	_____	_____	☐
1.4	Water	_____	_____	☐
1.5	Sugar water	_____	_____	☐
1.6	Cow's milk*	_____	_____	☐
1.7	Other milk**	_____	_____	☐
1.8	Fruit juices	_____	_____	☐
1.9	Other drinks[‡]	_____	_____	☐

* Includes plain, low-fat, evaporated and flavoured varieties of cow's milk. Include milk added to other foods such as cereal.

** Such as soy, rice, almond or goat milk. Include milk added to other foods such as cereal.

‡ Includes vegetable juice, soft drinks, cordial, sports drinks, yoghurt drinks, tea, coffee, etc.

2. The following questions relate to occasions when your baby consumed **breast milk**.

☐ *My baby has never consumed breast milk (proceed to Question 3).*

2.1	Average feed duration when being breast feed:<br>_____________minutes
2.2	Average volume *consumed* when provided expressed breast milk:<br>_____________mL<br>☐ Never provided expressed breast milk *(proceed to Question 3)*

[A] Refer to "Footnote A: Instructions for Question 1" on Page 2 for further clarification about answer this question

2.3	How long was expressed breast milk stored before it was fed to your baby (tick all that applies)?<br>☐ Kept at room temperature ☐ 1 day or less in refrigerator<br>☐ 2-3 days in refrigerator ☐ 3-4 days in refrigerator<br>☐ 5-7 days in refrigerator ☐ Frozen (for any length of time)	
2.4	On the majority of occasions, how were bottles containing expressed breast milk warmed (tick only one)?<br>☐ Did not warm ☐ Hot water[Ψ]\bottle warmer<br>☐ Microwave ☐ Other______________________________	

Ψ When bottle containing the milk is placed into a container of hot water

3. The following questions relate to occasions when your baby consumed **formula**.
   ☐ *My baby has never consumed formula (proceed to Question 4).*

3.1	Average volume consumed with provided formula:<br>______________mL	
3.2	What were the brand(s) and product name(s) of the formula(s) your baby been given. For example, "Wyeth S-26 GOLD Newborn" or "Karicare Aptamil® Delact"<br>1.______________________________<br>2.______________________________<br>3.______________________________<br>☐ Unknown	
3.3	Was the formula prepared according to the manufacturer's instructions?<br>☐ Yes<br>☐ No (please provide details of how the formula was prepared)<br>______________________________<br>______________________________<br>______________________________	
3.5	On the majority of occasions, how were bottles containing formula warmed (tick only one)?<br>☐ Did not warm ☐ Prepared with hot water<br>☐ Hot water[Ψ]\bottle warmer ☐ Microwave<br>☐ Other______________________________	

Ψ When bottle containing the milk is placed into a container of hot water

4. The following question relates to occasions when your baby consumed **drinks other than breast milk, formula or plain water**. This includes cow's milk or milk from other non-dairy sources as well as fruit juices, vegetable juices, cordials, soft drinks, etc. Please state what the drink(s) were, the **brand of the product** (where applicable) and how much your baby consumed on occasions these drinks were provided. Drink additives should also be included.[B] *If your baby has never received drinks other than breast milk, formula and water proceed to Question 5.*

5. The following questions relate to occasions when your baby consumed **solid foods** this week. Please indicate which of the follow foods your baby received and, on each occasion your baby was offered that food, the average amount he/she consumed with respect to a "spoon" or "mound" code as indicated in the Instructions.[C]

		Not at all	1-3 times per week	4-6 times per week	1-2 times per day	3-4 times per day	5 or more times per day	Average amount eaten per occasion
5.1	Vegetables including potato and baked beans	☐	☐	☐	☐	☐	☐	________
5.2	Fruit	☐	☐	☐	☐	☐	☐	________
5.3	Egg	☐	☐	☐	☐	☐	☐	________
5.4	Dairy products including butter, cheeses, cream, yoghurt, custard and ice cream	☐	☐	☐	☐	☐	☐	________
5.5	Wheat containing products including breads, biscuits, rusks, cakes, breakfast cereals, pastries, pasta, couscous and dim sims	☐	☐	☐	☐	☐	☐	________
5.6	Rice and/or rice containing products including rice cereals (e.g. Farax), rice cakes, rice noodles and rice flour	☐	☐	☐	☐	☐	☐	________
5.7	Soy containing products including tofu, miso, soy sauce, soy flour and soy-based dairy substitutes	☐	☐	☐	☐	☐	☐	________

[B] Refer to "Footnote B: Instructions for Question 4" on Page 3 for further clarification about answer this question
[C] Refer to "Footnote C: Instructions for Question 5" on Page 3 for further clarification about answer this question

		Not at all	1-3 times per week	4-6 times per week	1-2 times per day	3-4 times per day	5 or more times per day	Average amount eaten per occasion
5.8	Corn and/or corn containing products including popcorn, polenta, corn flakes, corn tortillas and corn flour (including products thickened with e.g. gravy)	☐	☐	☐	☐	☐	☐	________
5.9	Oats or oat containing products including porridge, oatmeal, muesli, ANZAC biscuits, bran and oat flour	☐	☐	☐	☐	☐	☐	________
5.10	Barley or rye containing products including barley water, many minestrone-style soups and rye bread	☐	☐	☐	☐	☐	☐	________
5.11	Nuts including peanut butter	☐	☐	☐	☐	☐	☐	________
5.12	Foods containing pork, lamb, chicken, turkey, or other poultry	☐	☐	☐	☐	☐	☐	________
5.13	Foods containing beef and veal	☐	☐	☐	☐	☐	☐	________
5.14	Foods containing fish or shellfish	☐	☐	☐	☐	☐	☐	________

If your baby consumed any of the solid foods outlined above, please provide some additional information about what those foods were with respect to each category. This may include the **name of the food**, the **brand of the product** (where applicable) and whether the food was **modified** (for example: low-fat, added calcium, wholemeal etc.).[D] Please also list any foods your baby consumed that do not fall in the above categories.

5.15

[D] Refer to "Footnote D: Instructions for Question 5" on Page 5 for further clarification about answer this question

What type of methods we used to cook your baby's solid foods (tick all that applies)?

5.16 What type of methods we used to cook your baby's solid foods (tick all that applies)?

☐ Did not cook ☐ Baking ☐ Steaming

☐ Grilling ☐ Roasting ☐ Boiling

☐ Stewing ☐ Frying ☐ BBQ

☐ Microwaving ☐ Other___________________________________

6. The following questions relates to the use of dummies/pacifiers.

6.1 Do you regularly offer your baby a dummy/pacifier?

☐ Yes ☐ No

6.2 Did you use any sweeteners on the dummy/pacifier?

☐ No ☐ Yes, honey

☐ Yes, other ____

7. The following questions relate to occasion when your baby was given **supplements and medications**.[E] Please state the brand, what the volume of a "dose" was (e.g. 5 mL, 1 tablet, etc.), how many doses were given each day and on how many days it was given **this week**.

		Dose volume	Doses per day	Days
7.1	Supplements			
	☐ Multivitamin (brand:____________________)	_____	_____	_____
	☐ Omega-3/fish oil (brand:_________________)	_____	_____	_____
	☐ Iron (brand:____________________________)	_____	_____	_____
	☐ Other:_________________________________	_____	_____	_____
7.2	Antibiotics			
	Name: ___________________________________	_____	_____	_____
	Name: ___________________________________	_____	_____	_____
	Name: ___________________________________	_____	_____	_____
7.3	Other as outlined in *Footnote E* (please specify)			
	Name: ___________________________________	_____	_____	_____
	Name: ___________________________________	_____	_____	_____
	Name: ___________________________________	_____	_____	_____

[E] Refer to "Footnote E: Instructions for Question 7" on Page 6 for further clarification about answer this question

8. Please describe your child's health this week, specifying any **fevers** and/or illnesses that required you to visit a healthcare profession (e.g. child health nurse, doctor, naturopath, etc.)

9. Did your child receive any vaccinations this week, either scheduled or optional? If you child was not given the vaccination as per the National Immunisation Program (NIP) guidelines (for example, one particular vaccine was not given but others were) please provide details.

	Description	Timing	As per NIP guidelines	Variation of NIP guidelines (give details)
9.1	Scheduled	Birth	☐	
		2 months	☐	
		4 months	☐	
		6 months	☐	
		12 months	☐	
9.2	Optional	Influenza	☐	
		Pneumococcal 13vPCV	☐	
		Pneumococcal 23vPPV	☐	
		Hepatitis A	☐	
		Other:__________________	☐	

10. Did you (the baby's mother) take any antibiotics just prior to your baby's birth, during the labour or since giving birth? Please provide details about the brand and dosage as described in Question 7.

		Yes	No	Unsure
10.1	In the week before giving birth	☐<br>Type: ____________________<br>Dose volume: _______________<br>Doses per day: ______________<br>Number of days: _____________	☐	☐

10.2	During labour	☐<br>Type: ____________________<br>Dose volume: ______________<br>Doses per day: _____________<br>Number of days: ____________	☐	☐
10.3	In the week since giving birth	☐<br>Type: ____________________<br>Dose volume: ______________<br>Doses per day: _____________<br>Number of days: ____________	☐	☐

Your baby is 2 weeks old

Completion date: ____/____/____

1. In the past 7 days, how often was your baby feed each item listed below on average? Consider everyone that has feed the baby. Include snacks and night-time feeds.[A]

		Number feeds per day	Number of days given	Not given
1.1	Breast milk via breast feeding	_____	_____	☐
1.2	Expressed breast milk	_____	_____	☐
1.3	Formula	_____	_____	☐
1.4	Water	_____	_____	☐
1.5	Sugar water	_____	_____	☐
1.6	Cow's milk*	_____	_____	☐
1.7	Other milk**	_____	_____	☐
1.8	Fruit juices	_____	_____	☐
1.9	Other drinks‡	_____	_____	☐

* Includes plain, low-fat, evaporated and flavoured varieties of cow's milk. Include milk added to other foods such as cereal.

** Such as soy, rice, almond or goat milk. Include milk added to other foods such as cereal.

‡ Includes vegetable juice, soft drinks, cordial, sports drinks, yoghurt drinks, tea, coffee, etc.

2. The following questions relate to occasions when your baby consumed **breast milk**.

☐ *My baby did not consume breast milk this week (proceed to Question 3).*

2.1 Average feed duration when being breast feed:

_____________minutes

2.2 Average volume *consumed* when provided expressed breast milk:

_____________mL

☐ Never provided expressed breast milk *(proceed to Question 2.5)*

2.3 How long was expressed breast milk stored before it was fed to your baby (tick all that applies)?

☐ Kept at room temperature ☐ 1 day or less in refrigerator
☐ 2-3 days in refrigerator ☐ 3-4 days in refrigerator
☐ 5-7 days in refrigerator ☐ Frozen (for any length of time)

2.4 On the majority of occasions, how were bottles containing expressed breast milk warmed (tick only one)?

☐ Did not warm ☐ Hot water[Ψ]\bottle warmer
☐ Microwave ☐ Other____________________________

[A] Refer to "Footnote A: Instructions for Question 1" on Page 2 for further clarification about answer this question

2.5	Did you (the baby's mother) take any antibiotics this week? Please provide details about the type of antibiotic and dosage as described in Question 7.<br>☐ No ☐ Unsure<br>☐ Yes Type: ______________________________<br>Dose volume: ________________________<br>Doses per day: _______________________<br>Number of days: ______________________

ᴪ When bottle containing the milk is placed into a container of hot water

3. The following questions relate to occasions when your baby consumed **formula**.

☐ *My baby did not consume formula this week (proceed to Question 4).*

3.1	Average volume consumed with provided formula:<br>_____________mL
3.2	What were the brand(s) and product name(s) of the formula(s) your baby been given. For example, "Wyeth S-26 GOLD Newborn" or "Karicare Aptamil® Delact"<br>1.____<br>2.____<br>3.____<br>☐ Unknown
3.3	Was the formula prepared according to the manufacturer's instructions?<br>☐ Yes<br>☐ No (please provide details of how the formula was prepared)<br>____<br>____<br>____
3.5	On the majority of occasions, how were bottles containing formula warmed (tick only one)?<br>☐ Did not warm ☐ Prepared with hot water<br>☐ Hot waterᴪ\bottle warmer ☐ Microwave<br>☐ Other_____________________________

ᴪ When bottle containing the milk is placed into a container of hot water

4. The following question relates to occasions when your baby consumed **drinks other than breast milk, formula or plain water**. This includes cow's milk or milk from other non-dairy sources as well as fruit juices, vegetable juices, cordials, soft drinks, etc. Please state what the drink(s) were, the **brand of the product** (where applicable) and how much your baby consumed on occasions

these drinks were provided. Drink additives should also be included.[B] *If your baby has never received drinks other than breast milk, formula and water proceed to question 5.*

5. The following questions relate to occasions when your baby consumed **solid foods** this week. Please indicate which of the follow foods your baby received and, on each occasion your baby was offered that food, the average amount he/she consumed with respect to a "spoon" or "mound" code as indicated in the Instructions.[C]

		Not at all	1-3 times per week	4-6 times per week	1-2 times per day	3-4 times per day	5 or more times per day	Average amount eaten per occasion
5.1	Vegetables including potato and baked beans	☐	☐	☐	☐	☐	☐	________
5.2	Fruit	☐	☐	☐	☐	☐	☐	________
5.3	Egg	☐	☐	☐	☐	☐	☐	________
5.4	Dairy products including butter, cheeses, cream, yoghurt, custard and ice cream	☐	☐	☐	☐	☐	☐	________
5.5	Wheat containing products including breads, biscuits, rusks, cakes, breakfast cereals, pastries, pasta, couscous and dim sims	☐	☐	☐	☐	☐	☐	________
5.6	Rice and/or rice containing products including rice cereals (e.g. Farax), rice cakes, rice noodles and rice flour	☐	☐	☐	☐	☐	☐	________
5.7	Soy containing products including tofu, miso, soy sauce, soy flour and soy-based dairy substitutes	☐	☐	☐	☐	☐	☐	________
5.8	Corn and/or corn containing products including popcorn, polenta, corn flakes, corn tortillas and corn flour (including products thickened with e.g. gravy)	☐	☐	☐	☐	☐	☐	________

[B] Refer to "Footnote B: Instructions for Question 4" on Page 3 for further clarification about answer this question

[C] Refer to "Footnote C: Instructions for Question 5" on Page 3 for further clarification about answer this question

		Not at all	1-3 times per week	4-6 times per week	1-2 times per day	3-4 times per day	5 or more times per day	Average amount eaten per occasion
5.9	Oats or oat containing products including porridge, oatmeal, muesli, ANZAC biscuits, bran and oat flour	☐	☐	☐	☐	☐	☐	________
5.10	Barley or rye containing products including barley water, many minestrone-style soups and rye bread	☐	☐	☐	☐	☐	☐	________
5.11	Nuts including peanut butter	☐	☐	☐	☐	☐	☐	________
5.12	Foods containing pork, lamb, chicken, turkey, or other poultry	☐	☐	☐	☐	☐	☐	________
5.13	Foods containing beef and veal	☐	☐	☐	☐	☐	☐	________
5.14	Foods containing fish or shellfish	☐	☐	☐	☐	☐	☐	________

If your baby consumed any of the solid foods outlined above, please provide some additional information about what those foods were with respect to each category. This may include the **name of the food**, the **brand of the product** (where applicable) and whether the food was **modified** (for example: low-fat, added calcium, wholemeal etc.).[D] Please also list any foods your baby consumed that do not fall in the above categories.

5.15

[D] Refer to "Footnote D: Instructions for Question 5" on Page 5 for further clarification about answer this question

What type of methods we used to cook your baby's solid foods (tick all that applies)?

5.16	What type of methods we used to cook your baby's solid foods (tick all that applies)?<br>☐ Did not cook ☐ Baking ☐ Steaming<br>☐ Grilling ☐ Roasting ☐ Boiling<br>☐ Stewing ☐ Frying ☐ BBQ<br>☐ Microwaving ☐ Other___________________________________

6. The following questions relates to the use of dummies/pacifiers.

6.1	Do you regularly offer your baby a dummy/pacifier?<br>☐ Yes ☐ No
6.2	Did you use any sweeteners on the dummy/pacifier?<br>☐ No ☐ Yes, honey<br>☐ Yes, other ____

7. The following questions relate to occasion when your baby was given **supplements and medications**.[E] Please state the brand, what the volume of a "dose" was (e.g. 5 mL, 1 tablet, etc.), how many doses were given each day and on how many days it was given **this week**.

		Dose volume	Doses per day	Days
7.1	Supplements			
	☐ Multivitamin (brand:____________________)	_____	_____	_____
	☐ Omega-3/fish oil (brand:_________________)	_____	_____	_____
	☐ Iron (brand:____________________________)	_____	_____	_____
	☐ Other:_________________________________	_____	_____	_____
7.2	Antibiotics			
	Name:____________________________________	_____	_____	_____
	Name:____________________________________	_____	_____	_____
	Name:____________________________________	_____	_____	_____
7.3	Other as outlined in *Footnote E* (please specify)			
	Name:____________________________________	_____	_____	_____
	Name:____________________________________	_____	_____	_____
	Name:____________________________________	_____	_____	_____

[E] Refer to "Footnote E: Instructions for Question 7" on Page 6 for further clarification about answer this question

8. Please describe your child's health this week, specifying any **fevers** and/or illnesses that required you to visit a healthcare profession (e.g. child health nurse, doctor, naturopath, etc.)

9. Did your child receive any vaccinations this week, either scheduled or optional? If you child was not given the vaccination as per the National Immunisation Program (NIP) guidelines (for example, one particular vaccine was not given but others were) please provide details.

	Description	Timing	As per NIP guidelines	Variation of NIP guidelines (give details)
9.1	Scheduled	Birth	☐	
		2 months	☐	
		4 months	☐	
		6 months	☐	
		12 months	☐	
9.2	Optional	Influenza	☐	
		Pneumococcal 13vPCV	☐	
		Pneumococcal 23vPPV	☐	
		Hepatitis A	☐	
		Other:_________________	☐	

Your baby is 3 weeks old

Completion date: ____/____/____

1. In the past 7 days, how often was your baby feed each item listed below on average? Consider everyone that has feed the baby. Include snacks and night-time feeds.[A]

		Number feeds per day	Number of days given	Not given
1.1	Breast milk via breast feeding	_____	_____	☐
1.2	Expressed breast milk	_____	_____	☐
1.3	Formula	_____	_____	☐
1.4	Water	_____	_____	☐
1.5	Sugar water	_____	_____	☐
1.6	Cow's milk*	_____	_____	☐
1.7	Other milk**	_____	_____	☐
1.8	Fruit juices	_____	_____	☐
1.9	Other drinks‡	_____	_____	☐

* Includes plain, low-fat, evaporated and flavoured varieties of cow's milk. Include milk added to other foods such as cereal.

** Such as soy, rice, almond or goat milk. Include milk added to other foods such as cereal.

‡ Includes vegetable juice, soft drinks, cordial, sports drinks, yoghurt drinks, tea, coffee, etc.

2. The following questions relate to occasions when your baby consumed **breast milk**.

☐ *My baby did not consume breast milk this week (proceed to Question 3).*

2.1 Average feed duration when being breast feed:

______________minutes

2.2 Average volume *consumed* when provided expressed breast milk:

______________mL

☐ Never provided expressed breast milk *(proceed to Question 2.5)*

2.3 How long was expressed breast milk stored before it was fed to your baby (tick all that applies)?

☐ Kept at room temperature ☐ 1 day or less in refrigerator
☐ 2-3 days in refrigerator ☐ 3-4 days in refrigerator
☐ 5-7 days in refrigerator ☐ Frozen (for any length of time)

2.4 On the majority of occasions, how were bottles containing expressed breast milk warmed (tick only one)?

☐ Did not warm ☐ Hot water[Ψ]\bottle warmer
☐ Microwave ☐ Other______________________________

[A] Refer to "Footnote A: Instructions for Question 1" on Page 2 for further clarification about answer this question

2.5	Did you (the baby's mother) take any antibiotics this week? Please provide details about the type of antibiotic and dosage as described in Question 7.<br>☐ No ☐ Unsure<br>☐ Yes Type: ______________________________<br>Dose volume: ________________________<br>Doses per day: _______________________<br>Number of days: ______________________

Ψ When bottle containing the milk is placed into a container of hot water

3. The following questions relate to occasions when your baby consumed **formula**.

   ☐ *My baby did not consume formula this week (proceed to Question 4).*

3.1	Average volume consumed with provided formula:<br>_____________mL
3.2	What were the brand(s) and product name(s) of the formula(s) your baby been given. For example, "Wyeth S-26 GOLD Newborn" or "Karicare Aptamil® Delact"<br>1.____<br>2.____<br>3.____<br>☐ Unknown
3.3	Was the formula prepared according to the manufacturer's instructions?<br>☐ Yes<br>☐ No (please provide details of how the formula was prepared)<br>____<br>____<br>____
3.5	On the majority of occasions, how were bottles containing formula warmed (tick only one)?<br>☐ Did not warm ☐ Prepared with hot water<br>☐ Hot water$^{\Psi}$\bottle warmer ☐ Microwave<br>☐ Other____________________________

Ψ When bottle containing the milk is placed into a container of hot water

4. The following question relates to occasions when your baby consumed **drinks other than breast milk, formula or plain water**. This includes cow's milk or milk from other non-dairy sources as well as fruit juices, vegetable juices, cordials, soft drinks, etc. Please state what the drink(s) were, the **brand of the product** (where applicable) and how much your baby consumed on occasions

these drinks were provided. Drink additives should also be included.[B] *If your baby has never received drinks other than breast milk, formula and water proceed to question 5.*

5. The following questions relate to occasions when your baby consumed **solid foods** this week. Please indicate which of the follow foods your baby received and, on each occasion your baby was offered that food, the average amount he/she consumed with respect to a "spoon" or "mound" code as indicated in the Instructions.[C]

		Not at all	1-3 times per week	4-6 times per week	1-2 times per day	3-4 times per day	5 or more times per day	Average amount eaten per occasion
5.1	Vegetables including potato and baked beans	☐	☐	☐	☐	☐	☐	________
5.2	Fruit	☐	☐	☐	☐	☐	☐	________
5.3	Egg	☐	☐	☐	☐	☐	☐	________
5.4	Dairy products including butter, cheeses, cream, yoghurt, custard and ice cream	☐	☐	☐	☐	☐	☐	________
5.5	Wheat containing products including breads, biscuits, rusks, cakes, breakfast cereals, pastries, pasta, couscous and dim sims	☐	☐	☐	☐	☐	☐	________
5.6	Rice and/or rice containing products including rice cereals (e.g. Farax), rice cakes, rice noodles and rice flour	☐	☐	☐	☐	☐	☐	________
5.7	Soy containing products including tofu, miso, soy sauce, soy flour and soy-based dairy substitutes	☐	☐	☐	☐	☐	☐	________
5.8	Corn and/or corn containing products including popcorn, polenta, corn flakes, corn tortillas and corn flour (including products thickened with e.g. gravy)	☐	☐	☐	☐	☐	☐	________

[B] Refer to "Footnote B: Instructions for Question 4" on Page 3 for further clarification about answer this question
[C] Refer to "Footnote C: Instructions for Question 5" on Page 3 for further clarification about answer this question

		Not at all	1-3 times per week	4-6 times per week	1-2 times per day	3-4 times per day	5 or more times per day	Average amount eaten per occasion
5.9	Oats or oat containing products including porridge, oatmeal, muesli, ANZAC biscuits, bran and oat flour	☐	☐	☐	☐	☐	☐	________
5.10	Barley or rye containing products including barley water, many minestrone-style soups and rye bread	☐	☐	☐	☐	☐	☐	________
5.11	Nuts including peanut butter	☐	☐	☐	☐	☐	☐	________
5.12	Foods containing pork, lamb, chicken, turkey, or other poultry	☐	☐	☐	☐	☐	☐	________
5.13	Foods containing beef and veal	☐	☐	☐	☐	☐	☐	________
5.14	Foods containing fish or shellfish	☐	☐	☐	☐	☐	☐	________

If your baby consumed any of the solid foods outlined above, please provide some additional information about what those foods were with respect to each category. This may include the **name of the food**, the **brand of the product** (where applicable) and whether the food was **modified** (for example: low-fat, added calcium, wholemeal etc.).[D] Please also list any foods your baby consumed that do not fall in the above categories.

5.15

[D] Refer to "Footnote D: Instructions for Question 5" on Page 5 for further clarification about answer this question

What type of methods we used to cook your baby's solid foods (tick all that applies)?

5.16	What type of methods we used to cook your baby's solid foods (tick all that applies)?<br>☐ Did not cook ☐ Baking ☐ Steaming<br>☐ Grilling ☐ Roasting ☐ Boiling<br>☐ Stewing ☐ Frying ☐ BBQ<br>☐ Microwaving ☐ Other___________________________________

6. The following questions relates to the use of dummies/pacifiers.

6.1	Do you regularly offer your baby a dummy/pacifier?<br>☐ Yes ☐ No
6.2	Did you use any sweeteners on the dummy/pacifier?<br>☐ No ☐ Yes, honey<br>☐ Yes, other ____

7. The following questions relate to occasion when your baby was given **supplements and medications**.[E] Please state the brand, what the volume of a "dose" was (e.g. 5 mL, 1 tablet, etc.), how many doses were given each day and on how many days it was given **this week**.

		Dose volume	Doses per day	Days
7.1	Supplements			
	☐ Multivitamin (brand:____________________)	_____	_____	_____
	☐ Omega-3/fish oil (brand:_________________)	_____	_____	_____
	☐ Iron (brand:____________________________)	_____	_____	_____
	☐ Other:_________________________________	_____	_____	_____
7.2	Antibiotics			
	Name:____________________________________	_____	_____	_____
	Name:____________________________________	_____	_____	_____
	Name:____________________________________	_____	_____	_____
7.3	Other as outlined in *Footnote E* (please specify)			
	Name:____________________________________	_____	_____	_____
	Name:____________________________________	_____	_____	_____
	Name:____________________________________	_____	_____	_____

[E] Refer to "Footnote E: Instructions for Question 7" on Page 6 for further clarification about answer this question

8. Please describe your child's health this week, specifying any **fevers** and/or illnesses that required you to visit a healthcare profession (e.g. child health nurse, doctor, naturopath, etc.)

9. Did your child receive any vaccinations this week, either scheduled or optional? If you child was not given the vaccination as per the National Immunisation Program (NIP) guidelines (for example, one particular vaccine was not given but others were) please provide details.

	Description	Timing	As per NIP guidelines	Variation of NIP guidelines (give details)
9.1	Scheduled	Birth	☐	
		2 months	☐	
		4 months	☐	
		6 months	☐	
		12 months	☐	
9.2	Optional	Influenza	☐	
		Pneumococcal 13vPCV	☐	
		Pneumococcal 23vPPV	☐	
		Hepatitis A	☐	
		Other:__________________	☐	

Your baby is 4 weeks old

Completion date: ____/____/____

1. In the past 7 days, how often was your baby feed each item listed below on average? Consider everyone that has feed the baby. Include snacks and night-time feeds.[A]

		Number feeds per day	Number of days given	Not given
1.1	Breast milk via breast feeding	_____	_____	☐
1.2	Expressed breast milk	_____	_____	☐
1.3	Formula	_____	_____	☐
1.4	Water	_____	_____	☐
1.5	Sugar water	_____	_____	☐
1.6	Cow's milk*	_____	_____	☐
1.7	Other milk**	_____	_____	☐
1.8	Fruit juices	_____	_____	☐
1.9	Other drinks‡	_____	_____	☐

* Includes plain, low-fat, evaporated and flavoured varieties of cow's milk. Include milk added to other foods such as cereal.

** Such as soy, rice, almond or goat milk. Include milk added to other foods such as cereal.

‡ Includes vegetable juice, soft drinks, cordial, sports drinks, yoghurt drinks, tea, coffee, etc.

2. The following questions relate to occasions when your baby consumed **breast milk**.

☐ *My baby did not consume breast milk this week (proceed to Question 3).*

2.1 Average feed duration when being breast feed:

_____________minutes

2.2 Average volume *consumed* when provided expressed breast milk:

_____________mL

☐ Never provided expressed breast milk *(proceed to Question 2.5)*

2.3 How long was expressed breast milk stored before it was fed to your baby (tick all that applies)?

☐ Kept at room temperature ☐ 1 day or less in refrigerator
☐ 2-3 days in refrigerator ☐ 3-4 days in refrigerator
☐ 5-7 days in refrigerator ☐ Frozen (for any length of time)

2.4 On the majority of occasions, how were bottles containing expressed breast milk warmed (tick only one)?

☐ Did not warm ☐ Hot water[Ψ]\bottle warmer
☐ Microwave ☐ Other_____________________________

[A] Refer to "Footnote A: Instructions for Question 1" on Page 2 for further clarification about answer this question

2.5	Did you (the baby's mother) take any antibiotics this week? Please provide details about the type of antibiotic and dosage as described in Question 7.<br>☐ No ☐ Unsure<br>☐ Yes Type: ______________________________<br>Dose volume: ________________________<br>Doses per day: ______________________<br>Number of days: _____________________	

Ψ When bottle containing the milk is placed into a container of hot water

3. The following questions relate to occasions when your baby consumed **formula**.

   ☐ *My baby did not consume formula this week (proceed to Question 4).*

3.1	Average volume consumed with provided formula:<br>_____________mL
3.2	What were the brand(s) and product name(s) of the formula(s) your baby been given. For example, "Wyeth S-26 GOLD Newborn" or "Karicare Aptamil® Delact"<br>1.____<br>2.____<br>3.____<br>☐ Unknown
3.3	Was the formula prepared according to the manufacturer's instructions?<br>☐ Yes<br>☐ No (please provide details of how the formula was prepared)<br>____<br>____<br>____
3.5	On the majority of occasions, how were bottles containing formula warmed (tick only one)?<br>☐ Did not warm ☐ Prepared with hot water<br>☐ Hot waterΨ\bottle warmer ☐ Microwave<br>☐ Other____________________________

Ψ When bottle containing the milk is placed into a container of hot water

4. The following question relates to occasions when your baby consumed **drinks other than breast milk, formula or plain water**. This includes cow's milk or milk from other non-dairy sources as well as fruit juices, vegetable juices, cordials, soft drinks, etc. Please state what the drink(s) were, the **brand of the product** (where applicable) and how much your baby consumed on occasions

these drinks were provided. Drink additives should also be included.[B] *If your baby has never received drinks other than breast milk, formula and water proceed to question 5.*

5. The following questions relate to occasions when your baby consumed **solid foods** this week. Please indicate which of the follow foods your baby received and, on each occasion your baby was offered that food, the average amount he/she consumed with respect to a "spoon" or "mound" code as indicated in the Instructions.[C]

		Not at all	1-3 times per week	4-6 times per week	1-2 times per day	3-4 times per day	5 or more times per day	Average amount eaten per occasion
5.1	Vegetables including potato and baked beans	☐	☐	☐	☐	☐	☐	________
5.2	Fruit	☐	☐	☐	☐	☐	☐	________
5.3	Egg	☐	☐	☐	☐	☐	☐	________
5.4	Dairy products including butter, cheeses, cream, yoghurt, custard and ice cream	☐	☐	☐	☐	☐	☐	________
5.5	Wheat containing products including breads, biscuits, rusks, cakes, breakfast cereals, pastries, pasta, couscous and dim sims	☐	☐	☐	☐	☐	☐	________
5.6	Rice and/or rice containing products including rice cereals (e.g. Farax), rice cakes, rice noodles and rice flour	☐	☐	☐	☐	☐	☐	________
5.7	Soy containing products including tofu, miso, soy sauce, soy flour and soy-based dairy substitutes	☐	☐	☐	☐	☐	☐	________
5.8	Corn and/or corn containing products including popcorn, polenta, corn flakes, corn tortillas and corn flour (including products thickened with e.g. gravy)	☐	☐	☐	☐	☐	☐	________

[B] Refer to "Footnote B: Instructions for Question 4" on Page 3 for further clarification about answer this question
[C] Refer to "Footnote C: Instructions for Question 5" on Page 3 for further clarification about answer this question

		Not at all	1-3 times per week	4-6 times per week	1-2 times per day	3-4 times per day	5 or more times per day	Average amount eaten per occasion
5.9	Oats or oat containing products including porridge, oatmeal, muesli, ANZAC biscuits, bran and oat flour	☐	☐	☐	☐	☐	☐	________
5.10	Barley or rye containing products including barley water, many minestrone-style soups and rye bread	☐	☐	☐	☐	☐	☐	________
5.11	Nuts including peanut butter	☐	☐	☐	☐	☐	☐	________
5.12	Foods containing pork, lamb, chicken, turkey, or other poultry	☐	☐	☐	☐	☐	☐	________
5.13	Foods containing beef and veal	☐	☐	☐	☐	☐	☐	________
5.14	Foods containing fish or shellfish	☐	☐	☐	☐	☐	☐	________

If your baby consumed any of the solid foods outlined above, please provide some additional information about what those foods were with respect to each category. This may include the **name of the food**, the **brand of the product** (where applicable) and whether the food was **modified** (for example: low-fat, added calcium, wholemeal etc.).[D] Please also list any foods your baby consumed that do not fall in the above categories.

5.15

[D] Refer to "Footnote D: Instructions for Question 5" on Page 5 for further clarification about answer this question

What type of methods we used to cook your baby's solid foods (tick all that applies)?

5.16	What type of methods we used to cook your baby's solid foods (tick all that applies)?<br>☐ Did not cook ☐ Baking ☐ Steaming<br>☐ Grilling ☐ Roasting ☐ Boiling<br>☐ Stewing ☐ Frying ☐ BBQ<br>☐ Microwaving ☐ Other___________________________________

6. The following questions relates to the use of dummies/pacifiers.

6.1	Do you regularly offer your baby a dummy/pacifier?<br>☐ Yes ☐ No
6.2	Did you use any sweeteners on the dummy/pacifier?<br>☐ No ☐ Yes, honey<br>☐ Yes, other ____

7. The following questions relate to occasion when your baby was given **supplements and medications**.[E] Please state the brand, what the volume of a "dose" was (e.g. 5 mL, 1 tablet, etc.), how many doses were given each day and on how many days it was given **this week**.

		Dose volume	Doses per day	Days
7.1	Supplements			
	☐ Multivitamin (brand:____________________)	_____	_____	_____
	☐ Omega-3/fish oil (brand:________________)	_____	_____	_____
	☐ Iron (brand:___________________________)	_____	_____	_____
	☐ Other:_________________________________	_____	_____	_____
7.2	Antibiotics			
	Name:____________________________________	_____	_____	_____
	Name:____________________________________	_____	_____	_____
	Name:____________________________________	_____	_____	_____
7.3	Other as outlined in *Footnote E* (please specify)			
	Name:____________________________________	_____	_____	_____
	Name:____________________________________	_____	_____	_____
	Name:____________________________________	_____	_____	_____

[E] Refer to "Footnote E: Instructions for Question 7" on Page 6 for further clarification about answer this question

8. Please describe your child's health this week, specifying any **fevers** and/or illnesses that required you to visit a healthcare profession (e.g. child health nurse, doctor, naturopath, etc.)

9. Did your child receive any vaccinations this week, either scheduled or optional? If you child was not given the vaccination as per the National Immunisation Program (NIP) guidelines (for example, one particular vaccine was not given but others were) please provide details.

	Description	Timing	As per NIP guidelines	Variation of NIP guidelines (give details)
9.1	Scheduled	Birth	☐	
		2 months	☐	
		4 months	☐	
		6 months	☐	
		12 months	☐	
9.2	Optional	Influenza	☐	
		Pneumococcal 13vPCV	☐	
		Pneumococcal 23vPPV	☐	
		Hepatitis A	☐	
		Other:__________________	☐	

Your baby is 5 weeks old

Completion date: ____/____/____

1. In the past 7 days, how often was your baby feed each item listed below on average? Consider everyone that has feed the baby. Include snacks and night-time feeds.[A]

		Number feeds per day	Number of days given	Not given
1.1	Breast milk via breast feeding	_____	_____	☐
1.2	Expressed breast milk	_____	_____	☐
1.3	Formula	_____	_____	☐
1.4	Water	_____	_____	☐
1.5	Sugar water	_____	_____	☐
1.6	Cow's milk*	_____	_____	☐
1.7	Other milk**	_____	_____	☐
1.8	Fruit juices	_____	_____	☐
1.9	Other drinks‡	_____	_____	☐

* Includes plain, low-fat, evaporated and flavoured varieties of cow's milk. Include milk added to other foods such as cereal.

** Such as soy, rice, almond or goat milk. Include milk added to other foods such as cereal.

‡ Includes vegetable juice, soft drinks, cordial, sports drinks, yoghurt drinks, tea, coffee, etc.

2. The following questions relate to occasions when your baby consumed **breast milk**.

☐ *My baby did not consume breast milk this week (proceed to Question 3).*

2.1 Average feed duration when being breast feed:

_____________minutes

2.2 Average volume *consumed* when provided expressed breast milk:

_____________mL

☐ Never provided expressed breast milk *(proceed to Question 2.5)*

2.3 How long was expressed breast milk stored before it was fed to your baby (tick all that applies)?

☐ Kept at room temperature ☐ 1 day or less in refrigerator
☐ 2-3 days in refrigerator ☐ 3-4 days in refrigerator
☐ 5-7 days in refrigerator ☐ Frozen (for any length of time)

2.4 On the majority of occasions, how were bottles containing expressed breast milk warmed (tick only one)?

☐ Did not warm ☐ Hot water[Ψ]\bottle warmer
☐ Microwave ☐ Other____________________________

[A] Refer to "Footnote A: Instructions for Question 1" on Page 2 for further clarification about answer this question

2.5	Did you (the baby's mother) take any antibiotics this week? Please provide details about the type of antibiotic and dosage as described in Question 7.<br>☐ No ☐ Unsure<br>☐ Yes Type: ______________________________<br>Dose volume: ________________________<br>Doses per day: _______________________<br>Number of days: ______________________

ψ When bottle containing the milk is placed into a container of hot water

3. The following questions relate to occasions when your baby consumed **formula**.

   ☐ *My baby did not consume formula this week (proceed to Question 4).*

3.1	Average volume consumed with provided formula:<br>_____________mL
3.2	What were the brand(s) and product name(s) of the formula(s) your baby been given. For example, "Wyeth S-26 GOLD Newborn" or "Karicare Aptamil® Delact"<br>1.____<br>2.____<br>3.____<br>☐ Unknown
3.3	Was the formula prepared according to the manufacturer's instructions?<br>☐ Yes<br>☐ No (please provide details of how the formula was prepared)<br>____<br>____<br>____
3.5	On the majority of occasions, how were bottles containing formula warmed (tick only one)?<br>☐ Did not warm ☐ Prepared with hot water<br>☐ Hot waterψ\bottle warmer ☐ Microwave<br>☐ Other_____________________________

ψ When bottle containing the milk is placed into a container of hot water

4. The following question relates to occasions when your baby consumed **drinks other than breast milk, formula or plain water**. This includes cow's milk or milk from other non-dairy sources as well as fruit juices, vegetable juices, cordials, soft drinks, etc. Please state what the drink(s) were, the **brand of the product** (where applicable) and how much your baby consumed on occasions

these drinks were provided. Drink additives should also be included.[B] *If your baby has never received drinks other than breast milk, formula and water proceed to question 5.*

5. The following questions relate to occasions when your baby consumed **solid foods** this week. Please indicate which of the follow foods your baby received and, on each occasion your baby was offered that food, the average amount he/she consumed with respect to a "spoon" or "mound" code as indicated in the Instructions.[C]

		Not at all	1-3 times per week	4-6 times per week	1-2 times per day	3-4 times per day	5 or more times per day	Average amount eaten per occasion
5.1	Vegetables including potato and baked beans	☐	☐	☐	☐	☐	☐	________
5.2	Fruit	☐	☐	☐	☐	☐	☐	________
5.3	Egg	☐	☐	☐	☐	☐	☐	________
5.4	Dairy products including butter, cheeses, cream, yoghurt, custard and ice cream	☐	☐	☐	☐	☐	☐	________
5.5	Wheat containing products including breads, biscuits, rusks, cakes, breakfast cereals, pastries, pasta, couscous and dim sims	☐	☐	☐	☐	☐	☐	________
5.6	Rice and/or rice containing products including rice cereals (e.g. Farax), rice cakes, rice noodles and rice flour	☐	☐	☐	☐	☐	☐	________
5.7	Soy containing products including tofu, miso, soy sauce, soy flour and soy-based dairy substitutes	☐	☐	☐	☐	☐	☐	________
5.8	Corn and/or corn containing products including popcorn, polenta, corn flakes, corn tortillas and corn flour (including products thickened with e.g. gravy)	☐	☐	☐	☐	☐	☐	________

[B] Refer to "Footnote B: Instructions for Question 4" on Page 3 for further clarification about answer this question
[C] Refer to "Footnote C: Instructions for Question 5" on Page 3 for further clarification about answer this question

		Not at all	1-3 times per week	4-6 times per week	1-2 times per day	3-4 times per day	5 or more times per day	Average amount eaten per occasion
5.9	Oats or oat containing products including porridge, oatmeal, muesli, ANZAC biscuits, bran and oat flour	☐	☐	☐	☐	☐	☐	________
5.10	Barley or rye containing products including barley water, many minestrone-style soups and rye bread	☐	☐	☐	☐	☐	☐	________
5.11	Nuts including peanut butter	☐	☐	☐	☐	☐	☐	________
5.12	Foods containing pork, lamb, chicken, turkey, or other poultry	☐	☐	☐	☐	☐	☐	________
5.13	Foods containing beef and veal	☐	☐	☐	☐	☐	☐	________
5.14	Foods containing fish or shellfish	☐	☐	☐	☐	☐	☐	________

If your baby consumed any of the solid foods outlined above, please provide some additional information about what those foods were with respect to each category. This may include the **name of the food**, the **brand of the product** (where applicable) and whether the food was **modified** (for example: low-fat, added calcium, wholemeal etc.).[D] Please also list any foods your baby consumed that do not fall in the above categories.

5.15

[D] Refer to "Footnote D: Instructions for Question 5" on Page 5 for further clarification about answer this question

What type of methods we used to cook your baby's solid foods (tick all that applies)?

5.16	What type of methods we used to cook your baby's solid foods (tick all that applies)?			
	☐ Did not cook	☐ Baking	☐ Steaming	
	☐ Grilling	☐ Roasting	☐ Boiling	
	☐ Stewing	☐ Frying	☐ BBQ	
	☐ Microwaving	☐ Other___________________________________		

6. The following questions relates to the use of dummies/pacifiers.

6.1	Do you regularly offer your baby a dummy/pacifier?		
	☐ Yes	☐ No	
6.2	Did you use any sweeteners on the dummy/pacifier?		
	☐ No	☐ Yes, honey	
	☐ Yes, other ____		

7. The following questions relate to occasion when your baby was given **supplements and medications**.[E] Please state the brand, what the volume of a "dose" was (e.g. 5 mL, 1 tablet, etc.), how many doses were given each day and on how many days it was given **this week**.

		Dose volume	Doses per day	Days
7.1	Supplements			
	☐ Multivitamin (brand:____________________)	_____	_____	_____
	☐ Omega-3/fish oil (brand:_________________)	_____	_____	_____
	☐ Iron (brand:____________________________)	_____	_____	_____
	☐ Other:_________________________________	_____	_____	_____
7.2	Antibiotics			
	Name:____________________________________	_____	_____	_____
	Name:____________________________________	_____	_____	_____
	Name:____________________________________	_____	_____	_____
7.3	Other as outlined in *Footnote E* (please specify)			
	Name:____________________________________	_____	_____	_____
	Name:____________________________________	_____	_____	_____
	Name:____________________________________	_____	_____	_____

[E] Refer to "Footnote E: Instructions for Question 7" on Page 6 for further clarification about answer this question

8. Please describe your child's health this week, specifying any **fevers** and/or illnesses that required you to visit a healthcare profession (e.g. child health nurse, doctor, naturopath, etc.)

9. Did your child receive any vaccinations this week, either scheduled or optional? If you child was not given the vaccination as per the National Immunisation Program (NIP) guidelines (for example, one particular vaccine was not given but others were) please provide details.

	Description	Timing	As per NIP guidelines	Variation of NIP guidelines (give details)
9.1	Scheduled	Birth	☐	
		2 months	☐	
		4 months	☐	
		6 months	☐	
		12 months	☐	
9.2	Optional	Influenza	☐	
		Pneumococcal 13vPCV	☐	
		Pneumococcal 23vPPV	☐	
		Hepatitis A	☐	
		Other:_________________	☐	

Your baby is 6 weeks old

Completion date: ____/____/____

1. In the past 7 days, how often was your baby feed each item listed below on average? Consider everyone that has feed the baby. Include snacks and night-time feeds.[A]

		Number feeds per day	Number of days given	Not given
1.1	Breast milk via breast feeding	_____	_____	☐
1.2	Expressed breast milk	_____	_____	☐
1.3	Formula	_____	_____	☐
1.4	Water	_____	_____	☐
1.5	Sugar water	_____	_____	☐
1.6	Cow's milk*	_____	_____	☐
1.7	Other milk**	_____	_____	☐
1.8	Fruit juices	_____	_____	☐
1.9	Other drinks‡	_____	_____	☐

* Includes plain, low-fat, evaporated and flavoured varieties of cow's milk. Include milk added to other foods such as cereal.

** Such as soy, rice, almond or goat milk. Include milk added to other foods such as cereal.

‡ Includes vegetable juice, soft drinks, cordial, sports drinks, yoghurt drinks, tea, coffee, etc.

2. The following questions relate to occasions when your baby consumed **breast milk**.

☐ *My baby did not consume breast milk this week (proceed to Question 3).*

2.1 Average feed duration when being breast feed:

______________minutes

2.2 Average volume *consumed* when provided expressed breast milk:

______________mL

☐ Never provided expressed breast milk *(proceed to Question 2.5)*

2.3 How long was expressed breast milk stored before it was fed to your baby (tick all that applies)?

☐ Kept at room temperature ☐ 1 day or less in refrigerator
☐ 2-3 days in refrigerator ☐ 3-4 days in refrigerator
☐ 5-7 days in refrigerator ☐ Frozen (for any length of time)

2.4 On the majority of occasions, how were bottles containing expressed breast milk warmed (tick only one)?

☐ Did not warm ☐ Hot water[Ψ]\bottle warmer
☐ Microwave ☐ Other______________________________

[A] Refer to "Footnote A: Instructions for Question 1" on Page 2 for further clarification about answer this question

2.5	Did you (the baby's mother) take any antibiotics this week? Please provide details about the type of antibiotic and dosage as described in Question 7.<br>☐ No ☐ Unsure<br>☐ Yes Type: ______________________________<br>Dose volume: _______________________<br>Doses per day: ______________________<br>Number of days: _____________________

ᴪ When bottle containing the milk is placed into a container of hot water

3. The following questions relate to occasions when your baby consumed **formula**.

   ☐ *My baby did not consume formula this week (proceed to Question 4).*

3.1	Average volume consumed with provided formula:<br>____________mL
3.2	What were the brand(s) and product name(s) of the formula(s) your baby been given. For example, "Wyeth S-26 GOLD Newborn" or "Karicare Aptamil® Delact"<br>1.____<br>2.____<br>3.____<br>☐ Unknown
3.3	Was the formula prepared according to the manufacturer's instructions?<br>☐ Yes<br>☐ No (please provide details of how the formula was prepared)<br>____<br>____<br>____
3.5	On the majority of occasions, how were bottles containing formula warmed (tick only one)?<br>☐ Did not warm ☐ Prepared with hot water<br>☐ Hot waterᴪ\bottle warmer ☐ Microwave<br>☐ Other___________________________

ᴪ When bottle containing the milk is placed into a container of hot water

4. The following question relates to occasions when your baby consumed **drinks other than breast milk, formula or plain water**. This includes cow's milk or milk from other non-dairy sources as well as fruit juices, vegetable juices, cordials, soft drinks, etc. Please state what the drink(s) were, the **brand of the product** (where applicable) and how much your baby consumed on occasions

these drinks were provided. Drink additives should also be included.[B] *If your baby has never received drinks other than breast milk, formula and water proceed to question 5.*

5. The following questions relate to occasions when your baby consumed **solid foods** this week. Please indicate which of the follow foods your baby received and, on each occasion your baby was offered that food, the average amount he/she consumed with respect to a "spoon" or "mound" code as indicated in the Instructions.[C]

		Not at all	1-3 times per week	4-6 times per week	1-2 times per day	3-4 times per day	5 or more times per day	Average amount eaten per occasion
5.1	Vegetables including potato and baked beans	☐	☐	☐	☐	☐	☐	________
5.2	Fruit	☐	☐	☐	☐	☐	☐	________
5.3	Egg	☐	☐	☐	☐	☐	☐	________
5.4	Dairy products including butter, cheeses, cream, yoghurt, custard and ice cream	☐	☐	☐	☐	☐	☐	________
5.5	Wheat containing products including breads, biscuits, rusks, cakes, breakfast cereals, pastries, pasta, couscous and dim sims	☐	☐	☐	☐	☐	☐	________
5.6	Rice and/or rice containing products including rice cereals (e.g. Farax), rice cakes, rice noodles and rice flour	☐	☐	☐	☐	☐	☐	________
5.7	Soy containing products including tofu, miso, soy sauce, soy flour and soy-based dairy substitutes	☐	☐	☐	☐	☐	☐	________
5.8	Corn and/or corn containing products including popcorn, polenta, corn flakes, corn tortillas and corn flour (including products thickened with e.g. gravy)	☐	☐	☐	☐	☐	☐	________

[B] Refer to "Footnote B: Instructions for Question 4" on Page 3 for further clarification about answer this question
[C] Refer to "Footnote C: Instructions for Question 5" on Page 3 for further clarification about answer this question

		Not at all	1-3 times per week	4-6 times per week	1-2 times per day	3-4 times per day	5 or more times per day	Average amount eaten per occasion
5.9	Oats or oat containing products including porridge, oatmeal, muesli, ANZAC biscuits, bran and oat flour	☐	☐	☐	☐	☐	☐	________
5.10	Barley or rye containing products including barley water, many minestrone-style soups and rye bread	☐	☐	☐	☐	☐	☐	________
5.11	Nuts including peanut butter	☐	☐	☐	☐	☐	☐	________
5.12	Foods containing pork, lamb, chicken, turkey, or other poultry	☐	☐	☐	☐	☐	☐	________
5.13	Foods containing beef and veal	☐	☐	☐	☐	☐	☐	________
5.14	Foods containing fish or shellfish	☐	☐	☐	☐	☐	☐	________

If your baby consumed any of the solid foods outlined above, please provide some additional information about what those foods were with respect to each category. This may include the **name of the food**, the **brand of the product** (where applicable) and whether the food was **modified** (for example: low-fat, added calcium, wholemeal etc.).[D] Please also list any foods your baby consumed that do not fall in the above categories.

5.15

[D] Refer to "Footnote D: Instructions for Question 5" on Page 5 for further clarification about answer this question

What type of methods we used to cook your baby's solid foods (tick all that applies)?

5.16	What type of methods we used to cook your baby's solid foods (tick all that applies)?			
	☐ Did not cook	☐ Baking	☐ Steaming	
	☐ Grilling	☐ Roasting	☐ Boiling	
	☐ Stewing	☐ Frying	☐ BBQ	
	☐ Microwaving	☐ Other___________________________		

6. The following questions relates to the use of dummies/pacifiers.

6.1	Do you regularly offer your baby a dummy/pacifier?	
	☐ Yes	☐ No
6.2	Did you use any sweeteners on the dummy/pacifier?	
	☐ No	☐ Yes, honey
	☐ Yes, other ____	

7. The following questions relate to occasion when your baby was given **supplements and medications**.[E] Please state the brand, what the volume of a "dose" was (e.g. 5 mL, 1 tablet, etc.), how many doses were given each day and on how many days it was given **this week**.

		Dose volume	Doses per day	Days
7.1	<u>Supplements</u>			
	☐ Multivitamin (brand:____________________)	_____	_____	_____
	☐ Omega-3/fish oil (brand:_________________)	_____	_____	_____
	☐ Iron (brand:____________________________)	_____	_____	_____
	☐ Other:_________________________________	_____	_____	_____
7.2	<u>Antibiotics</u>			
	Name:____________________________________	_____	_____	_____
	Name:____________________________________	_____	_____	_____
	Name:____________________________________	_____	_____	_____
7.3	<u>Other as outlined in *Footnote E* (please specify)</u>			
	Name:____________________________________	_____	_____	_____
	Name:____________________________________	_____	_____	_____
	Name:____________________________________	_____	_____	_____

[E] Refer to "Footnote E: Instructions for Question 7" on Page 6 for further clarification about answer this question

8. Please describe your child's health this week, specifying any **fevers** and/or illnesses that required you to visit a healthcare profession (e.g. child health nurse, doctor, naturopath, etc.)

9. Did your child receive any vaccinations this week, either scheduled or optional? If you child was not given the vaccination as per the National Immunisation Program (NIP) guidelines (for example, one particular vaccine was not given but others were) please provide details.

	Description	Timing	As per NIP guidelines	Variation of NIP guidelines (give details)
9.1	Scheduled	Birth	☐	
		2 months	☐	
		4 months	☐	
		6 months	☐	
		12 months	☐	
9.2	Optional	Influenza	☐	
		Pneumococcal 13vPCV	☐	
		Pneumococcal 23vPPV	☐	
		Hepatitis A	☐	
		Other:__________________	☐	

Your baby is 7 weeks old

Completion date: ____/____/____

1. In the past 7 days, how often was your baby feed each item listed below on average? Consider everyone that has feed the baby. Include snacks and night-time feeds.[A]

		Number feeds per day	Number of days given	Not given
1.1	Breast milk via breast feeding	_____	_____	☐
1.2	Expressed breast milk	_____	_____	☐
1.3	Formula	_____	_____	☐
1.4	Water	_____	_____	☐
1.5	Sugar water	_____	_____	☐
1.6	Cow's milk*	_____	_____	☐
1.7	Other milk**	_____	_____	☐
1.8	Fruit juices	_____	_____	☐
1.9	Other drinks‡	_____	_____	☐

* Includes plain, low-fat, evaporated and flavoured varieties of cow's milk. Include milk added to other foods such as cereal.

** Such as soy, rice, almond or goat milk. Include milk added to other foods such as cereal.

‡ Includes vegetable juice, soft drinks, cordial, sports drinks, yoghurt drinks, tea, coffee, etc.

2. The following questions relate to occasions when your baby consumed **breast milk**.

☐ *My baby did not consume breast milk this week (proceed to Question 3).*

2.1 Average feed duration when being breast feed:

_____________minutes

2.2 Average volume *consumed* when provided expressed breast milk:

_____________mL

☐ Never provided expressed breast milk *(proceed to Question 2.5)*

2.3 How long was expressed breast milk stored before it was fed to your baby (tick all that applies)?

☐ Kept at room temperature ☐ 1 day or less in refrigerator

☐ 2-3 days in refrigerator ☐ 3-4 days in refrigerator

☐ 5-7 days in refrigerator ☐ Frozen (for any length of time)

2.4 On the majority of occasions, how were bottles containing expressed breast milk warmed (tick only one)?

☐ Did not warm ☐ Hot water[ψ]\bottle warmer

☐ Microwave ☐ Other_____________________________

[A] Refer to "Footnote A: Instructions for Question 1" on Page 2 for further clarification about answer this question

2.5	Did you (the baby's mother) take any antibiotics this week? Please provide details about the type of antibiotic and dosage as described in Question 7.<br>☐ No ☐ Unsure<br>☐ Yes Type: ______________________________<br>Dose volume: ________________________<br>Doses per day: _______________________<br>Number of days: ______________________

Ψ When bottle containing the milk is placed into a container of hot water

3. The following questions relate to occasions when your baby consumed **formula**.
   ☐ *My baby did not consume formula this week (proceed to Question 4).*

3.1	Average volume consumed with provided formula:<br>_____________mL
3.2	What were the brand(s) and product name(s) of the formula(s) your baby been given. For example, "Wyeth S-26 GOLD Newborn" or "Karicare Aptamil® Delact"<br>1.____<br>2.____<br>3.____<br>☐ Unknown
3.3	Was the formula prepared according to the manufacturer's instructions?<br>☐ Yes<br>☐ No (please provide details of how the formula was prepared)<br>____<br>____<br>____
3.5	On the majority of occasions, how were bottles containing formula warmed (tick only one)?<br>☐ Did not warm ☐ Prepared with hot water<br>☐ Hot waterΨ\bottle warmer ☐ Microwave<br>☐ Other_____________________________

Ψ When bottle containing the milk is placed into a container of hot water

4. The following question relates to occasions when your baby consumed **drinks other than breast milk, formula or plain water**. This includes cow's milk or milk from other non-dairy sources as well as fruit juices, vegetable juices, cordials, soft drinks, etc. Please state what the drink(s) were, the **brand of the product** (where applicable) and how much your baby consumed on occasions

these drinks were provided. Drink additives should also be included.[B] *If your baby has never received drinks other than breast milk, formula and water proceed to question 5.*

5. The following questions relate to occasions when your baby consumed **solid foods** this week. Please indicate which of the follow foods your baby received and, on each occasion your baby was offered that food, the average amount he/she consumed with respect to a "spoon" or "mound" code as indicated in the Instructions.[C]

		Not at all	1-3 times per week	4-6 times per week	1-2 times per day	3-4 times per day	5 or more times per day	Average amount eaten per occasion
5.1	Vegetables including potato and baked beans	☐	☐	☐	☐	☐	☐	________
5.2	Fruit	☐	☐	☐	☐	☐	☐	________
5.3	Egg	☐	☐	☐	☐	☐	☐	________
5.4	Dairy products including butter, cheeses, cream, yoghurt, custard and ice cream	☐	☐	☐	☐	☐	☐	________
5.5	Wheat containing products including breads, biscuits, rusks, cakes, breakfast cereals, pastries, pasta, couscous and dim sims	☐	☐	☐	☐	☐	☐	________
5.6	Rice and/or rice containing products including rice cereals (e.g. Farax), rice cakes, rice noodles and rice flour	☐	☐	☐	☐	☐	☐	________
5.7	Soy containing products including tofu, miso, soy sauce, soy flour and soy-based dairy substitutes	☐	☐	☐	☐	☐	☐	________
5.8	Corn and/or corn containing products including popcorn, polenta, corn flakes, corn tortillas and corn flour (including products thickened with e.g. gravy)	☐	☐	☐	☐	☐	☐	________

[B] Refer to "Footnote B: Instructions for Question 4" on Page 3 for further clarification about answer this question
[C] Refer to "Footnote C: Instructions for Question 5" on Page 3 for further clarification about answer this question

		Not at all	1-3 times per week	4-6 times per week	1-2 times per day	3-4 times per day	5 or more times per day	Average amount eaten per occasion
5.9	Oats or oat containing products including porridge, oatmeal, muesli, ANZAC biscuits, bran and oat flour	☐	☐	☐	☐	☐	☐	________
5.10	Barley or rye containing products including barley water, many minestrone-style soups and rye bread	☐	☐	☐	☐	☐	☐	________
5.11	Nuts including peanut butter	☐	☐	☐	☐	☐	☐	________
5.12	Foods containing pork, lamb, chicken, turkey, or other poultry	☐	☐	☐	☐	☐	☐	________
5.13	Foods containing beef and veal	☐	☐	☐	☐	☐	☐	________
5.14	Foods containing fish or shellfish	☐	☐	☐	☐	☐	☐	________

If your baby consumed any of the solid foods outlined above, please provide some additional information about what those foods were with respect to each category. This may include the **name of the food**, the **brand of the product** (where applicable) and whether the food was **modified** (for example: low-fat, added calcium, wholemeal etc.).[D] Please also list any foods your baby consumed that do not fall in the above categories.

5.15

[D] Refer to "Footnote D: Instructions for Question 5" on Page 5 for further clarification about answer this question

What type of methods we used to cook your baby's solid foods (tick all that applies)?

5.16	What type of methods we used to cook your baby's solid foods (tick all that applies)?			
	☐ Did not cook	☐ Baking	☐ Steaming	
	☐ Grilling	☐ Roasting	☐ Boiling	
	☐ Stewing	☐ Frying	☐ BBQ	
	☐ Microwaving	☐ Other___________________________________		

6. The following questions relates to the use of dummies/pacifiers.

6.1	Do you regularly offer your baby a dummy/pacifier?	
	☐ Yes	☐ No
6.2	Did you use any sweeteners on the dummy/pacifier?	
	☐ No	☐ Yes, honey
	☐ Yes, other ____	

7. The following questions relate to occasion when your baby was given **supplements and medications**.[E] Please state the brand, what the volume of a "dose" was (e.g. 5 mL, 1 tablet, etc.), how many doses were given each day and on how many days it was given **this week**.

		Dose volume	Doses per day	Days
7.1	Supplements			
	☐ Multivitamin (brand:____________________)	_____	_____	_____
	☐ Omega-3/fish oil (brand:_________________)	_____	_____	_____
	☐ Iron (brand:____________________________)	_____	_____	_____
	☐ Other:_________________________________	_____	_____	_____
7.2	Antibiotics			
	Name:____________________________________	_____	_____	_____
	Name:____________________________________	_____	_____	_____
	Name:____________________________________	_____	_____	_____
7.3	Other as outlined in *Footnote E* (please specify)			
	Name:____________________________________	_____	_____	_____
	Name:____________________________________	_____	_____	_____
	Name:____________________________________	_____	_____	_____

[E] Refer to "Footnote E: Instructions for Question 7" on Page 6 for further clarification about answer this question

8. Please describe your child's health this week, specifying any **fevers** and/or illnesses that required you to visit a healthcare profession (e.g. child health nurse, doctor, naturopath, etc.)

9. Did your child receive any vaccinations this week, either scheduled or optional? If you child was not given the vaccination as per the National Immunisation Program (NIP) guidelines (for example, one particular vaccine was not given but others were) please provide details.

	Description	Timing	As per NIP guidelines	Variation of NIP guidelines (give details)
9.1	Scheduled	Birth	☐	
		2 months	☐	
		4 months	☐	
		6 months	☐	
		12 months	☐	
9.2	Optional	Influenza	☐	
		Pneumococcal 13vPCV	☐	
		Pneumococcal 23vPPV	☐	
		Hepatitis A	☐	
		Other:_________________	☐	

Your baby is 8 weeks old

Completion date: ____/____/____

1. In the past 7 days, how often was your baby feed each item listed below on average? Consider everyone that has feed the baby. Include snacks and night-time feeds.[A]

		Number feeds per day	Number of days given	Not given
1.1	Breast milk via breast feeding	_____	_____	☐
1.2	Expressed breast milk	_____	_____	☐
1.3	Formula	_____	_____	☐
1.4	Water	_____	_____	☐
1.5	Sugar water	_____	_____	☐
1.6	Cow's milk*	_____	_____	☐
1.7	Other milk**	_____	_____	☐
1.8	Fruit juices	_____	_____	☐
1.9	Other drinks‡	_____	_____	☐

* Includes plain, low-fat, evaporated and flavoured varieties of cow's milk. Include milk added to other foods such as cereal.

** Such as soy, rice, almond or goat milk. Include milk added to other foods such as cereal.

‡ Includes vegetable juice, soft drinks, cordial, sports drinks, yoghurt drinks, tea, coffee, etc.

2. The following questions relate to occasions when your baby consumed **breast milk**.

☐ *My baby did not consume breast milk this week (proceed to Question 3).*

2.1 Average feed duration when being breast feed:

_____________minutes

2.2 Average volume *consumed* when provided expressed breast milk:

_____________mL

☐ Never provided expressed breast milk *(proceed to Question 2.5)*

2.3 How long was expressed breast milk stored before it was fed to your baby (tick all that applies)?

☐ Kept at room temperature ☐ 1 day or less in refrigerator
☐ 2-3 days in refrigerator ☐ 3-4 days in refrigerator
☐ 5-7 days in refrigerator ☐ Frozen (for any length of time)

2.4 On the majority of occasions, how were bottles containing expressed breast milk warmed (tick only one)?

☐ Did not warm ☐ Hot water[Ψ]\bottle warmer
☐ Microwave ☐ Other_____________________________

[A] Refer to "Footnote A: Instructions for Question 1" on Page 2 for further clarification about answer this question

2.5	Did you (the baby's mother) take any antibiotics this week? Please provide details about the type of antibiotic and dosage as described in Question 7.<br>☐ No ☐ Unsure<br>☐ Yes Type: ______________________________<br>Dose volume: ________________________<br>Doses per day: ______________________<br>Number of days: ______________________

Ψ When bottle containing the milk is placed into a container of hot water

3. The following questions relate to occasions when your baby consumed **formula**.
   ☐ *My baby did not consume formula this week (proceed to Question 4).*

3.1	Average volume consumed with provided formula:<br>_____________mL
3.2	What were the brand(s) and product name(s) of the formula(s) your baby been given. For example, "Wyeth S-26 GOLD Newborn" or "Karicare Aptamil® Delact"<br>1.____<br>2.____<br>3.____<br>☐ Unknown
3.3	Was the formula prepared according to the manufacturer's instructions?<br>☐ Yes<br>☐ No (please provide details of how the formula was prepared)<br>____<br>____<br>____
3.5	On the majority of occasions, how were bottles containing formula warmed (tick only one)?<br>☐ Did not warm ☐ Prepared with hot water<br>☐ Hot waterΨ\bottle warmer ☐ Microwave<br>☐ Other_____________________________

Ψ When bottle containing the milk is placed into a container of hot water

4. The following question relates to occasions when your baby consumed **drinks other than breast milk, formula or plain water**. This includes cow's milk or milk from other non-dairy sources as well as fruit juices, vegetable juices, cordials, soft drinks, etc. Please state what the drink(s) were, the **brand of the product** (where applicable) and how much your baby consumed on occasions

these drinks were provided. Drink additives should also be included.[B] *If your baby has never received drinks other than breast milk, formula and water proceed to question 5.*

5. The following questions relate to occasions when your baby consumed **solid foods** this week. Please indicate which of the follow foods your baby received and, on each occasion your baby was offered that food, the average amount he/she consumed with respect to a "spoon" or "mound" code as indicated in the Instructions.[C]

		Not at all	1-3 times per week	4-6 times per week	1-2 times per day	3-4 times per day	5 or more times per day	Average amount eaten per occasion
5.1	Vegetables including potato and baked beans	☐	☐	☐	☐	☐	☐	________
5.2	Fruit	☐	☐	☐	☐	☐	☐	________
5.3	Egg	☐	☐	☐	☐	☐	☐	________
5.4	Dairy products including butter, cheeses, cream, yoghurt, custard and ice cream	☐	☐	☐	☐	☐	☐	________
5.5	Wheat containing products including breads, biscuits, rusks, cakes, breakfast cereals, pastries, pasta, couscous and dim sims	☐	☐	☐	☐	☐	☐	________
5.6	Rice and/or rice containing products including rice cereals (e.g. Farax), rice cakes, rice noodles and rice flour	☐	☐	☐	☐	☐	☐	________
5.7	Soy containing products including tofu, miso, soy sauce, soy flour and soy-based dairy substitutes	☐	☐	☐	☐	☐	☐	________
5.8	Corn and/or corn containing products including popcorn, polenta, corn flakes, corn tortillas and corn flour (including products thickened with e.g. gravy)	☐	☐	☐	☐	☐	☐	________

[B] Refer to "Footnote B: Instructions for Question 4" on Page 3 for further clarification about answer this question
[C] Refer to "Footnote C: Instructions for Question 5" on Page 3 for further clarification about answer this question

		Not at all	1-3 times per week	4-6 times per week	1-2 times per day	3-4 times per day	5 or more times per day	Average amount eaten per occasion
5.9	Oats or oat containing products including porridge, oatmeal, muesli, ANZAC biscuits, bran and oat flour	☐	☐	☐	☐	☐	☐	________
5.10	Barley or rye containing products including barley water, many minestrone-style soups and rye bread	☐	☐	☐	☐	☐	☐	________
5.11	Nuts including peanut butter	☐	☐	☐	☐	☐	☐	________
5.12	Foods containing pork, lamb, chicken, turkey, or other poultry	☐	☐	☐	☐	☐	☐	________
5.13	Foods containing beef and veal	☐	☐	☐	☐	☐	☐	________
5.14	Foods containing fish or shellfish	☐	☐	☐	☐	☐	☐	________

If your baby consumed any of the solid foods outlined above, please provide some additional information about what those foods were with respect to each category. This may include the **name of the food**, the **brand of the product** (where applicable) and whether the food was **modified** (for example: low-fat, added calcium, wholemeal etc.).[D] Please also list any foods your baby consumed that do not fall in the above categories.

5.15

[D] Refer to "Footnote D: Instructions for Question 5" on Page 5 for further clarification about answer this question

What type of methods we used to cook your baby's solid foods (tick all that applies)?

5.16	What type of methods we used to cook your baby's solid foods (tick all that applies)?<br>☐ Did not cook ☐ Baking ☐ Steaming<br>☐ Grilling ☐ Roasting ☐ Boiling<br>☐ Stewing ☐ Frying ☐ BBQ<br>☐ Microwaving ☐ Other___________________________________

6. The following questions relates to the use of dummies/pacifiers.

6.1	Do you regularly offer your baby a dummy/pacifier?<br>☐ Yes ☐ No
6.2	Did you use any sweeteners on the dummy/pacifier?<br>☐ No ☐ Yes, honey<br>☐ Yes, other ____

7. The following questions relate to occasion when your baby was given **supplements and medications**.[E] Please state the brand, what the volume of a "dose" was (e.g. 5 mL, 1 tablet, etc.), how many doses were given each day and on how many days it was given **this week**.

		Dose volume	Doses per day	Days
7.1	Supplements			
	☐ Multivitamin (brand:____________________)	_____	_____	_____
	☐ Omega-3/fish oil (brand:_________________)	_____	_____	_____
	☐ Iron (brand:____________________________)	_____	_____	_____
	☐ Other:_________________________________	_____	_____	_____
7.2	Antibiotics			
	Name:____________________________________	_____	_____	_____
	Name:____________________________________	_____	_____	_____
	Name:____________________________________	_____	_____	_____
7.3	Other as outlined in *Footnote E* (please specify)			
	Name:____________________________________	_____	_____	_____
	Name:____________________________________	_____	_____	_____
	Name:____________________________________	_____	_____	_____

[E] Refer to "Footnote E: Instructions for Question 7" on Page 6 for further clarification about answer this question

8. Please describe your child's health this week, specifying any **fevers** and/or illnesses that required you to visit a healthcare profession (e.g. child health nurse, doctor, naturopath, etc.)

9. Did your child receive any vaccinations this week, either scheduled or optional? If you child was not given the vaccination as per the National Immunisation Program (NIP) guidelines (for example, one particular vaccine was not given but others were) please provide details.

	Description	Timing	As per NIP guidelines	Variation of NIP guidelines (give details)
9.1	Scheduled	Birth	☐	
		2 months	☐	
		4 months	☐	
		6 months	☐	
		12 months	☐	
9.2	Optional	Influenza	☐	
		Pneumococcal 13vPCV	☐	
		Pneumococcal 23vPPV	☐	
		Hepatitis A	☐	
		Other:_________________	☐	

Your baby is 9 weeks old

Completion date: ____/____/____

1. In the past 7 days, how often was your baby feed each item listed below on average? Consider everyone that has feed the baby. Include snacks and night-time feeds.[A]

		Number feeds per day	Number of days given	Not given
1.1	Breast milk via breast feeding	_____	_____	☐
1.2	Expressed breast milk	_____	_____	☐
1.3	Formula	_____	_____	☐
1.4	Water	_____	_____	☐
1.5	Sugar water	_____	_____	☐
1.6	Cow's milk*	_____	_____	☐
1.7	Other milk**	_____	_____	☐
1.8	Fruit juices	_____	_____	☐
1.9	Other drinks‡	_____	_____	☐

* Includes plain, low-fat, evaporated and flavoured varieties of cow's milk. Include milk added to other foods such as cereal.

** Such as soy, rice, almond or goat milk. Include milk added to other foods such as cereal.

‡ Includes vegetable juice, soft drinks, cordial, sports drinks, yoghurt drinks, tea, coffee, etc.

2. The following questions relate to occasions when your baby consumed **breast milk**.

☐ *My baby did not consume breast milk this week (proceed to Question 3).*

2.1 Average feed duration when being breast feed:

_____________minutes

2.2 Average volume *consumed* when provided expressed breast milk:

_____________mL

☐ Never provided expressed breast milk *(proceed to Question 2.5)*

2.3 How long was expressed breast milk stored before it was fed to your baby (tick all that applies)?

☐ Kept at room temperature ☐ 1 day or less in refrigerator
☐ 2-3 days in refrigerator ☐ 3-4 days in refrigerator
☐ 5-7 days in refrigerator ☐ Frozen (for any length of time)

2.4 On the majority of occasions, how were bottles containing expressed breast milk warmed (tick only one)?

☐ Did not warm ☐ Hot water[Ψ]\bottle warmer
☐ Microwave ☐ Other____________________________

[A] Refer to "Footnote A: Instructions for Question 1" on Page 2 for further clarification about answer this question

2.5	Did you (the baby's mother) take any antibiotics this week? Please provide details about the type of antibiotic and dosage as described in Question 7.<br>☐ No ☐ Unsure<br>☐ Yes Type: ______________________________<br>Dose volume: ________________________<br>Doses per day: _______________________<br>Number of days: ______________________

ᵠ When bottle containing the milk is placed into a container of hot water

3. The following questions relate to occasions when your baby consumed **formula**.
   ☐ *My baby did not consume formula this week (proceed to Question 4).*

3.1	Average volume consumed with provided formula:<br>_____________mL
3.2	What were the brand(s) and product name(s) of the formula(s) your baby been given. For example, "Wyeth S-26 GOLD Newborn" or "Karicare Aptamil® Delact"<br>1.____<br>2.____<br>3.____<br>☐ Unknown
3.3	Was the formula prepared according to the manufacturer's instructions?<br>☐ Yes<br>☐ No (please provide details of how the formula was prepared)<br>____<br>____<br>____
3.5	On the majority of occasions, how were bottles containing formula warmed (tick only one)?<br>☐ Did not warm ☐ Prepared with hot water<br>☐ Hot waterᵠ\bottle warmer ☐ Microwave<br>☐ Other____________________________

ᵠ When bottle containing the milk is placed into a container of hot water

4. The following question relates to occasions when your baby consumed **drinks other than breast milk, formula or plain water**. This includes cow's milk or milk from other non-dairy sources as well as fruit juices, vegetable juices, cordials, soft drinks, etc. Please state what the drink(s) were, the **brand of the product** (where applicable) and how much your baby consumed on occasions

these drinks were provided. Drink additives should also be included.[B] *If your baby has never received drinks other than breast milk, formula and water proceed to question 5.*

5. The following questions relate to occasions when your baby consumed **solid foods** this week. Please indicate which of the follow foods your baby received and, on each occasion your baby was offered that food, the average amount he/she consumed with respect to a “spoon” or “mound” code as indicated in the Instructions.[C]

		Not at all	1-3 times per week	4-6 times per week	1-2 times per day	3-4 times per day	5 or more times per day	Average amount eaten per occasion
5.1	Vegetables including potato and baked beans	☐	☐	☐	☐	☐	☐	________
5.2	Fruit	☐	☐	☐	☐	☐	☐	________
5.3	Egg	☐	☐	☐	☐	☐	☐	________
5.4	Dairy products including butter, cheeses, cream, yoghurt, custard and ice cream	☐	☐	☐	☐	☐	☐	________
5.5	Wheat containing products including breads, biscuits, rusks, cakes, breakfast cereals, pastries, pasta, couscous and dim sims	☐	☐	☐	☐	☐	☐	________
5.6	Rice and/or rice containing products including rice cereals (e.g. Farax), rice cakes, rice noodles and rice flour	☐	☐	☐	☐	☐	☐	________
5.7	Soy containing products including tofu, miso, soy sauce, soy flour and soy-based dairy substitutes	☐	☐	☐	☐	☐	☐	________
5.8	Corn and/or corn containing products including popcorn, polenta, corn flakes, corn tortillas and corn flour (including products thickened with e.g. gravy)	☐	☐	☐	☐	☐	☐	________

[B] Refer to “Footnote B: Instructions for Question 4” on Page 3 for further clarification about answer this question
[C] Refer to “Footnote C: Instructions for Question 5” on Page 3 for further clarification about answer this question

		Not at all	1-3 times per week	4-6 times per week	1-2 times per day	3-4 times per day	5 or more times per day	Average amount eaten per occasion
5.9	Oats or oat containing products including porridge, oatmeal, muesli, ANZAC biscuits, bran and oat flour	☐	☐	☐	☐	☐	☐	________
5.10	Barley or rye containing products including barley water, many minestrone-style soups and rye bread	☐	☐	☐	☐	☐	☐	________
5.11	Nuts including peanut butter	☐	☐	☐	☐	☐	☐	________
5.12	Foods containing pork, lamb, chicken, turkey, or other poultry	☐	☐	☐	☐	☐	☐	________
5.13	Foods containing beef and veal	☐	☐	☐	☐	☐	☐	________
5.14	Foods containing fish or shellfish	☐	☐	☐	☐	☐	☐	________

If your baby consumed any of the solid foods outlined above, please provide some additional information about what those foods were with respect to each category. This may include the **name of the food**, the **brand of the product** (where applicable) and whether the food was **modified** (for example: low-fat, added calcium, wholemeal etc.).[D] Please also list any foods your baby consumed that do not fall in the above categories.

5.15

[D] Refer to "Footnote D: Instructions for Question 5" on Page 5 for further clarification about answer this question

What type of methods we used to cook your baby's solid foods (tick all that applies)?

5.16	What type of methods we used to cook your baby's solid foods (tick all that applies)?<br>☐ Did not cook ☐ Baking ☐ Steaming<br>☐ Grilling ☐ Roasting ☐ Boiling<br>☐ Stewing ☐ Frying ☐ BBQ<br>☐ Microwaving ☐ Other___________________________________

6. The following questions relates to the use of dummies/pacifiers.

6.1	Do you regularly offer your baby a dummy/pacifier?<br>☐ Yes ☐ No
6.2	Did you use any sweeteners on the dummy/pacifier?<br>☐ No ☐ Yes, honey<br>☐ Yes, other ____

7. The following questions relate to occasion when your baby was given **supplements and medications**.[E] Please state the brand, what the volume of a "dose" was (e.g. 5 mL, 1 tablet, etc.), how many doses were given each day and on how many days it was given **this week**.

		Dose volume	Doses per day	Days
7.1	Supplements			
	☐ Multivitamin (brand:____________________)	_____	_____	_____
	☐ Omega-3/fish oil (brand:_________________)	_____	_____	_____
	☐ Iron (brand:____________________________)	_____	_____	_____
	☐ Other:_________________________________	_____	_____	_____
7.2	Antibiotics			
	Name:___________________________________	_____	_____	_____
	Name:___________________________________	_____	_____	_____
	Name:___________________________________	_____	_____	_____
7.3	Other as outlined in *Footnote E* (please specify)			
	Name:___________________________________	_____	_____	_____
	Name:___________________________________	_____	_____	_____
	Name:___________________________________	_____	_____	_____

[E] Refer to "Footnote E: Instructions for Question 7" on Page 6 for further clarification about answer this question

8. Please describe your child's health this week, specifying any **fevers** and/or illnesses that required you to visit a healthcare profession (e.g. child health nurse, doctor, naturopath, etc.)

9. Did your child receive any vaccinations this week, either scheduled or optional? If you child was not given the vaccination as per the National Immunisation Program (NIP) guidelines (for example, one particular vaccine was not given but others were) please provide details.

	Description	Timing	As per NIP guidelines	Variation of NIP guidelines (give details)
9.1	Scheduled	Birth	☐	
		2 months	☐	
		4 months	☐	
		6 months	☐	
		12 months	☐	
9.2	Optional	Influenza	☐	
		Pneumococcal 13vPCV	☐	
		Pneumococcal 23vPPV	☐	
		Hepatitis A	☐	
		Other:__________________	☐	

Your baby is 10 weeks old

Completion date: ____/____/____

1. In the past 7 days, how often was your baby feed each item listed below on average? Consider everyone that has feed the baby. Include snacks and night-time feeds.[A]

		Number feeds per day	Number of days given	Not given
1.1	Breast milk via breast feeding	_____	_____	☐
1.2	Expressed breast milk	_____	_____	☐
1.3	Formula	_____	_____	☐
1.4	Water	_____	_____	☐
1.5	Sugar water	_____	_____	☐
1.6	Cow's milk*	_____	_____	☐
1.7	Other milk**	_____	_____	☐
1.8	Fruit juices	_____	_____	☐
1.9	Other drinks‡	_____	_____	☐

* Includes plain, low-fat, evaporated and flavoured varieties of cow's milk. Include milk added to other foods such as cereal.

** Such as soy, rice, almond or goat milk. Include milk added to other foods such as cereal.

‡ Includes vegetable juice, soft drinks, cordial, sports drinks, yoghurt drinks, tea, coffee, etc.

2. The following questions relate to occasions when your baby consumed **breast milk**.

☐ *My baby did not consume breast milk this week (proceed to Question 3).*

2.1 Average feed duration when being breast feed:

_____________minutes

2.2 Average volume *consumed* when provided expressed breast milk:

_____________mL

☐ Never provided expressed breast milk *(proceed to Question 2.5)*

2.3 How long was expressed breast milk stored before it was fed to your baby (tick all that applies)?

☐ Kept at room temperature ☐ 1 day or less in refrigerator
☐ 2-3 days in refrigerator ☐ 3-4 days in refrigerator
☐ 5-7 days in refrigerator ☐ Frozen (for any length of time)

2.4 On the majority of occasions, how were bottles containing expressed breast milk warmed (tick only one)?

☐ Did not warm ☐ Hot water[Ψ]\bottle warmer
☐ Microwave ☐ Other_____________________________

[A] Refer to "Footnote A: Instructions for Question 1" on Page 2 for further clarification about answer this question

2.5	Did you (the baby's mother) take any antibiotics this week? Please provide details about the type of antibiotic and dosage as described in Question 7.<br>☐ No ☐ Unsure<br>☐ Yes Type: ______________________________<br>Dose volume: ________________________<br>Doses per day: _______________________<br>Number of days: ______________________

ᵠ When bottle containing the milk is placed into a container of hot water

3. The following questions relate to occasions when your baby consumed **formula**.

   ☐ *My baby did not consume formula this week (proceed to Question 4).*

3.1	Average volume consumed with provided formula:<br>_____________mL
3.2	What were the brand(s) and product name(s) of the formula(s) your baby been given. For example, "Wyeth S-26 GOLD Newborn" or "Karicare Aptamil® Delact"<br>1.____<br>2.____<br>3.____<br>☐ Unknown
3.3	Was the formula prepared according to the manufacturer's instructions?<br>☐ Yes<br>☐ No (please provide details of how the formula was prepared)<br>____<br>____<br>____
3.5	On the majority of occasions, how were bottles containing formula warmed (tick only one)?<br>☐ Did not warm ☐ Prepared with hot water<br>☐ Hot waterᵠ\bottle warmer ☐ Microwave<br>☐ Other____________________________

ᵠ When bottle containing the milk is placed into a container of hot water

4. The following question relates to occasions when your baby consumed **drinks other than breast milk, formula or plain water**. This includes cow's milk or milk from other non-dairy sources as well as fruit juices, vegetable juices, cordials, soft drinks, etc. Please state what the drink(s) were, the **brand of the product** (where applicable) and how much your baby consumed on occasions

these drinks were provided. Drink additives should also be included.[B] *If your baby has never received drinks other than breast milk, formula and water proceed to question 5.*

5. The following questions relate to occasions when your baby consumed **solid foods** this week. Please indicate which of the follow foods your baby received and, on each occasion your baby was offered that food, the average amount he/she consumed with respect to a "spoon" or "mound" code as indicated in the Instructions.[C]

		Not at all	1-3 times per week	4-6 times per week	1-2 times per day	3-4 times per day	5 or more times per day	Average amount eaten per occasion
5.1	Vegetables including potato and baked beans	☐	☐	☐	☐	☐	☐	________
5.2	Fruit	☐	☐	☐	☐	☐	☐	________
5.3	Egg	☐	☐	☐	☐	☐	☐	________
5.4	Dairy products including butter, cheeses, cream, yoghurt, custard and ice cream	☐	☐	☐	☐	☐	☐	________
5.5	Wheat containing products including breads, biscuits, rusks, cakes, breakfast cereals, pastries, pasta, couscous and dim sims	☐	☐	☐	☐	☐	☐	________
5.6	Rice and/or rice containing products including rice cereals (e.g. Farax), rice cakes, rice noodles and rice flour	☐	☐	☐	☐	☐	☐	________
5.7	Soy containing products including tofu, miso, soy sauce, soy flour and soy-based dairy substitutes	☐	☐	☐	☐	☐	☐	________
5.8	Corn and/or corn containing products including popcorn, polenta, corn flakes, corn tortillas and corn flour (including products thickened with e.g. gravy)	☐	☐	☐	☐	☐	☐	________

[B] Refer to "Footnote B: Instructions for Question 4" on Page 3 for further clarification about answer this question
[C] Refer to "Footnote C: Instructions for Question 5" on Page 3 for further clarification about answer this question

		Not at all	1-3 times per week	4-6 times per week	1-2 times per day	3-4 times per day	5 or more times per day	Average amount eaten per occasion
5.9	Oats or oat containing products including porridge, oatmeal, muesli, ANZAC biscuits, bran and oat flour	☐	☐	☐	☐	☐	☐	________
5.10	Barley or rye containing products including barley water, many minestrone-style soups and rye bread	☐	☐	☐	☐	☐	☐	________
5.11	Nuts including peanut butter	☐	☐	☐	☐	☐	☐	________
5.12	Foods containing pork, lamb, chicken, turkey, or other poultry	☐	☐	☐	☐	☐	☐	________
5.13	Foods containing beef and veal	☐	☐	☐	☐	☐	☐	________
5.14	Foods containing fish or shellfish	☐	☐	☐	☐	☐	☐	________

If your baby consumed any of the solid foods outlined above, please provide some additional information about what those foods were with respect to each category. This may include the **name of the food**, the **brand of the product** (where applicable) and whether the food was **modified** (for example: low-fat, added calcium, wholemeal etc.).[D] Please also list any foods your baby consumed that do not fall in the above categories.

5.15

[D] Refer to "Footnote D: Instructions for Question 5" on Page 5 for further clarification about answer this question

What type of methods we used to cook your baby's solid foods (tick all that applies)?

5.16	What type of methods we used to cook your baby's solid foods (tick all that applies)?<br>☐ Did not cook ☐ Baking ☐ Steaming<br>☐ Grilling ☐ Roasting ☐ Boiling<br>☐ Stewing ☐ Frying ☐ BBQ<br>☐ Microwaving ☐ Other___________________________

6. The following questions relates to the use of dummies/pacifiers.

6.1	Do you regularly offer your baby a dummy/pacifier?<br>☐ Yes ☐ No
6.2	Did you use any sweeteners on the dummy/pacifier?<br>☐ No ☐ Yes, honey<br>☐ Yes, other ___________________________

7. The following questions relate to occasion when your baby was given **supplements and medications**.[E] Please state the brand, what the volume of a "dose" was (e.g. 5 mL, 1 tablet, etc.), how many doses were given each day and on how many days it was given **this week**.

		Dose volume	Doses per day	Days
7.1	<u>Supplements</u>			
	☐ Multivitamin (brand:___________________)	_____	_____	_____
	☐ Omega-3/fish oil (brand:________________)	_____	_____	_____
	☐ Iron (brand:__________________________)	_____	_____	_____
	☐ Other:______________________________	_____	_____	_____
7.2	<u>Antibiotics</u>			
	Name:________________________________	_____	_____	_____
	Name:________________________________	_____	_____	_____
	Name:________________________________	_____	_____	_____
7.3	<u>Other as outlined in *Footnote E* (please specify)</u>			
	Name:________________________________	_____	_____	_____
	Name:________________________________	_____	_____	_____
	Name:________________________________	_____	_____	_____

[E] Refer to "Footnote E: Instructions for Question 7" on Page 6 for further clarification about answer this question

8. Please describe your child's health this week, specifying any **fevers** and/or illnesses that required you to visit a healthcare profession (e.g. child health nurse, doctor, naturopath, etc.)

9. Did your child receive any vaccinations this week, either scheduled or optional? If you child was not given the vaccination as per the National Immunisation Program (NIP) guidelines (for example, one particular vaccine was not given but others were) please provide details.

	Description	Timing	As per NIP guidelines	Variation of NIP guidelines (give details)
9.1	Scheduled	Birth	☐	
		2 months	☐	
		4 months	☐	
		6 months	☐	
		12 months	☐	
9.2	Optional	Influenza	☐	
		Pneumococcal 13vPCV	☐	
		Pneumococcal 23vPPV	☐	
		Hepatitis A	☐	
		Other:__________________	☐	

Your baby is 11 weeks old

Completion date: ____/____/____

1. In the past 7 days, how often was your baby feed each item listed below on average? Consider everyone that has feed the baby. Include snacks and night-time feeds.[A]

		Number feeds per day	Number of days given	Not given
1.1	Breast milk via breast feeding	_____	_____	☐
1.2	Expressed breast milk	_____	_____	☐
1.3	Formula	_____	_____	☐
1.4	Water	_____	_____	☐
1.5	Sugar water	_____	_____	☐
1.6	Cow's milk*	_____	_____	☐
1.7	Other milk**	_____	_____	☐
1.8	Fruit juices	_____	_____	☐
1.9	Other drinks‡	_____	_____	☐

* Includes plain, low-fat, evaporated and flavoured varieties of cow's milk. Include milk added to other foods such as cereal.

** Such as soy, rice, almond or goat milk. Include milk added to other foods such as cereal.

‡ Includes vegetable juice, soft drinks, cordial, sports drinks, yoghurt drinks, tea, coffee, etc.

2. The following questions relate to occasions when your baby consumed **breast milk**.

☐ *My baby did not consume breast milk this week (proceed to Question 3).*

2.1 Average feed duration when being breast feed:

_____________minutes

2.2 Average volume *consumed* when provided expressed breast milk:

_____________mL

☐ Never provided expressed breast milk *(proceed to Question 2.5)*

2.3 How long was expressed breast milk stored before it was fed to your baby (tick all that applies)?

☐ Kept at room temperature ☐ 1 day or less in refrigerator

☐ 2-3 days in refrigerator ☐ 3-4 days in refrigerator

☐ 5-7 days in refrigerator ☐ Frozen (for any length of time)

2.4 On the majority of occasions, how were bottles containing expressed breast milk warmed (tick only one)?

☐ Did not warm ☐ Hot water[Ψ]\bottle warmer

☐ Microwave ☐ Other_____________________________

[A] Refer to "Footnote A: Instructions for Question 1" on Page 2 for further clarification about answer this question

2.5	Did you (the baby's mother) take any antibiotics this week? Please provide details about the type of antibiotic and dosage as described in Question 7.<br>☐ No ☐ Unsure<br>☐ Yes Type: ______________________________<br>Dose volume: ________________________<br>Doses per day: _______________________<br>Number of days: ______________________

Ψ When bottle containing the milk is placed into a container of hot water

3. The following questions relate to occasions when your baby consumed **formula**.

   ☐ *My baby did not consume formula this week (proceed to Question 4).*

3.1	Average volume consumed with provided formula:<br>_____________mL
3.2	What were the brand(s) and product name(s) of the formula(s) your baby been given. For example, "Wyeth S-26 GOLD Newborn" or "Karicare Aptamil® Delact"<br>1.____<br>2.____<br>3.____<br>☐ Unknown
3.3	Was the formula prepared according to the manufacturer's instructions?<br>☐ Yes<br>☐ No (please provide details of how the formula was prepared)<br>____<br>____<br>____
3.5	On the majority of occasions, how were bottles containing formula warmed (tick only one)?<br>☐ Did not warm ☐ Prepared with hot water<br>☐ Hot waterΨ\bottle warmer ☐ Microwave<br>☐ Other_____________________________

Ψ When bottle containing the milk is placed into a container of hot water

4. The following question relates to occasions when your baby consumed **drinks other than breast milk, formula or plain water**. This includes cow's milk or milk from other non-dairy sources as well as fruit juices, vegetable juices, cordials, soft drinks, etc. Please state what the drink(s) were, the **brand of the product** (where applicable) and how much your baby consumed on occasions

these drinks were provided. Drink additives should also be included.[B] *If your baby has never received drinks other than breast milk, formula and water proceed to question 5.*

5. The following questions relate to occasions when your baby consumed **solid foods** this week. Please indicate which of the follow foods your baby received and, on each occasion your baby was offered that food, the average amount he/she consumed with respect to a "spoon" or "mound" code as indicated in the Instructions.[C]

		Not at all	1-3 times per week	4-6 times per week	1-2 times per day	3-4 times per day	5 or more times per day	Average amount eaten per occasion
5.1	Vegetables including potato and baked beans	☐	☐	☐	☐	☐	☐	________
5.2	Fruit	☐	☐	☐	☐	☐	☐	________
5.3	Egg	☐	☐	☐	☐	☐	☐	________
5.4	Dairy products including butter, cheeses, cream, yoghurt, custard and ice cream	☐	☐	☐	☐	☐	☐	________
5.5	Wheat containing products including breads, biscuits, rusks, cakes, breakfast cereals, pastries, pasta, couscous and dim sims	☐	☐	☐	☐	☐	☐	________
5.6	Rice and/or rice containing products including rice cereals (e.g. Farax), rice cakes, rice noodles and rice flour	☐	☐	☐	☐	☐	☐	________
5.7	Soy containing products including tofu, miso, soy sauce, soy flour and soy-based dairy substitutes	☐	☐	☐	☐	☐	☐	________
5.8	Corn and/or corn containing products including popcorn, polenta, corn flakes, corn tortillas and corn flour (including products thickened with e.g. gravy)	☐	☐	☐	☐	☐	☐	________

[B] Refer to "Footnote B: Instructions for Question 4" on Page 3 for further clarification about answer this question
[C] Refer to "Footnote C: Instructions for Question 5" on Page 3 for further clarification about answer this question

		Not at all	1-3 times per week	4-6 times per week	1-2 times per day	3-4 times per day	5 or more times per day	Average amount eaten per occasion
5.9	Oats or oat containing products including porridge, oatmeal, muesli, ANZAC biscuits, bran and oat flour	☐	☐	☐	☐	☐	☐	________
5.10	Barley or rye containing products including barley water, many minestrone-style soups and rye bread	☐	☐	☐	☐	☐	☐	________
5.11	Nuts including peanut butter	☐	☐	☐	☐	☐	☐	________
5.12	Foods containing pork, lamb, chicken, turkey, or other poultry	☐	☐	☐	☐	☐	☐	________
5.13	Foods containing beef and veal	☐	☐	☐	☐	☐	☐	________
5.14	Foods containing fish or shellfish	☐	☐	☐	☐	☐	☐	________

If your baby consumed any of the solid foods outlined above, please provide some additional information about what those foods were with respect to each category. This may include the **name of the food**, the **brand of the product** (where applicable) and whether the food was **modified** (for example: low-fat, added calcium, wholemeal etc.).[D] Please also list any foods your baby consumed that do not fall in the above categories.

5.15

[D] Refer to "Footnote D: Instructions for Question 5" on Page 5 for further clarification about answer this question

What type of methods we used to cook your baby's solid foods (tick all that applies)?

5.16	What type of methods we used to cook your baby's solid foods (tick all that applies)?
	☐ Did not cook ☐ Baking ☐ Steaming
	☐ Grilling ☐ Roasting ☐ Boiling
	☐ Stewing ☐ Frying ☐ BBQ
	☐ Microwaving ☐ Other___________________________________

6. The following questions relates to the use of dummies/pacifiers.

6.1	Do you regularly offer your baby a dummy/pacifier?
	☐ Yes ☐ No
6.2	Did you use any sweeteners on the dummy/pacifier?
	☐ No ☐ Yes, honey
	☐ Yes, other ____

7. The following questions relate to occasion when your baby was given **supplements and medications**.[E] Please state the brand, what the volume of a "dose" was (e.g. 5 mL, 1 tablet, etc.), how many doses were given each day and on how many days it was given **this week**.

		Dose volume	Doses per day	Days
7.1	Supplements			
	☐ Multivitamin (brand:____________________)	_____	_____	_____
	☐ Omega-3/fish oil (brand:_________________)	_____	_____	_____
	☐ Iron (brand:____________________________)	_____	_____	_____
	☐ Other:_________________________________	_____	_____	_____
7.2	Antibiotics			
	Name:____________________________________	_____	_____	_____
	Name:____________________________________	_____	_____	_____
	Name:____________________________________	_____	_____	_____
7.3	Other as outlined in *Footnote E* (please specify)			
	Name:____________________________________	_____	_____	_____
	Name:____________________________________	_____	_____	_____
	Name:____________________________________	_____	_____	_____

[E] Refer to "Footnote E: Instructions for Question 7" on Page 6 for further clarification about answer this question

8. Please describe your child's health this week, specifying any **fevers** and/or illnesses that required you to visit a healthcare profession (e.g. child health nurse, doctor, naturopath, etc.)

9. Did your child receive any vaccinations this week, either scheduled or optional? If you child was not given the vaccination as per the National Immunisation Program (NIP) guidelines (for example, one particular vaccine was not given but others were) please provide details.

	Description	Timing	As per NIP guidelines	Variation of NIP guidelines (give details)
9.1	Scheduled	Birth	☐	
		2 months	☐	
		4 months	☐	
		6 months	☐	
		12 months	☐	
9.2	Optional	Influenza	☐	
		Pneumococcal 13vPCV	☐	
		Pneumococcal 23vPPV	☐	
		Hepatitis A	☐	
		Other:_________________	☐	

Your baby is 12 weeks old

Completion date: ____/____/____

1. In the past 7 days, how often was your baby feed each item listed below on average? Consider everyone that has feed the baby. Include snacks and night-time feeds.[A]

		Number feeds per day	Number of days given	Not given
1.1	Breast milk via breast feeding	_____	_____	☐
1.2	Expressed breast milk	_____	_____	☐
1.3	Formula	_____	_____	☐
1.4	Water	_____	_____	☐
1.5	Sugar water	_____	_____	☐
1.6	Cow's milk*	_____	_____	☐
1.7	Other milk**	_____	_____	☐
1.8	Fruit juices	_____	_____	☐
1.9	Other drinks‡	_____	_____	☐

* Includes plain, low-fat, evaporated and flavoured varieties of cow's milk. Include milk added to other foods such as cereal.

** Such as soy, rice, almond or goat milk. Include milk added to other foods such as cereal.

‡ Includes vegetable juice, soft drinks, cordial, sports drinks, yoghurt drinks, tea, coffee, etc.

2. The following questions relate to occasions when your baby consumed **breast milk**.

☐ *My baby did not consume breast milk this week (proceed to Question 3).*

2.1 Average feed duration when being breast feed:

_____________minutes

2.2 Average volume *consumed* when provided expressed breast milk:

_____________mL

☐ Never provided expressed breast milk *(proceed to Question 2.5)*

2.3 How long was expressed breast milk stored before it was fed to your baby (tick all that applies)?

☐ Kept at room temperature ☐ 1 day or less in refrigerator
☐ 2-3 days in refrigerator ☐ 3-4 days in refrigerator
☐ 5-7 days in refrigerator ☐ Frozen (for any length of time)

2.4 On the majority of occasions, how were bottles containing expressed breast milk warmed (tick only one)?

☐ Did not warm ☐ Hot water[Ψ]\bottle warmer
☐ Microwave ☐ Other____________________________

[A] Refer to "Footnote A: Instructions for Question 1" on Page 2 for further clarification about answer this question

2.5	Did you (the baby's mother) take any antibiotics this week? Please provide details about the type of antibiotic and dosage as described in Question 7.<br>☐ No ☐ Unsure<br>☐ Yes Type: ________________<br>Dose volume: ________________<br>Doses per day: ________________<br>Number of days: ________________

Ψ When bottle containing the milk is placed into a container of hot water

3. The following questions relate to occasions when your baby consumed **formula**.

   ☐ *My baby did not consume formula this week (proceed to Question 4).*

3.1	Average volume consumed with provided formula:<br>_____________mL
3.2	What were the brand(s) and product name(s) of the formula(s) your baby been given. For example, "Wyeth S-26 GOLD Newborn" or "Karicare Aptamil® Delact"<br>1.________________<br>2.________________<br>3.________________<br>☐ Unknown
3.3	Was the formula prepared according to the manufacturer's instructions?<br>☐ Yes<br>☐ No (please provide details of how the formula was prepared)<br>________________<br>________________<br>________________
3.5	On the majority of occasions, how were bottles containing formula warmed (tick only one)?<br>☐ Did not warm ☐ Prepared with hot water<br>☐ Hot waterΨ\bottle warmer ☐ Microwave<br>☐ Other________________

Ψ When bottle containing the milk is placed into a container of hot water

4. The following question relates to occasions when your baby consumed **drinks other than breast milk, formula or plain water**. This includes cow's milk or milk from other non-dairy sources as well as fruit juices, vegetable juices, cordials, soft drinks, etc. Please state what the drink(s) were, the **brand of the product** (where applicable) and how much your baby consumed on occasions

these drinks were provided. Drink additives should also be included.[B] *If your baby has never received drinks other than breast milk, formula and water proceed to question 5.*

5. The following questions relate to occasions when your baby consumed **solid foods** this week. Please indicate which of the follow foods your baby received and, on each occasion your baby was offered that food, the average amount he/she consumed with respect to a "spoon" or "mound" code as indicated in the Instructions.[C]

		Not at all	1-3 times per week	4-6 times per week	1-2 times per day	3-4 times per day	5 or more times per day	Average amount eaten per occasion
5.1	Vegetables including potato and baked beans	☐	☐	☐	☐	☐	☐	________
5.2	Fruit	☐	☐	☐	☐	☐	☐	________
5.3	Egg	☐	☐	☐	☐	☐	☐	________
5.4	Dairy products including butter, cheeses, cream, yoghurt, custard and ice cream	☐	☐	☐	☐	☐	☐	________
5.5	Wheat containing products including breads, biscuits, rusks, cakes, breakfast cereals, pastries, pasta, couscous and dim sims	☐	☐	☐	☐	☐	☐	________
5.6	Rice and/or rice containing products including rice cereals (e.g. Farax), rice cakes, rice noodles and rice flour	☐	☐	☐	☐	☐	☐	________
5.7	Soy containing products including tofu, miso, soy sauce, soy flour and soy-based dairy substitutes	☐	☐	☐	☐	☐	☐	________
5.8	Corn and/or corn containing products including popcorn, polenta, corn flakes, corn tortillas and corn flour (including products thickened with e.g. gravy)	☐	☐	☐	☐	☐	☐	________

[B] Refer to "Footnote B: Instructions for Question 4" on Page 3 for further clarification about answer this question
[C] Refer to "Footnote C: Instructions for Question 5" on Page 3 for further clarification about answer this question

		Not at all	1-3 times per week	4-6 times per week	1-2 times per day	3-4 times per day	5 or more times per day	Average amount eaten per occasion
5.9	Oats or oat containing products including porridge, oatmeal, muesli, ANZAC biscuits, bran and oat flour	☐	☐	☐	☐	☐	☐	________
5.10	Barley or rye containing products including barley water, many minestrone-style soups and rye bread	☐	☐	☐	☐	☐	☐	________
5.11	Nuts including peanut butter	☐	☐	☐	☐	☐	☐	________
5.12	Foods containing pork, lamb, chicken, turkey, or other poultry	☐	☐	☐	☐	☐	☐	________
5.13	Foods containing beef and veal	☐	☐	☐	☐	☐	☐	________
5.14	Foods containing fish or shellfish	☐	☐	☐	☐	☐	☐	________

If your baby consumed any of the solid foods outlined above, please provide some additional information about what those foods were with respect to each category. This may include the **name of the food**, the **brand of the product** (where applicable) and whether the food was **modified** (for example: low-fat, added calcium, wholemeal etc.).[D] Please also list any foods your baby consumed that do not fall in the above categories.

5.15

[D] Refer to "Footnote D: Instructions for Question 5" on Page 5 for further clarification about answer this question

What type of methods we used to cook your baby's solid foods (tick all that applies)?

5.16 What type of methods we used to cook your baby's solid foods (tick all that applies)?

☐ Did not cook ☐ Baking ☐ Steaming

☐ Grilling ☐ Roasting ☐ Boiling

☐ Stewing ☐ Frying ☐ BBQ

☐ Microwaving ☐ Other___________________________________

6. The following questions relates to the use of dummies/pacifiers.

6.1 Do you regularly offer your baby a dummy/pacifier?

☐ Yes ☐ No

6.2 Did you use any sweeteners on the dummy/pacifier?

☐ No ☐ Yes, honey

☐ Yes, other ____

7. The following questions relate to occasion when your baby was given **supplements and medications**.[E] Please state the brand, what the volume of a "dose" was (e.g. 5 mL, 1 tablet, etc.), how many doses were given each day and on how many days it was given **this week**.

		Dose volume	Doses per day	Days
7.1	Supplements			
	☐ Multivitamin (brand:____________________)	_____	_____	_____
	☐ Omega-3/fish oil (brand:_________________)	_____	_____	_____
	☐ Iron (brand:___________________________)	_____	_____	_____
	☐ Other:_________________________________	_____	_____	_____
7.2	Antibiotics			
	Name:___________________________________	_____	_____	_____
	Name:___________________________________	_____	_____	_____
	Name:___________________________________	_____	_____	_____
7.3	Other as outlined in *Footnote E* (please specify)			
	Name:___________________________________	_____	_____	_____
	Name:___________________________________	_____	_____	_____
	Name:___________________________________	_____	_____	_____

[E] Refer to "Footnote E: Instructions for Question 7" on Page 6 for further clarification about answer this question

8. Please describe your child's health this week, specifying any **fevers** and/or illnesses that required you to visit a healthcare profession (e.g. child health nurse, doctor, naturopath, etc.)

9. Did your child receive any vaccinations this week, either scheduled or optional? If you child was not given the vaccination as per the National Immunisation Program (NIP) guidelines (for example, one particular vaccine was not given but others were) please provide details.

	Description	Timing	As per NIP guidelines	Variation of NIP guidelines (give details)
9.1	Scheduled	Birth	☐	
		2 months	☐	
		4 months	☐	
		6 months	☐	
		12 months	☐	
9.2	Optional	Influenza	☐	
		Pneumococcal 13vPCV	☐	
		Pneumococcal 23vPPV	☐	
		Hepatitis A	☐	
		Other:_________________	☐	

Your baby is 13 weeks old

Completion date: ____/____/____

1. In the past 7 days, how often was your baby feed each item listed below on average? Consider everyone that has feed the baby. Include snacks and night-time feeds.[A]

		Number feeds per day	Number of days given	Not given
1.1	Breast milk via breast feeding	_____	_____	☐
1.2	Expressed breast milk	_____	_____	☐
1.3	Formula	_____	_____	☐
1.4	Water	_____	_____	☐
1.5	Sugar water	_____	_____	☐
1.6	Cow's milk*	_____	_____	☐
1.7	Other milk**	_____	_____	☐
1.8	Fruit juices	_____	_____	☐
1.9	Other drinks‡	_____	_____	☐

* Includes plain, low-fat, evaporated and flavoured varieties of cow's milk. Include milk added to other foods such as cereal.
** Such as soy, rice, almond or goat milk. Include milk added to other foods such as cereal.
‡ Includes vegetable juice, soft drinks, cordial, sports drinks, yoghurt drinks, tea, coffee, etc.

2. The following questions relate to occasions when your baby consumed **breast milk**.

☐ *My baby did not consume breast milk this week (proceed to Question 3).*

2.1 Average feed duration when being breast feed:

_____________minutes

2.2 Average volume *consumed* when provided expressed breast milk:

_____________mL

☐ Never provided expressed breast milk *(proceed to Question 2.5)*

2.3 How long was expressed breast milk stored before it was fed to your baby (tick all that applies)?

☐ Kept at room temperature ☐ 1 day or less in refrigerator
☐ 2-3 days in refrigerator ☐ 3-4 days in refrigerator
☐ 5-7 days in refrigerator ☐ Frozen (for any length of time)

2.4 On the majority of occasions, how were bottles containing expressed breast milk warmed (tick only one)?

☐ Did not warm ☐ Hot water[Ψ]\bottle warmer
☐ Microwave ☐ Other____________________________

[A] Refer to "Footnote A: Instructions for Question 1" on Page 2 for further clarification about answer this question

2.5	Did you (the baby's mother) take any antibiotics this week? Please provide details about the type of antibiotic and dosage as described in Question 7.<br>☐ No ☐ Unsure<br>☐ Yes Type: ______________________________<br>Dose volume: ________________________<br>Doses per day: _______________________<br>Number of days: ______________________

ᵠ When bottle containing the milk is placed into a container of hot water

3. The following questions relate to occasions when your baby consumed **formula**.

   ☐ *My baby did not consume formula this week (proceed to Question 4).*

3.1	Average volume consumed with provided formula:<br>_____________mL
3.2	What were the brand(s) and product name(s) of the formula(s) your baby been given. For example, "Wyeth S-26 GOLD Newborn" or "Karicare Aptamil® Delact"<br>1.____<br>2.____<br>3.____<br>☐ Unknown
3.3	Was the formula prepared according to the manufacturer's instructions?<br>☐ Yes<br>☐ No (please provide details of how the formula was prepared)<br>____<br>____<br>____
3.5	On the majority of occasions, how were bottles containing formula warmed (tick only one)?<br>☐ Did not warm ☐ Prepared with hot water<br>☐ Hot waterᵠ\bottle warmer ☐ Microwave<br>☐ Other_____________________________

ᵠ When bottle containing the milk is placed into a container of hot water

4. The following question relates to occasions when your baby consumed **drinks other than breast milk, formula or plain water**. This includes cow's milk or milk from other non-dairy sources as well as fruit juices, vegetable juices, cordials, soft drinks, etc. Please state what the drink(s) were, the **brand of the product** (where applicable) and how much your baby consumed on occasions

these drinks were provided. Drink additives should also be included.[B] *If your baby has never received drinks other than breast milk, formula and water proceed to question 5.*

5. The following questions relate to occasions when your baby consumed **solid foods** this week. Please indicate which of the follow foods your baby received and, on each occasion your baby was offered that food, the average amount he/she consumed with respect to a "spoon" or "mound" code as indicated in the Instructions.[C]

		Not at all	1-3 times per week	4-6 times per week	1-2 times per day	3-4 times per day	5 or more times per day	Average amount eaten per occasion
5.1	Vegetables including potato and baked beans	☐	☐	☐	☐	☐	☐	________
5.2	Fruit	☐	☐	☐	☐	☐	☐	________
5.3	Egg	☐	☐	☐	☐	☐	☐	________
5.4	Dairy products including butter, cheeses, cream, yoghurt, custard and ice cream	☐	☐	☐	☐	☐	☐	________
5.5	Wheat containing products including breads, biscuits, rusks, cakes, breakfast cereals, pastries, pasta, couscous and dim sims	☐	☐	☐	☐	☐	☐	________
5.6	Rice and/or rice containing products including rice cereals (e.g. Farax), rice cakes, rice noodles and rice flour	☐	☐	☐	☐	☐	☐	________
5.7	Soy containing products including tofu, miso, soy sauce, soy flour and soy-based dairy substitutes	☐	☐	☐	☐	☐	☐	________
5.8	Corn and/or corn containing products including popcorn, polenta, corn flakes, corn tortillas and corn flour (including products thickened with e.g. gravy)	☐	☐	☐	☐	☐	☐	________

[B] Refer to "Footnote B: Instructions for Question 4" on Page 3 for further clarification about answer this question
[C] Refer to "Footnote C: Instructions for Question 5" on Page 3 for further clarification about answer this question

		Not at all	1-3 times per week	4-6 times per week	1-2 times per day	3-4 times per day	5 or more times per day	Average amount eaten per occasion
5.9	Oats or oat containing products including porridge, oatmeal, muesli, ANZAC biscuits, bran and oat flour	☐	☐	☐	☐	☐	☐	________
5.10	Barley or rye containing products including barley water, many minestrone-style soups and rye bread	☐	☐	☐	☐	☐	☐	________
5.11	Nuts including peanut butter	☐	☐	☐	☐	☐	☐	________
5.12	Foods containing pork, lamb, chicken, turkey, or other poultry	☐	☐	☐	☐	☐	☐	________
5.13	Foods containing beef and veal	☐	☐	☐	☐	☐	☐	________
5.14	Foods containing fish or shellfish	☐	☐	☐	☐	☐	☐	________

If your baby consumed any of the solid foods outlined above, please provide some additional information about what those foods were with respect to each category. This may include the **name of the food**, the **brand of the product** (where applicable) and whether the food was **modified** (for example: low-fat, added calcium, wholemeal etc.).[D] Please also list any foods your baby consumed that do not fall in the above categories.

5.15

[D] Refer to "Footnote D: Instructions for Question 5" on Page 5 for further clarification about answer this question

What type of methods we used to cook your baby's solid foods (tick all that applies)?

5.16	What type of methods we used to cook your baby's solid foods (tick all that applies)?<br>☐ Did not cook ☐ Baking ☐ Steaming<br>☐ Grilling ☐ Roasting ☐ Boiling<br>☐ Stewing ☐ Frying ☐ BBQ<br>☐ Microwaving ☐ Other___________________________________

6. The following questions relates to the use of dummies/pacifiers.

6.1	Do you regularly offer your baby a dummy/pacifier?<br>☐ Yes ☐ No
6.2	Did you use any sweeteners on the dummy/pacifier?<br>☐ No ☐ Yes, honey<br>☐ Yes, other ____

7. The following questions relate to occasion when your baby was given **supplements and medications**.[E] Please state the brand, what the volume of a "dose" was (e.g. 5 mL, 1 tablet, etc.), how many doses were given each day and on how many days it was given **this week**.

		Dose volume	Doses per day	Days
7.1	Supplements			
	☐ Multivitamin (brand:____________________)	_____	_____	_____
	☐ Omega-3/fish oil (brand:_________________)	_____	_____	_____
	☐ Iron (brand:____________________________)	_____	_____	_____
	☐ Other:_________________________________	_____	_____	_____
7.2	Antibiotics			
	Name:____________________________________	_____	_____	_____
	Name:____________________________________	_____	_____	_____
	Name:____________________________________	_____	_____	_____
7.3	Other as outlined in *Footnote E* (please specify)			
	Name:____________________________________	_____	_____	_____
	Name:____________________________________	_____	_____	_____
	Name:____________________________________	_____	_____	_____

[E] Refer to "Footnote E: Instructions for Question 7" on Page 6 for further clarification about answer this question

8. Please describe your child's health this week, specifying any **fevers** and/or illnesses that required you to visit a healthcare profession (e.g. child health nurse, doctor, naturopath, etc.)

9. Did your child receive any vaccinations this week, either scheduled or optional? If you child was not given the vaccination as per the National Immunisation Program (NIP) guidelines (for example, one particular vaccine was not given but others were) please provide details.

	Description	Timing	As per NIP guidelines	Variation of NIP guidelines (give details)
9.1	Scheduled	Birth	☐	
		2 months	☐	
		4 months	☐	
		6 months	☐	
		12 months	☐	
9.2	Optional	Influenza	☐	
		Pneumococcal 13vPCV	☐	
		Pneumococcal 23vPPV	☐	
		Hepatitis A	☐	
		Other:__________________	☐	

Your baby is 14 weeks old

Completion date: ____/____/____

1. In the past 7 days, how often was your baby feed each item listed below on average? Consider everyone that has feed the baby. Include snacks and night-time feeds.[A]

		Number feeds per day	Number of days given	Not given
1.1	Breast milk via breast feeding	_____	_____	☐
1.2	Expressed breast milk	_____	_____	☐
1.3	Formula	_____	_____	☐
1.4	Water	_____	_____	☐
1.5	Sugar water	_____	_____	☐
1.6	Cow's milk*	_____	_____	☐
1.7	Other milk**	_____	_____	☐
1.8	Fruit juices	_____	_____	☐
1.9	Other drinks[‡]	_____	_____	☐

* Includes plain, low-fat, evaporated and flavoured varieties of cow's milk. Include milk added to other foods such as cereal.

** Such as soy, rice, almond or goat milk. Include milk added to other foods such as cereal.

‡ Includes vegetable juice, soft drinks, cordial, sports drinks, yoghurt drinks, tea, coffee, etc.

2. The following questions relate to occasions when your baby consumed **breast milk**.

☐ *My baby did not consume breast milk this week (proceed to Question 3).*

2.1 Average feed duration when being breast feed:

_____________minutes

2.2 Average volume *consumed* when provided expressed breast milk:

_____________mL

☐ Never provided expressed breast milk *(proceed to Question 2.5)*

2.3 How long was expressed breast milk stored before it was fed to your baby (tick all that applies)?

☐ Kept at room temperature ☐ 1 day or less in refrigerator
☐ 2-3 days in refrigerator ☐ 3-4 days in refrigerator
☐ 5-7 days in refrigerator ☐ Frozen (for any length of time)

2.4 On the majority of occasions, how were bottles containing expressed breast milk warmed (tick only one)?

☐ Did not warm ☐ Hot water[Ψ]\bottle warmer
☐ Microwave ☐ Other_____________________________

[A] Refer to "Footnote A: Instructions for Question 1" on Page 2 for further clarification about answer this question

2.5	Did you (the baby's mother) take any antibiotics this week? Please provide details about the type of antibiotic and dosage as described in Question 7.<br>☐ No ☐ Unsure<br>☐ Yes Type: ______________________________<br>Dose volume: ________________________<br>Doses per day: _______________________<br>Number of days: ______________________

ᵠ When bottle containing the milk is placed into a container of hot water

3. The following questions relate to occasions when your baby consumed **formula**.
   ☐ *My baby did not consume formula this week (proceed to Question 4).*

3.1	Average volume consumed with provided formula:<br>_____________mL
3.2	What were the brand(s) and product name(s) of the formula(s) your baby been given. For example, "Wyeth S-26 GOLD Newborn" or "Karicare Aptamil® Delact"<br>1.____<br>2.____<br>3.____<br>☐ Unknown
3.3	Was the formula prepared according to the manufacturer's instructions?<br>☐ Yes<br>☐ No (please provide details of how the formula was prepared)<br>____<br>____<br>____
3.5	On the majority of occasions, how were bottles containing formula warmed (tick only one)?<br>☐ Did not warm ☐ Prepared with hot water<br>☐ Hot waterᵠ\bottle warmer ☐ Microwave<br>☐ Other____________________________

ᵠ When bottle containing the milk is placed into a container of hot water

4. The following question relates to occasions when your baby consumed **drinks other than breast milk, formula or plain water**. This includes cow's milk or milk from other non-dairy sources as well as fruit juices, vegetable juices, cordials, soft drinks, etc. Please state what the drink(s) were, the **brand of the product** (where applicable) and how much your baby consumed on occasions

these drinks were provided. Drink additives should also be included.[B] *If your baby has never received drinks other than breast milk, formula and water proceed to question 5.*

5. The following questions relate to occasions when your baby consumed **solid foods** this week. Please indicate which of the follow foods your baby received and, on each occasion your baby was offered that food, the average amount he/she consumed with respect to a "spoon" or "mound" code as indicated in the Instructions.[C]

		Not at all	1-3 times per week	4-6 times per week	1-2 times per day	3-4 times per day	5 or more times per day	Average amount eaten per occasion
5.1	Vegetables including potato and baked beans	☐	☐	☐	☐	☐	☐	________
5.2	Fruit	☐	☐	☐	☐	☐	☐	________
5.3	Egg	☐	☐	☐	☐	☐	☐	________
5.4	Dairy products including butter, cheeses, cream, yoghurt, custard and ice cream	☐	☐	☐	☐	☐	☐	________
5.5	Wheat containing products including breads, biscuits, rusks, cakes, breakfast cereals, pastries, pasta, couscous and dim sims	☐	☐	☐	☐	☐	☐	________
5.6	Rice and/or rice containing products including rice cereals (e.g. Farax), rice cakes, rice noodles and rice flour	☐	☐	☐	☐	☐	☐	________
5.7	Soy containing products including tofu, miso, soy sauce, soy flour and soy-based dairy substitutes	☐	☐	☐	☐	☐	☐	________
5.8	Corn and/or corn containing products including popcorn, polenta, corn flakes, corn tortillas and corn flour (including products thickened with e.g. gravy)	☐	☐	☐	☐	☐	☐	________

[B] Refer to "Footnote B: Instructions for Question 4" on Page 3 for further clarification about answer this question
[C] Refer to "Footnote C: Instructions for Question 5" on Page 3 for further clarification about answer this question

		Not at all	1-3 times per week	4-6 times per week	1-2 times per day	3-4 times per day	5 or more times per day	Average amount eaten per occasion
5.9	Oats or oat containing products including porridge, oatmeal, muesli, ANZAC biscuits, bran and oat flour	☐	☐	☐	☐	☐	☐	________
5.10	Barley or rye containing products including barley water, many minestrone-style soups and rye bread	☐	☐	☐	☐	☐	☐	________
5.11	Nuts including peanut butter	☐	☐	☐	☐	☐	☐	________
5.12	Foods containing pork, lamb, chicken, turkey, or other poultry	☐	☐	☐	☐	☐	☐	________
5.13	Foods containing beef and veal	☐	☐	☐	☐	☐	☐	________
5.14	Foods containing fish or shellfish	☐	☐	☐	☐	☐	☐	________

If your baby consumed any of the solid foods outlined above, please provide some additional information about what those foods were with respect to each category. This may include the **name of the food**, the **brand of the product** (where applicable) and whether the food was **modified** (for example: low-fat, added calcium, wholemeal etc.).[D] Please also list any foods your baby consumed that do not fall in the above categories.

5.15

[D] Refer to "Footnote D: Instructions for Question 5" on Page 5 for further clarification about answer this question

What type of methods we used to cook your baby's solid foods (tick all that applies)?

5.16	What type of methods we used to cook your baby's solid foods (tick all that applies)?<br>☐ Did not cook ☐ Baking ☐ Steaming<br>☐ Grilling ☐ Roasting ☐ Boiling<br>☐ Stewing ☐ Frying ☐ BBQ<br>☐ Microwaving ☐ Other___________________________________

6. The following questions relates to the use of dummies/pacifiers.

6.1	Do you regularly offer your baby a dummy/pacifier?<br>☐ Yes ☐ No
6.2	Did you use any sweeteners on the dummy/pacifier?<br>☐ No ☐ Yes, honey<br>☐ Yes, other ____

7. The following questions relate to occasion when your baby was given **supplements and medications**.[E] Please state the brand, what the volume of a "dose" was (e.g. 5 mL, 1 tablet, etc.), how many doses were given each day and on how many days it was given **this week**.

		Dose volume	Doses per day	Days
7.1	Supplements			
	☐ Multivitamin (brand:____________________)	_____	_____	_____
	☐ Omega-3/fish oil (brand:_________________)	_____	_____	_____
	☐ Iron (brand:____________________________)	_____	_____	_____
	☐ Other:_________________________________	_____	_____	_____
7.2	Antibiotics			
	Name:____________________________________	_____	_____	_____
	Name:____________________________________	_____	_____	_____
	Name:____________________________________	_____	_____	_____
7.3	Other as outlined in *Footnote E* (please specify)			
	Name:____________________________________	_____	_____	_____
	Name:____________________________________	_____	_____	_____
	Name:____________________________________	_____	_____	_____

[E] Refer to "Footnote E: Instructions for Question 7" on Page 6 for further clarification about answer this question

8. Please describe your child's health this week, specifying any **fevers** and/or illnesses that required you to visit a healthcare profession (e.g. child health nurse, doctor, naturopath, etc.)

9. Did your child receive any vaccinations this week, either scheduled or optional? If you child was not given the vaccination as per the National Immunisation Program (NIP) guidelines (for example, one particular vaccine was not given but others were) please provide details.

	Description	Timing	As per NIP guidelines	Variation of NIP guidelines (give details)
9.1	Scheduled	Birth	☐	
		2 months	☐	
		4 months	☐	
		6 months	☐	
		12 months	☐	
9.2	Optional	Influenza	☐	
		Pneumococcal 13vPCV	☐	
		Pneumococcal 23vPPV	☐	
		Hepatitis A	☐	
		Other:_________________	☐	

Your baby is 15 weeks old

Completion date: ____/____/____

1. In the past 7 days, how often was your baby feed each item listed below on average? Consider everyone that has feed the baby. Include snacks and night-time feeds.[A]

		Number feeds per day	Number of days given	Not given
1.1	Breast milk via breast feeding	_____	_____	☐
1.2	Expressed breast milk	_____	_____	☐
1.3	Formula	_____	_____	☐
1.4	Water	_____	_____	☐
1.5	Sugar water	_____	_____	☐
1.6	Cow's milk*	_____	_____	☐
1.7	Other milk**	_____	_____	☐
1.8	Fruit juices	_____	_____	☐
1.9	Other drinks‡	_____	_____	☐

* Includes plain, low-fat, evaporated and flavoured varieties of cow's milk. Include milk added to other foods such as cereal.

** Such as soy, rice, almond or goat milk. Include milk added to other foods such as cereal.

‡ Includes vegetable juice, soft drinks, cordial, sports drinks, yoghurt drinks, tea, coffee, etc.

2. The following questions relate to occasions when your baby consumed **breast milk**.

☐ *My baby did not consume breast milk this week (proceed to Question 3).*

2.1 Average feed duration when being breast feed:

_____________minutes

2.2 Average volume *consumed* when provided expressed breast milk:

_____________mL

☐ Never provided expressed breast milk *(proceed to Question 2.5)*

2.3 How long was expressed breast milk stored before it was fed to your baby (tick all that applies)?

☐ Kept at room temperature ☐ 1 day or less in refrigerator
☐ 2-3 days in refrigerator ☐ 3-4 days in refrigerator
☐ 5-7 days in refrigerator ☐ Frozen (for any length of time)

2.4 On the majority of occasions, how were bottles containing expressed breast milk warmed (tick only one)?

☐ Did not warm ☐ Hot water[Ψ]\bottle warmer
☐ Microwave ☐ Other_____________________________

[A] Refer to "Footnote A: Instructions for Question 1" on Page 2 for further clarification about answer this question

2.5	Did you (the baby's mother) take any antibiotics this week? Please provide details about the type of antibiotic and dosage as described in Question 7.<br>☐ No ☐ Unsure<br>☐ Yes Type: ______________________________<br>Dose volume: ________________________<br>Doses per day: _______________________<br>Number of days: ______________________

ᵠ When bottle containing the milk is placed into a container of hot water

3. The following questions relate to occasions when your baby consumed **formula**.
   ☐ *My baby did not consume formula this week (proceed to Question 4).*

3.1	Average volume consumed with provided formula:<br>_____________mL
3.2	What were the brand(s) and product name(s) of the formula(s) your baby been given. For example, "Wyeth S-26 GOLD Newborn" or "Karicare Aptamil® Delact"<br>1.____<br>2.____<br>3.____<br>☐ Unknown
3.3	Was the formula prepared according to the manufacturer's instructions?<br>☐ Yes<br>☐ No (please provide details of how the formula was prepared)<br>____<br>____<br>____
3.5	On the majority of occasions, how were bottles containing formula warmed (tick only one)?<br>☐ Did not warm ☐ Prepared with hot water<br>☐ Hot waterᵠ\bottle warmer ☐ Microwave<br>☐ Other____________________________

ᵠ When bottle containing the milk is placed into a container of hot water

4. The following question relates to occasions when your baby consumed **drinks other than breast milk, formula or plain water**. This includes cow's milk or milk from other non-dairy sources as well as fruit juices, vegetable juices, cordials, soft drinks, etc. Please state what the drink(s) were, the **brand of the product** (where applicable) and how much your baby consumed on occasions

these drinks were provided. Drink additives should also be included.[B] *If your baby has never received drinks other than breast milk, formula and water proceed to question 5.*

5. The following questions relate to occasions when your baby consumed **solid foods** this week. Please indicate which of the follow foods your baby received and, on each occasion your baby was offered that food, the average amount he/she consumed with respect to a "spoon" or "mound" code as indicated in the Instructions.[C]

		Not at all	1-3 times per week	4-6 times per week	1-2 times per day	3-4 times per day	5 or more times per day	Average amount eaten per occasion
5.1	Vegetables including potato and baked beans	☐	☐	☐	☐	☐	☐	________
5.2	Fruit	☐	☐	☐	☐	☐	☐	________
5.3	Egg	☐	☐	☐	☐	☐	☐	________
5.4	Dairy products including butter, cheeses, cream, yoghurt, custard and ice cream	☐	☐	☐	☐	☐	☐	________
5.5	Wheat containing products including breads, biscuits, rusks, cakes, breakfast cereals, pastries, pasta, couscous and dim sims	☐	☐	☐	☐	☐	☐	________
5.6	Rice and/or rice containing products including rice cereals (e.g. Farax), rice cakes, rice noodles and rice flour	☐	☐	☐	☐	☐	☐	________
5.7	Soy containing products including tofu, miso, soy sauce, soy flour and soy-based dairy substitutes	☐	☐	☐	☐	☐	☐	________
5.8	Corn and/or corn containing products including popcorn, polenta, corn flakes, corn tortillas and corn flour (including products thickened with e.g. gravy)	☐	☐	☐	☐	☐	☐	________

[B] Refer to "Footnote B: Instructions for Question 4" on Page 3 for further clarification about answer this question
[C] Refer to "Footnote C: Instructions for Question 5" on Page 3 for further clarification about answer this question

		Not at all	1-3 times per week	4-6 times per week	1-2 times per day	3-4 times per day	5 or more times per day	Average amount eaten per occasion
5.9	Oats or oat containing products including porridge, oatmeal, muesli, ANZAC biscuits, bran and oat flour	☐	☐	☐	☐	☐	☐	________
5.10	Barley or rye containing products including barley water, many minestrone-style soups and rye bread	☐	☐	☐	☐	☐	☐	________
5.11	Nuts including peanut butter	☐	☐	☐	☐	☐	☐	________
5.12	Foods containing pork, lamb, chicken, turkey, or other poultry	☐	☐	☐	☐	☐	☐	________
5.13	Foods containing beef and veal	☐	☐	☐	☐	☐	☐	________
5.14	Foods containing fish or shellfish	☐	☐	☐	☐	☐	☐	________

If your baby consumed any of the solid foods outlined above, please provide some additional information about what those foods were with respect to each category. This may include the **name of the food**, the **brand of the product** (where applicable) and whether the food was **modified** (for example: low-fat, added calcium, wholemeal etc.).[D] Please also list any foods your baby consumed that do not fall in the above categories.

5.15

[D] Refer to "Footnote D: Instructions for Question 5" on Page 5 for further clarification about answer this question

What type of methods we used to cook your baby's solid foods (tick all that applies)?

5.16	What type of methods we used to cook your baby's solid foods (tick all that applies)?		
	☐ Did not cook	☐ Baking	☐ Steaming
	☐ Grilling	☐ Roasting	☐ Boiling
	☐ Stewing	☐ Frying	☐ BBQ
	☐ Microwaving	☐ Other___________________________________	

6. The following questions relates to the use of dummies/pacifiers.

6.1	Do you regularly offer your baby a dummy/pacifier?	
	☐ Yes	☐ No
6.2	Did you use any sweeteners on the dummy/pacifier?	
	☐ No	☐ Yes, honey
	☐ Yes, other ____	

7. The following questions relate to occasion when your baby was given **supplements and medications**.[E] Please state the brand, what the volume of a "dose" was (e.g. 5 mL, 1 tablet, etc.), how many doses were given each day and on how many days it was given **this week**.

		Dose volume	Doses per day	Days
7.1	Supplements			
	☐ Multivitamin (brand:____________________)	_____	_____	_____
	☐ Omega-3/fish oil (brand:_________________)	_____	_____	_____
	☐ Iron (brand:____________________________)	_____	_____	_____
	☐ Other:_________________________________	_____	_____	_____
7.2	Antibiotics			
	Name:____________________________________	_____	_____	_____
	Name:____________________________________	_____	_____	_____
	Name:____________________________________	_____	_____	_____
7.3	Other as outlined in *Footnote E* (please specify)			
	Name:____________________________________	_____	_____	_____
	Name:____________________________________	_____	_____	_____
	Name:____________________________________	_____	_____	_____

[E] Refer to "Footnote E: Instructions for Question 7" on Page 6 for further clarification about answer this question

8. Please describe your child's health this week, specifying any **fevers** and/or illnesses that required you to visit a healthcare profession (e.g. child health nurse, doctor, naturopath, etc.)

9. Did your child receive any vaccinations this week, either scheduled or optional? If you child was not given the vaccination as per the National Immunisation Program (NIP) guidelines (for example, one particular vaccine was not given but others were) please provide details.

	Description	Timing	As per NIP guidelines	Variation of NIP guidelines (give details)
9.1	Scheduled	Birth	☐	
		2 months	☐	
		4 months	☐	
		6 months	☐	
		12 months	☐	
9.2	Optional	Influenza	☐	
		Pneumococcal 13vPCV	☐	
		Pneumococcal 23vPPV	☐	
		Hepatitis A	☐	
		Other:_________________	☐	

Your baby is 16 weeks old

Completion date: ____/____/____

1. In the past 7 days, how often was your baby feed each item listed below on average? Consider everyone that has feed the baby. Include snacks and night-time feeds.[A]

		Number feeds per day	Number of days given	Not given
1.1	Breast milk via breast feeding	_____	_____	☐
1.2	Expressed breast milk	_____	_____	☐
1.3	Formula	_____	_____	☐
1.4	Water	_____	_____	☐
1.5	Sugar water	_____	_____	☐
1.6	Cow's milk*	_____	_____	☐
1.7	Other milk**	_____	_____	☐
1.8	Fruit juices	_____	_____	☐
1.9	Other drinks‡	_____	_____	☐

* Includes plain, low-fat, evaporated and flavoured varieties of cow's milk. Include milk added to other foods such as cereal.
** Such as soy, rice, almond or goat milk. Include milk added to other foods such as cereal.
‡ Includes vegetable juice, soft drinks, cordial, sports drinks, yoghurt drinks, tea, coffee, etc.

2. The following questions relate to occasions when your baby consumed **breast milk**.

☐ *My baby did not consume breast milk this week (proceed to Question 3).*

2.1 Average feed duration when being breast feed:

_____________minutes

2.2 Average volume *consumed* when provided expressed breast milk:

_____________mL

☐ Never provided expressed breast milk *(proceed to Question 2.5)*

2.3 How long was expressed breast milk stored before it was fed to your baby (tick all that applies)?

☐ Kept at room temperature ☐ 1 day or less in refrigerator
☐ 2-3 days in refrigerator ☐ 3-4 days in refrigerator
☐ 5-7 days in refrigerator ☐ Frozen (for any length of time)

2.4 On the majority of occasions, how were bottles containing expressed breast milk warmed (tick only one)?

☐ Did not warm ☐ Hot water[Ψ]\bottle warmer
☐ Microwave ☐ Other_____________________________

[A] Refer to "Footnote A: Instructions for Question 1" on Page 2 for further clarification about answer this question

2.5	Did you (the baby's mother) take any antibiotics this week? Please provide details about the type of antibiotic and dosage as described in Question 7.<br>☐ No ☐ Unsure<br>☐ Yes Type: ____________________<br>Dose volume: ____________________<br>Doses per day: ____________________<br>Number of days: ____________________

ᴪ When bottle containing the milk is placed into a container of hot water

3. The following questions relate to occasions when your baby consumed **formula**.

☐ *My baby did not consume formula this week (proceed to Question 4).*

3.1	Average volume consumed with provided formula:<br>____________mL
3.2	What were the brand(s) and product name(s) of the formula(s) your baby been given. For example, "Wyeth S-26 GOLD Newborn" or "Karicare Aptamil® Delact"<br>1.____________________<br>2.____________________<br>3.____________________<br>☐ Unknown
3.3	Was the formula prepared according to the manufacturer's instructions?<br>☐ Yes<br>☐ No (please provide details of how the formula was prepared)<br>____________________<br>____________________<br>____________________
3.5	On the majority of occasions, how were bottles containing formula warmed (tick only one)?<br>☐ Did not warm ☐ Prepared with hot water<br>☐ Hot waterᴪ\bottle warmer ☐ Microwave<br>☐ Other____________________

ᴪ When bottle containing the milk is placed into a container of hot water

4. The following question relates to occasions when your baby consumed **drinks other than breast milk, formula or plain water**. This includes cow's milk or milk from other non-dairy sources as well as fruit juices, vegetable juices, cordials, soft drinks, etc. Please state what the drink(s) were, the **brand of the product** (where applicable) and how much your baby consumed on occasions

these drinks were provided. Drink additives should also be included.[B] *If your baby has never received drinks other than breast milk, formula and water proceed to question 5.*

5. The following questions relate to occasions when your baby consumed **solid foods** this week. Please indicate which of the follow foods your baby received and, on each occasion your baby was offered that food, the average amount he/she consumed with respect to a "spoon" or "mound" code as indicated in the Instructions.[C]

		Not at all	1-3 times per week	4-6 times per week	1-2 times per day	3-4 times per day	5 or more times per day	Average amount eaten per occasion
5.1	Vegetables including potato and baked beans	☐	☐	☐	☐	☐	☐	________
5.2	Fruit	☐	☐	☐	☐	☐	☐	________
5.3	Egg	☐	☐	☐	☐	☐	☐	________
5.4	Dairy products including butter, cheeses, cream, yoghurt, custard and ice cream	☐	☐	☐	☐	☐	☐	________
5.5	Wheat containing products including breads, biscuits, rusks, cakes, breakfast cereals, pastries, pasta, couscous and dim sims	☐	☐	☐	☐	☐	☐	________
5.6	Rice and/or rice containing products including rice cereals (e.g. Farax), rice cakes, rice noodles and rice flour	☐	☐	☐	☐	☐	☐	________
5.7	Soy containing products including tofu, miso, soy sauce, soy flour and soy-based dairy substitutes	☐	☐	☐	☐	☐	☐	________
5.8	Corn and/or corn containing products including popcorn, polenta, corn flakes, corn tortillas and corn flour (including products thickened with e.g. gravy)	☐	☐	☐	☐	☐	☐	________

[B] Refer to "Footnote B: Instructions for Question 4" on Page 3 for further clarification about answer this question
[C] Refer to "Footnote C: Instructions for Question 5" on Page 3 for further clarification about answer this question

		Not at all	1-3 times per week	4-6 times per week	1-2 times per day	3-4 times per day	5 or more times per day	Average amount eaten per occasion
5.9	Oats or oat containing products including porridge, oatmeal, muesli, ANZAC biscuits, bran and oat flour	☐	☐	☐	☐	☐	☐	________
5.10	Barley or rye containing products including barley water, many minestrone-style soups and rye bread	☐	☐	☐	☐	☐	☐	________
5.11	Nuts including peanut butter	☐	☐	☐	☐	☐	☐	________
5.12	Foods containing pork, lamb, chicken, turkey, or other poultry	☐	☐	☐	☐	☐	☐	________
5.13	Foods containing beef and veal	☐	☐	☐	☐	☐	☐	________
5.14	Foods containing fish or shellfish	☐	☐	☐	☐	☐	☐	________

If your baby consumed any of the solid foods outlined above, please provide some additional information about what those foods were with respect to each category. This may include the **name of the food**, the **brand of the product** (where applicable) and whether the food was **modified** (for example: low-fat, added calcium, wholemeal etc.).[D] Please also list any foods your baby consumed that do not fall in the above categories.

5.15

[D] Refer to "Footnote D: Instructions for Question 5" on Page 5 for further clarification about answer this question

What type of methods we used to cook your baby's solid foods (tick all that applies)?

5.16	What type of methods we used to cook your baby's solid foods (tick all that applies)?<br>☐ Did not cook ☐ Baking ☐ Steaming<br>☐ Grilling ☐ Roasting ☐ Boiling<br>☐ Stewing ☐ Frying ☐ BBQ<br>☐ Microwaving ☐ Other___________________________________

6. The following questions relates to the use of dummies/pacifiers.

6.1	Do you regularly offer your baby a dummy/pacifier?<br>☐ Yes ☐ No
6.2	Did you use any sweeteners on the dummy/pacifier?<br>☐ No ☐ Yes, honey<br>☐ Yes, other ____

7. The following questions relate to occasion when your baby was given **supplements and medications**.[E] Please state the brand, what the volume of a "dose" was (e.g. 5 mL, 1 tablet, etc.), how many doses were given each day and on how many days it was given **this week**.

		Dose volume	Doses per day	Days
7.1	Supplements			
	☐ Multivitamin (brand:____________________)	_____	_____	_____
	☐ Omega-3/fish oil (brand:_________________)	_____	_____	_____
	☐ Iron (brand:___________________________)	_____	_____	_____
	☐ Other:_________________________________	_____	_____	_____
7.2	Antibiotics			
	Name:____________________________________	_____	_____	_____
	Name:____________________________________	_____	_____	_____
	Name:____________________________________	_____	_____	_____
7.3	Other as outlined in *Footnote E* (please specify)			
	Name:____________________________________	_____	_____	_____
	Name:____________________________________	_____	_____	_____
	Name:____________________________________	_____	_____	_____

[E] Refer to "Footnote E: Instructions for Question 7" on Page 6 for further clarification about answer this question

8. Please describe your child's health this week, specifying any **fevers** and/or illnesses that required you to visit a healthcare profession (e.g. child health nurse, doctor, naturopath, etc.)

9. Did your child receive any vaccinations this week, either scheduled or optional? If you child was not given the vaccination as per the National Immunisation Program (NIP) guidelines (for example, one particular vaccine was not given but others were) please provide details.

	Description	Timing	As per NIP guidelines	Variation of NIP guidelines (give details)
9.1	Scheduled	Birth	☐	
		2 months	☐	
		4 months	☐	
		6 months	☐	
		12 months	☐	
9.2	Optional	Influenza	☐	
		Pneumococcal 13vPCV	☐	
		Pneumococcal 23vPPV	☐	
		Hepatitis A	☐	
		Other:_________________	☐	

Your baby is 17 weeks old

Completion date: ____/____/____

1. In the past 7 days, how often was your baby feed each item listed below on average? Consider everyone that has feed the baby. Include snacks and night-time feeds.[A]

		Number feeds per day	Number of days given	Not given
1.1	Breast milk via breast feeding	_____	_____	☐
1.2	Expressed breast milk	_____	_____	☐
1.3	Formula	_____	_____	☐
1.4	Water	_____	_____	☐
1.5	Sugar water	_____	_____	☐
1.6	Cow's milk*	_____	_____	☐
1.7	Other milk**	_____	_____	☐
1.8	Fruit juices	_____	_____	☐
1.9	Other drinks‡	_____	_____	☐

* Includes plain, low-fat, evaporated and flavoured varieties of cow's milk. Include milk added to other foods such as cereal.

** Such as soy, rice, almond or goat milk. Include milk added to other foods such as cereal.

‡ Includes vegetable juice, soft drinks, cordial, sports drinks, yoghurt drinks, tea, coffee, etc.

2. The following questions relate to occasions when your baby consumed **breast milk**.

☐ *My baby did not consume breast milk this week (proceed to Question 3).*

2.1 Average feed duration when being breast feed:

_____________minutes

2.2 Average volume *consumed* when provided expressed breast milk:

_____________mL

☐ Never provided expressed breast milk *(proceed to Question 2.5)*

2.3 How long was expressed breast milk stored before it was fed to your baby (tick all that applies)?

☐ Kept at room temperature ☐ 1 day or less in refrigerator
☐ 2-3 days in refrigerator ☐ 3-4 days in refrigerator
☐ 5-7 days in refrigerator ☐ Frozen (for any length of time)

2.4 On the majority of occasions, how were bottles containing expressed breast milk warmed (tick only one)?

☐ Did not warm ☐ Hot water[Ψ]\bottle warmer
☐ Microwave ☐ Other_____________________________

[A] Refer to "Footnote A: Instructions for Question 1" on Page 2 for further clarification about answer this question

2.5	Did you (the baby's mother) take any antibiotics this week? Please provide details about the type of antibiotic and dosage as described in Question 7.<br>☐ No ☐ Unsure<br>☐ Yes Type: ______________________________<br>Dose volume: ________________________<br>Doses per day: _______________________<br>Number of days: ______________________

$^{\psi}$ When bottle containing the milk is placed into a container of hot water

3. The following questions relate to occasions when your baby consumed **formula**.
   ☐ *My baby did not consume formula this week (proceed to Question 4).*

3.1	Average volume consumed with provided formula:<br>_____________mL
3.2	What were the brand(s) and product name(s) of the formula(s) your baby been given. For example, "Wyeth S-26 GOLD Newborn" or "Karicare Aptamil® Delact"<br>1.____<br>2.____<br>3.____<br>☐ Unknown
3.3	Was the formula prepared according to the manufacturer's instructions?<br>☐ Yes<br>☐ No (please provide details of how the formula was prepared)<br>____<br>____<br>____
3.5	On the majority of occasions, how were bottles containing formula warmed (tick only one)?<br>☐ Did not warm ☐ Prepared with hot water<br>☐ Hot water$^{\psi}$\bottle warmer ☐ Microwave<br>☐ Other_____________________________

$^{\psi}$ When bottle containing the milk is placed into a container of hot water

4. The following question relates to occasions when your baby consumed **drinks other than breast milk, formula or plain water**. This includes cow's milk or milk from other non-dairy sources as well as fruit juices, vegetable juices, cordials, soft drinks, etc. Please state what the drink(s) were, the **brand of the product** (where applicable) and how much your baby consumed on occasions

these drinks were provided. Drink additives should also be included.[B] *If your baby has never received drinks other than breast milk, formula and water proceed to question 5.*

5. The following questions relate to occasions when your baby consumed **solid foods** this week. Please indicate which of the follow foods your baby received and, on each occasion your baby was offered that food, the average amount he/she consumed with respect to a "spoon" or "mound" code as indicated in the Instructions.[C]

		Not at all	1-3 times per week	4-6 times per week	1-2 times per day	3-4 times per day	5 or more times per day	Average amount eaten per occasion
5.1	Vegetables including potato and baked beans	☐	☐	☐	☐	☐	☐	________
5.2	Fruit	☐	☐	☐	☐	☐	☐	________
5.3	Egg	☐	☐	☐	☐	☐	☐	________
5.4	Dairy products including butter, cheeses, cream, yoghurt, custard and ice cream	☐	☐	☐	☐	☐	☐	________
5.5	Wheat containing products including breads, biscuits, rusks, cakes, breakfast cereals, pastries, pasta, couscous and dim sims	☐	☐	☐	☐	☐	☐	________
5.6	Rice and/or rice containing products including rice cereals (e.g. Farax), rice cakes, rice noodles and rice flour	☐	☐	☐	☐	☐	☐	________
5.7	Soy containing products including tofu, miso, soy sauce, soy flour and soy-based dairy substitutes	☐	☐	☐	☐	☐	☐	________
5.8	Corn and/or corn containing products including popcorn, polenta, corn flakes, corn tortillas and corn flour (including products thickened with e.g. gravy)	☐	☐	☐	☐	☐	☐	________

[B] Refer to "Footnote B: Instructions for Question 4" on Page 3 for further clarification about answer this question
[C] Refer to "Footnote C: Instructions for Question 5" on Page 3 for further clarification about answer this question

		Not at all	1-3 times per week	4-6 times per week	1-2 times per day	3-4 times per day	5 or more times per day	Average amount eaten per occasion
5.9	Oats or oat containing products including porridge, oatmeal, muesli, ANZAC biscuits, bran and oat flour	☐	☐	☐	☐	☐	☐	________
5.10	Barley or rye containing products including barley water, many minestrone-style soups and rye bread	☐	☐	☐	☐	☐	☐	________
5.11	Nuts including peanut butter	☐	☐	☐	☐	☐	☐	________
5.12	Foods containing pork, lamb, chicken, turkey, or other poultry	☐	☐	☐	☐	☐	☐	________
5.13	Foods containing beef and veal	☐	☐	☐	☐	☐	☐	________
5.14	Foods containing fish or shellfish	☐	☐	☐	☐	☐	☐	________

If your baby consumed any of the solid foods outlined above, please provide some additional information about what those foods were with respect to each category. This may include the **name of the food**, the **brand of the product** (where applicable) and whether the food was **modified** (for example: low-fat, added calcium, wholemeal etc.).[D] Please also list any foods your baby consumed that do not fall in the above categories.

5.15

[D] Refer to "Footnote D: Instructions for Question 5" on Page 5 for further clarification about answer this question

What type of methods we used to cook your baby's solid foods (tick all that applies)?

5.16	What type of methods we used to cook your baby's solid foods (tick all that applies)?<br>☐ Did not cook ☐ Baking ☐ Steaming<br>☐ Grilling ☐ Roasting ☐ Boiling<br>☐ Stewing ☐ Frying ☐ BBQ<br>☐ Microwaving ☐ Other___________________________________

6. The following questions relates to the use of dummies/pacifiers.

6.1	Do you regularly offer your baby a dummy/pacifier?<br>☐ Yes ☐ No
6.2	Did you use any sweeteners on the dummy/pacifier?<br>☐ No ☐ Yes, honey<br>☐ Yes, other ____

7. The following questions relate to occasion when your baby was given **supplements and medications**.[E] Please state the brand, what the volume of a "dose" was (e.g. 5 mL, 1 tablet, etc.), how many doses were given each day and on how many days it was given **this week**.

		Dose volume	Doses per day	Days
7.1	Supplements			
	☐ Multivitamin (brand:____________________)	_____	_____	_____
	☐ Omega-3/fish oil (brand:_________________)	_____	_____	_____
	☐ Iron (brand:____________________________)	_____	_____	_____
	☐ Other:_________________________________	_____	_____	_____
7.2	Antibiotics			
	Name:____________________________________	_____	_____	_____
	Name:____________________________________	_____	_____	_____
	Name:____________________________________	_____	_____	_____
7.3	Other as outlined in *Footnote E* (please specify)			
	Name:____________________________________	_____	_____	_____
	Name:____________________________________	_____	_____	_____
	Name:____________________________________	_____	_____	_____

[E] Refer to "Footnote E: Instructions for Question 7" on Page 6 for further clarification about answer this question

8. Please describe your child's health this week, specifying any **fevers** and/or illnesses that required you to visit a healthcare profession (e.g. child health nurse, doctor, naturopath, etc.)

9. Did your child receive any vaccinations this week, either scheduled or optional? If you child was not given the vaccination as per the National Immunisation Program (NIP) guidelines (for example, one particular vaccine was not given but others were) please provide details.

	Description	Timing	As per NIP guidelines	Variation of NIP guidelines (give details)
9.1	Scheduled	Birth	☐	
		2 months	☐	
		4 months	☐	
		6 months	☐	
		12 months	☐	
9.2	Optional	Influenza	☐	
		Pneumococcal 13vPCV	☐	
		Pneumococcal 23vPPV	☐	
		Hepatitis A	☐	
		Other:__________________	☐	

Your baby is 18 weeks old

Completion date: ____/____/____

1. In the past 7 days, how often was your baby feed each item listed below on average? Consider everyone that has feed the baby. Include snacks and night-time feeds.[A]

		Number feeds per day	Number of days given	Not given
1.1	Breast milk via breast feeding	_____	_____	☐
1.2	Expressed breast milk	_____	_____	☐
1.3	Formula	_____	_____	☐
1.4	Water	_____	_____	☐
1.5	Sugar water	_____	_____	☐
1.6	Cow's milk*	_____	_____	☐
1.7	Other milk**	_____	_____	☐
1.8	Fruit juices	_____	_____	☐
1.9	Other drinks‡	_____	_____	☐

* Includes plain, low-fat, evaporated and flavoured varieties of cow's milk. Include milk added to other foods such as cereal.

** Such as soy, rice, almond or goat milk. Include milk added to other foods such as cereal.

‡ Includes vegetable juice, soft drinks, cordial, sports drinks, yoghurt drinks, tea, coffee, etc.

2. The following questions relate to occasions when your baby consumed **breast milk**.

☐ *My baby did not consume breast milk this week (proceed to Question 3).*

2.1 Average feed duration when being breast feed:

_____________minutes

2.2 Average volume *consumed* when provided expressed breast milk:

_____________mL

☐ Never provided expressed breast milk *(proceed to Question 2.5)*

2.3 How long was expressed breast milk stored before it was fed to your baby (tick all that applies)?

☐ Kept at room temperature ☐ 1 day or less in refrigerator

☐ 2-3 days in refrigerator ☐ 3-4 days in refrigerator

☐ 5-7 days in refrigerator ☐ Frozen (for any length of time)

2.4 On the majority of occasions, how were bottles containing expressed breast milk warmed (tick only one)?

☐ Did not warm ☐ Hot water[Ψ]\bottle warmer

☐ Microwave ☐ Other_____________________________

[A] Refer to "Footnote A: Instructions for Question 1" on Page 2 for further clarification about answer this question

2.5	Did you (the baby's mother) take any antibiotics this week? Please provide details about the type of antibiotic and dosage as described in Question 7.<br>☐ No ☐ Unsure<br>☐ Yes Type: ______________________________<br>Dose volume: ________________________<br>Doses per day: _______________________<br>Number of days: ______________________

ᴪ When bottle containing the milk is placed into a container of hot water

3. The following questions relate to occasions when your baby consumed **formula**.
   ☐ *My baby did not consume formula this week (proceed to Question 4).*

3.1	Average volume consumed with provided formula:<br>_____________mL
3.2	What were the brand(s) and product name(s) of the formula(s) your baby been given. For example, "Wyeth S-26 GOLD Newborn" or "Karicare Aptamil® Delact"<br>1.____<br>2.____<br>3.____<br>☐ Unknown
3.3	Was the formula prepared according to the manufacturer's instructions?<br>☐ Yes<br>☐ No (please provide details of how the formula was prepared)<br>____<br>____<br>____
3.5	On the majority of occasions, how were bottles containing formula warmed (tick only one)?<br>☐ Did not warm ☐ Prepared with hot water<br>☐ Hot waterᴪ\bottle warmer ☐ Microwave<br>☐ Other_____________________________

ᴪ When bottle containing the milk is placed into a container of hot water

4. The following question relates to occasions when your baby consumed **drinks other than breast milk, formula or plain water**. This includes cow's milk or milk from other non-dairy sources as well as fruit juices, vegetable juices, cordials, soft drinks, etc. Please state what the drink(s) were, the **brand of the product** (where applicable) and how much your baby consumed on occasions

these drinks were provided. Drink additives should also be included.[B] *If your baby has never received drinks other than breast milk, formula and water proceed to question 5.*

5. The following questions relate to occasions when your baby consumed **solid foods** this week. Please indicate which of the follow foods your baby received and, on each occasion your baby was offered that food, the average amount he/she consumed with respect to a "spoon" or "mound" code as indicated in the Instructions.[C]

		Not at all	1-3 times per week	4-6 times per week	1-2 times per day	3-4 times per day	5 or more times per day	Average amount eaten per occasion
5.1	Vegetables including potato and baked beans	☐	☐	☐	☐	☐	☐	________
5.2	Fruit	☐	☐	☐	☐	☐	☐	________
5.3	Egg	☐	☐	☐	☐	☐	☐	________
5.4	Dairy products including butter, cheeses, cream, yoghurt, custard and ice cream	☐	☐	☐	☐	☐	☐	________
5.5	Wheat containing products including breads, biscuits, rusks, cakes, breakfast cereals, pastries, pasta, couscous and dim sims	☐	☐	☐	☐	☐	☐	________
5.6	Rice and/or rice containing products including rice cereals (e.g. Farax), rice cakes, rice noodles and rice flour	☐	☐	☐	☐	☐	☐	________
5.7	Soy containing products including tofu, miso, soy sauce, soy flour and soy-based dairy substitutes	☐	☐	☐	☐	☐	☐	________
5.8	Corn and/or corn containing products including popcorn, polenta, corn flakes, corn tortillas and corn flour (including products thickened with e.g. gravy)	☐	☐	☐	☐	☐	☐	________

[B] Refer to "Footnote B: Instructions for Question 4" on Page 3 for further clarification about answer this question
[C] Refer to "Footnote C: Instructions for Question 5" on Page 3 for further clarification about answer this question

		Not at all	1-3 times per week	4-6 times per week	1-2 times per day	3-4 times per day	5 or more times per day	Average amount eaten per occasion
5.9	Oats or oat containing products including porridge, oatmeal, muesli, ANZAC biscuits, bran and oat flour	☐	☐	☐	☐	☐	☐	________
5.10	Barley or rye containing products including barley water, many minestrone-style soups and rye bread	☐	☐	☐	☐	☐	☐	________
5.11	Nuts including peanut butter	☐	☐	☐	☐	☐	☐	________
5.12	Foods containing pork, lamb, chicken, turkey, or other poultry	☐	☐	☐	☐	☐	☐	________
5.13	Foods containing beef and veal	☐	☐	☐	☐	☐	☐	________
5.14	Foods containing fish or shellfish	☐	☐	☐	☐	☐	☐	________

If your baby consumed any of the solid foods outlined above, please provide some additional information about what those foods were with respect to each category. This may include the **name of the food**, the **brand of the product** (where applicable) and whether the food was **modified** (for example: low-fat, added calcium, wholemeal etc.).[D] Please also list any foods your baby consumed that do not fall in the above categories.

5.15

[D] Refer to "Footnote D: Instructions for Question 5" on Page 5 for further clarification about answer this question

What type of methods we used to cook your baby's solid foods (tick all that applies)?

5.16	What type of methods we used to cook your baby's solid foods (tick all that applies)?<br>☐ Did not cook ☐ Baking ☐ Steaming<br>☐ Grilling ☐ Roasting ☐ Boiling<br>☐ Stewing ☐ Frying ☐ BBQ<br>☐ Microwaving ☐ Other___________________________________

6. The following questions relates to the use of dummies/pacifiers.

6.1	Do you regularly offer your baby a dummy/pacifier?<br>☐ Yes ☐ No
6.2	Did you use any sweeteners on the dummy/pacifier?<br>☐ No ☐ Yes, honey<br>☐ Yes, other ____

7. The following questions relate to occasion when your baby was given **supplements and medications**.[E] Please state the brand, what the volume of a "dose" was (e.g. 5 mL, 1 tablet, etc.), how many doses were given each day and on how many days it was given **this week**.

		Dose volume	Doses per day	Days
7.1	Supplements			
	☐ Multivitamin (brand:____________________)	_____	_____	_____
	☐ Omega-3/fish oil (brand:_________________)	_____	_____	_____
	☐ Iron (brand:____________________________)	_____	_____	_____
	☐ Other:_________________________________	_____	_____	_____
7.2	Antibiotics			
	Name:____________________________________	_____	_____	_____
	Name:____________________________________	_____	_____	_____
	Name:____________________________________	_____	_____	_____
7.3	Other as outlined in *Footnote E* (please specify)			
	Name:____________________________________	_____	_____	_____
	Name:____________________________________	_____	_____	_____
	Name:____________________________________	_____	_____	_____

[E] Refer to "Footnote E: Instructions for Question 7" on Page 6 for further clarification about answer this question

8. Please describe your child's health this week, specifying any **fevers** and/or illnesses that required you to visit a healthcare profession (e.g. child health nurse, doctor, naturopath, etc.)

9. Did your child receive any vaccinations this week, either scheduled or optional? If you child was not given the vaccination as per the National Immunisation Program (NIP) guidelines (for example, one particular vaccine was not given but others were) please provide details.

	Description	Timing	As per NIP guidelines	Variation of NIP guidelines (give details)
9.1	Scheduled	Birth	☐	
		2 months	☐	
		4 months	☐	
		6 months	☐	
		12 months	☐	
9.2	Optional	Influenza	☐	
		Pneumococcal 13vPCV	☐	
		Pneumococcal 23vPPV	☐	
		Hepatitis A	☐	
		Other:_________________	☐	

Your baby is 19 weeks old

Completion date: ____/____/____

1. In the past 7 days, how often was your baby feed each item listed below on average? Consider everyone that has feed the baby. Include snacks and night-time feeds.[A]

		Number feeds per day	Number of days given	Not given
1.1	Breast milk via breast feeding	_____	_____	☐
1.2	Expressed breast milk	_____	_____	☐
1.3	Formula	_____	_____	☐
1.4	Water	_____	_____	☐
1.5	Sugar water	_____	_____	☐
1.6	Cow's milk*	_____	_____	☐
1.7	Other milk**	_____	_____	☐
1.8	Fruit juices	_____	_____	☐
1.9	Other drinks‡	_____	_____	☐

* Includes plain, low-fat, evaporated and flavoured varieties of cow's milk. Include milk added to other foods such as cereal.
** Such as soy, rice, almond or goat milk. Include milk added to other foods such as cereal.
‡ Includes vegetable juice, soft drinks, cordial, sports drinks, yoghurt drinks, tea, coffee, etc.

2. The following questions relate to occasions when your baby consumed **breast milk**.

☐ *My baby did not consume breast milk this week (proceed to Question 3).*

2.1 Average feed duration when being breast feed:

_____________minutes

2.2 Average volume *consumed* when provided expressed breast milk:

_____________mL

☐ Never provided expressed breast milk *(proceed to Question 2.5)*

2.3 How long was expressed breast milk stored before it was fed to your baby (tick all that applies)?

☐ Kept at room temperature ☐ 1 day or less in refrigerator
☐ 2-3 days in refrigerator ☐ 3-4 days in refrigerator
☐ 5-7 days in refrigerator ☐ Frozen (for any length of time)

2.4 On the majority of occasions, how were bottles containing expressed breast milk warmed (tick only one)?

☐ Did not warm ☐ Hot water[Ψ]\bottle warmer
☐ Microwave ☐ Other_____________________________

[A] Refer to "Footnote A: Instructions for Question 1" on Page 2 for further clarification about answer this question

2.5	Did you (the baby's mother) take any antibiotics this week? Please provide details about the type of antibiotic and dosage as described in Question 7.<br>☐ No ☐ Unsure<br>☐ Yes Type: ______________________________<br>Dose volume: ________________________<br>Doses per day: _______________________<br>Number of days: ______________________

Ψ When bottle containing the milk is placed into a container of hot water

3. The following questions relate to occasions when your baby consumed **formula**.

   ☐ *My baby did not consume formula this week (proceed to Question 4).*

3.1	Average volume consumed with provided formula:<br>_____________mL
3.2	What were the brand(s) and product name(s) of the formula(s) your baby been given. For example, "Wyeth S-26 GOLD Newborn" or "Karicare Aptamil® Delact"<br>1.____<br>2.____<br>3.____<br>☐ Unknown
3.3	Was the formula prepared according to the manufacturer's instructions?<br>☐ Yes<br>☐ No (please provide details of how the formula was prepared)<br>____<br>____<br>____
3.5	On the majority of occasions, how were bottles containing formula warmed (tick only one)?<br>☐ Did not warm ☐ Prepared with hot water<br>☐ Hot waterΨ\bottle warmer ☐ Microwave<br>☐ Other_____________________________

Ψ When bottle containing the milk is placed into a container of hot water

4. The following question relates to occasions when your baby consumed **drinks other than breast milk, formula or plain water**. This includes cow's milk or milk from other non-dairy sources as well as fruit juices, vegetable juices, cordials, soft drinks, etc. Please state what the drink(s) were, the **brand of the product** (where applicable) and how much your baby consumed on occasions

these drinks were provided. Drink additives should also be included.[B] *If your baby has never received drinks other than breast milk, formula and water proceed to question 5.*

5. The following questions relate to occasions when your baby consumed **solid foods** this week. Please indicate which of the follow foods your baby received and, on each occasion your baby was offered that food, the average amount he/she consumed with respect to a "spoon" or "mound" code as indicated in the Instructions.[C]

		Not at all	1-3 times per week	4-6 times per week	1-2 times per day	3-4 times per day	5 or more times per day	Average amount eaten per occasion
5.1	Vegetables including potato and baked beans	☐	☐	☐	☐	☐	☐	________
5.2	Fruit	☐	☐	☐	☐	☐	☐	________
5.3	Egg	☐	☐	☐	☐	☐	☐	________
5.4	Dairy products including butter, cheeses, cream, yoghurt, custard and ice cream	☐	☐	☐	☐	☐	☐	________
5.5	Wheat containing products including breads, biscuits, rusks, cakes, breakfast cereals, pastries, pasta, couscous and dim sims	☐	☐	☐	☐	☐	☐	________
5.6	Rice and/or rice containing products including rice cereals (e.g. Farax), rice cakes, rice noodles and rice flour	☐	☐	☐	☐	☐	☐	________
5.7	Soy containing products including tofu, miso, soy sauce, soy flour and soy-based dairy substitutes	☐	☐	☐	☐	☐	☐	________
5.8	Corn and/or corn containing products including popcorn, polenta, corn flakes, corn tortillas and corn flour (including products thickened with e.g. gravy)	☐	☐	☐	☐	☐	☐	________

[B] Refer to "Footnote B: Instructions for Question 4" on Page 3 for further clarification about answer this question

[C] Refer to "Footnote C: Instructions for Question 5" on Page 3 for further clarification about answer this question

		Not at all	1-3 times per week	4-6 times per week	1-2 times per day	3-4 times per day	5 or more times per day	Average amount eaten per occasion
5.9	Oats or oat containing products including porridge, oatmeal, muesli, ANZAC biscuits, bran and oat flour	☐	☐	☐	☐	☐	☐	________
5.10	Barley or rye containing products including barley water, many minestrone-style soups and rye bread	☐	☐	☐	☐	☐	☐	________
5.11	Nuts including peanut butter	☐	☐	☐	☐	☐	☐	________
5.12	Foods containing pork, lamb, chicken, turkey, or other poultry	☐	☐	☐	☐	☐	☐	________
5.13	Foods containing beef and veal	☐	☐	☐	☐	☐	☐	________
5.14	Foods containing fish or shellfish	☐	☐	☐	☐	☐	☐	________

If your baby consumed any of the solid foods outlined above, please provide some additional information about what those foods were with respect to each category. This may include the **name of the food**, the **brand of the product** (where applicable) and whether the food was **modified** (for example: low-fat, added calcium, wholemeal etc.).[D] Please also list any foods your baby consumed that do not fall in the above categories.

5.15

[D] Refer to "Footnote D: Instructions for Question 5" on Page 5 for further clarification about answer this question

What type of methods we used to cook your baby's solid foods (tick all that applies)?

5.16	What type of methods we used to cook your baby's solid foods (tick all that applies)?			
		☐ Did not cook	☐ Baking	☐ Steaming
		☐ Grilling	☐ Roasting	☐ Boiling
		☐ Stewing	☐ Frying	☐ BBQ
		☐ Microwaving	☐ Other___________________________	

6. The following questions relates to the use of dummies/pacifiers.

6.1	Do you regularly offer your baby a dummy/pacifier?		
		☐ Yes	☐ No
6.2	Did you use any sweeteners on the dummy/pacifier?		
		☐ No	☐ Yes, honey
		☐ Yes, other ____	

7. The following questions relate to occasion when your baby was given **supplements and medications**.[E] Please state the brand, what the volume of a "dose" was (e.g. 5 mL, 1 tablet, etc.), how many doses were given each day and on how many days it was given **this week**.

		Dose volume	Doses per day	Days
7.1	Supplements			
	☐ Multivitamin (brand:____________________)	_____	_____	_____
	☐ Omega-3/fish oil (brand:________________)	_____	_____	_____
	☐ Iron (brand:___________________________)	_____	_____	_____
	☐ Other:________________________________	_____	_____	_____
7.2	Antibiotics			
	Name:___________________________________	_____	_____	_____
	Name:___________________________________	_____	_____	_____
	Name:___________________________________	_____	_____	_____
7.3	Other as outlined in *Footnote E* (please specify)			
	Name:___________________________________	_____	_____	_____
	Name:___________________________________	_____	_____	_____
	Name:___________________________________	_____	_____	_____

[E] Refer to "Footnote E: Instructions for Question 7" on Page 6 for further clarification about answer this question

8. Please describe your child's health this week, specifying any **fevers** and/or illnesses that required you to visit a healthcare profession (e.g. child health nurse, doctor, naturopath, etc.)

9. Did your child receive any vaccinations this week, either scheduled or optional? If you child was not given the vaccination as per the National Immunisation Program (NIP) guidelines (for example, one particular vaccine was not given but others were) please provide details.

	Description	Timing	As per NIP guidelines	Variation of NIP guidelines (give details)
9.1	Scheduled	Birth	☐	
		2 months	☐	
		4 months	☐	
		6 months	☐	
		12 months	☐	
9.2	Optional	Influenza	☐	
		Pneumococcal 13vPCV	☐	
		Pneumococcal 23vPPV	☐	
		Hepatitis A	☐	
		Other:_________________	☐	

Your baby is 20 weeks old

Completion date: ____/____/____

1. In the past 7 days, how often was your baby feed each item listed below on average? Consider everyone that has feed the baby. Include snacks and night-time feeds.[A]

		Number feeds per day	Number of days given	Not given
1.1	Breast milk via breast feeding	_____	_____	☐
1.2	Expressed breast milk	_____	_____	☐
1.3	Formula	_____	_____	☐
1.4	Water	_____	_____	☐
1.5	Sugar water	_____	_____	☐
1.6	Cow's milk*	_____	_____	☐
1.7	Other milk**	_____	_____	☐
1.8	Fruit juices	_____	_____	☐
1.9	Other drinks‡	_____	_____	☐

* Includes plain, low-fat, evaporated and flavoured varieties of cow's milk. Include milk added to other foods such as cereal.

** Such as soy, rice, almond or goat milk. Include milk added to other foods such as cereal.

‡ Includes vegetable juice, soft drinks, cordial, sports drinks, yoghurt drinks, tea, coffee, etc.

2. The following questions relate to occasions when your baby consumed **breast milk**.

☐ *My baby did not consume breast milk this week (proceed to Question 3).*

2.1 Average feed duration when being breast feed:

_____________minutes

2.2 Average volume *consumed* when provided expressed breast milk:

_____________mL

☐ Never provided expressed breast milk *(proceed to Question 2.5)*

2.3 How long was expressed breast milk stored before it was fed to your baby (tick all that applies)?

☐ Kept at room temperature ☐ 1 day or less in refrigerator

☐ 2-3 days in refrigerator ☐ 3-4 days in refrigerator

☐ 5-7 days in refrigerator ☐ Frozen (for any length of time)

2.4 On the majority of occasions, how were bottles containing expressed breast milk warmed (tick only one)?

☐ Did not warm ☐ Hot water[Ψ]\bottle warmer

☐ Microwave ☐ Other_____________________________

[A] Refer to "Footnote A: Instructions for Question 1" on Page 2 for further clarification about answer this question

2.5	Did you (the baby's mother) take any antibiotics this week? Please provide details about the type of antibiotic and dosage as described in Question 7.	
	☐ No ☐ Unsure	
	☐ Yes Type: ______________________________<br>Dose volume: ________________________<br>Doses per day: _______________________<br>Number of days: ______________________	

ᵠ When bottle containing the milk is placed into a container of hot water

3. The following questions relate to occasions when your baby consumed **formula**.

☐ *My baby did not consume formula this week (proceed to Question 4).*

3.1	Average volume consumed with provided formula:<br>_____________mL
3.2	What were the brand(s) and product name(s) of the formula(s) your baby been given. For example, "Wyeth S-26 GOLD Newborn" or "Karicare Aptamil® Delact"<br>1.____<br>2.____<br>3.____<br>☐ Unknown
3.3	Was the formula prepared according to the manufacturer's instructions?<br>☐ Yes<br>☐ No (please provide details of how the formula was prepared)<br>____<br>____<br>____
3.5	On the majority of occasions, how were bottles containing formula warmed (tick only one)?<br>☐ Did not warm ☐ Prepared with hot water<br>☐ Hot waterᵠ\bottle warmer ☐ Microwave<br>☐ Other_____________________________

ᵠ When bottle containing the milk is placed into a container of hot water

4. The following question relates to occasions when your baby consumed **drinks other than breast milk, formula or plain water**. This includes cow's milk or milk from other non-dairy sources as well as fruit juices, vegetable juices, cordials, soft drinks, etc. Please state what the drink(s) were, the **brand of the product** (where applicable) and how much your baby consumed on occasions

these drinks were provided. Drink additives should also be included.[B] *If your baby has never received drinks other than breast milk, formula and water proceed to question 5.*

5. The following questions relate to occasions when your baby consumed **solid foods** this week. Please indicate which of the follow foods your baby received and, on each occasion your baby was offered that food, the average amount he/she consumed with respect to a "spoon" or "mound" code as indicated in the Instructions.[C]

		Not at all	1-3 times per week	4-6 times per week	1-2 times per day	3-4 times per day	5 or more times per day	Average amount eaten per occasion
5.1	Vegetables including potato and baked beans	☐	☐	☐	☐	☐	☐	________
5.2	Fruit	☐	☐	☐	☐	☐	☐	________
5.3	Egg	☐	☐	☐	☐	☐	☐	________
5.4	Dairy products including butter, cheeses, cream, yoghurt, custard and ice cream	☐	☐	☐	☐	☐	☐	________
5.5	Wheat containing products including breads, biscuits, rusks, cakes, breakfast cereals, pastries, pasta, couscous and dim sims	☐	☐	☐	☐	☐	☐	________
5.6	Rice and/or rice containing products including rice cereals (e.g. Farax), rice cakes, rice noodles and rice flour	☐	☐	☐	☐	☐	☐	________
5.7	Soy containing products including tofu, miso, soy sauce, soy flour and soy-based dairy substitutes	☐	☐	☐	☐	☐	☐	________
5.8	Corn and/or corn containing products including popcorn, polenta, corn flakes, corn tortillas and corn flour (including products thickened with e.g. gravy)	☐	☐	☐	☐	☐	☐	________

[B] Refer to "Footnote B: Instructions for Question 4" on Page 3 for further clarification about answer this question
[C] Refer to "Footnote C: Instructions for Question 5" on Page 3 for further clarification about answer this question

		Not at all	1-3 times per week	4-6 times per week	1-2 times per day	3-4 times per day	5 or more times per day	Average amount eaten per occasion
5.9	Oats or oat containing products including porridge, oatmeal, muesli, ANZAC biscuits, bran and oat flour	☐	☐	☐	☐	☐	☐	________
5.10	Barley or rye containing products including barley water, many minestrone-style soups and rye bread	☐	☐	☐	☐	☐	☐	________
5.11	Nuts including peanut butter	☐	☐	☐	☐	☐	☐	________
5.12	Foods containing pork, lamb, chicken, turkey, or other poultry	☐	☐	☐	☐	☐	☐	________
5.13	Foods containing beef and veal	☐	☐	☐	☐	☐	☐	________
5.14	Foods containing fish or shellfish	☐	☐	☐	☐	☐	☐	________

If your baby consumed any of the solid foods outlined above, please provide some additional information about what those foods were with respect to each category. This may include the **name of the food**, the **brand of the product** (where applicable) and whether the food was **modified** (for example: low-fat, added calcium, wholemeal etc.).[D] Please also list any foods your baby consumed that do not fall in the above categories.

5.15

[D] Refer to "Footnote D: Instructions for Question 5" on Page 5 for further clarification about answer this question

What type of methods we used to cook your baby's solid foods (tick all that applies)?

5.16	What type of methods we used to cook your baby's solid foods (tick all that applies)?<br>☐ Did not cook ☐ Baking ☐ Steaming<br>☐ Grilling ☐ Roasting ☐ Boiling<br>☐ Stewing ☐ Frying ☐ BBQ<br>☐ Microwaving ☐ Other________________________________

6. The following questions relates to the use of dummies/pacifiers.

6.1	Do you regularly offer your baby a dummy/pacifier?<br>☐ Yes ☐ No
6.2	Did you use any sweeteners on the dummy/pacifier?<br>☐ No ☐ Yes, honey<br>☐ Yes, other ____

7. The following questions relate to occasion when your baby was given **supplements and medications**.[E] Please state the brand, what the volume of a "dose" was (e.g. 5 mL, 1 tablet, etc.), how many doses were given each day and on how many days it was given **this week**.

		Dose volume	Doses per day	Days
7.1	Supplements			
	☐ Multivitamin (brand:____________________)	_____	_____	_____
	☐ Omega-3/fish oil (brand:________________)	_____	_____	_____
	☐ Iron (brand:___________________________)	_____	_____	_____
	☐ Other:______________________________	_____	_____	_____
7.2	Antibiotics			
	Name:________________________________	_____	_____	_____
	Name:________________________________	_____	_____	_____
	Name:________________________________	_____	_____	_____
7.3	Other as outlined in *Footnote E* (please specify)			
	Name:________________________________	_____	_____	_____
	Name:________________________________	_____	_____	_____
	Name:________________________________	_____	_____	_____

[E] Refer to "Footnote E: Instructions for Question 7" on Page 6 for further clarification about answer this question

8. Please describe your child's health this week, specifying any **fevers** and/or illnesses that required you to visit a healthcare profession (e.g. child health nurse, doctor, naturopath, etc.)

9. Did your child receive any vaccinations this week, either scheduled or optional? If you child was not given the vaccination as per the National Immunisation Program (NIP) guidelines (for example, one particular vaccine was not given but others were) please provide details.

	Description	Timing	As per NIP guidelines	Variation of NIP guidelines (give details)
9.1	Scheduled	Birth	☐	
		2 months	☐	
		4 months	☐	
		6 months	☐	
		12 months	☐	
9.2	Optional	Influenza	☐	
		Pneumococcal 13vPCV	☐	
		Pneumococcal 23vPPV	☐	
		Hepatitis A	☐	
		Other:__________________	☐	

Your baby is 21 weeks old

Completion date: ____/____/____

1. In the past 7 days, how often was your baby feed each item listed below on average? Consider everyone that has feed the baby. Include snacks and night-time feeds.[A]

		Number feeds per day	Number of days given	Not given
1.1	Breast milk via breast feeding	_____	_____	☐
1.2	Expressed breast milk	_____	_____	☐
1.3	Formula	_____	_____	☐
1.4	Water	_____	_____	☐
1.5	Sugar water	_____	_____	☐
1.6	Cow's milk*	_____	_____	☐
1.7	Other milk**	_____	_____	☐
1.8	Fruit juices	_____	_____	☐
1.9	Other drinks[‡]	_____	_____	☐

* Includes plain, low-fat, evaporated and flavoured varieties of cow's milk. Include milk added to other foods such as cereal.

** Such as soy, rice, almond or goat milk. Include milk added to other foods such as cereal.

‡ Includes vegetable juice, soft drinks, cordial, sports drinks, yoghurt drinks, tea, coffee, etc.

2. The following questions relate to occasions when your baby consumed **breast milk**.

☐ *My baby did not consume breast milk this week (proceed to Question 3).*

2.1 Average feed duration when being breast feed:

_____________minutes

2.2 Average volume *consumed* when provided expressed breast milk:

_____________mL

☐ Never provided expressed breast milk *(proceed to Question 2.5)*

2.3 How long was expressed breast milk stored before it was fed to your baby (tick all that applies)?

☐ Kept at room temperature ☐ 1 day or less in refrigerator
☐ 2-3 days in refrigerator ☐ 3-4 days in refrigerator
☐ 5-7 days in refrigerator ☐ Frozen (for any length of time)

2.4 On the majority of occasions, how were bottles containing expressed breast milk warmed (tick only one)?

☐ Did not warm ☐ Hot water[Ψ]\bottle warmer
☐ Microwave ☐ Other_____________________________

[A] Refer to "Footnote A: Instructions for Question 1" on Page 2 for further clarification about answer this question

2.5	Did you (the baby's mother) take any antibiotics this week? Please provide details about the type of antibiotic and dosage as described in Question 7.<br>☐ No ☐ Unsure<br>☐ Yes Type: ______________________________<br>Dose volume: ________________________<br>Doses per day: _______________________<br>Number of days: ______________________

Ψ When bottle containing the milk is placed into a container of hot water

3. The following questions relate to occasions when your baby consumed **formula**.

☐ *My baby did not consume formula this week (proceed to Question 4).*

3.1	Average volume consumed with provided formula:<br>_____________mL
3.2	What were the brand(s) and product name(s) of the formula(s) your baby been given. For example, "Wyeth S-26 GOLD Newborn" or "Karicare Aptamil® Delact"<br>1.____<br>2.____<br>3.____<br>☐ Unknown
3.3	Was the formula prepared according to the manufacturer's instructions?<br>☐ Yes<br>☐ No (please provide details of how the formula was prepared)<br>____<br>____<br>____
3.5	On the majority of occasions, how were bottles containing formula warmed (tick only one)?<br>☐ Did not warm ☐ Prepared with hot water<br>☐ Hot water[Ψ]\bottle warmer ☐ Microwave<br>☐ Other____________________________

Ψ When bottle containing the milk is placed into a container of hot water

4. The following question relates to occasions when your baby consumed **drinks other than breast milk, formula or plain water**. This includes cow's milk or milk from other non-dairy sources as well as fruit juices, vegetable juices, cordials, soft drinks, etc. Please state what the drink(s) were, the **brand of the product** (where applicable) and how much your baby consumed on occasions

these drinks were provided. Drink additives should also be included.[B] *If your baby has never received drinks other than breast milk, formula and water proceed to question 5.*

5. The following questions relate to occasions when your baby consumed **solid foods** this week. Please indicate which of the follow foods your baby received and, on each occasion your baby was offered that food, the average amount he/she consumed with respect to a "spoon" or "mound" code as indicated in the Instructions.[C]

		Not at all	1-3 times per week	4-6 times per week	1-2 times per day	3-4 times per day	5 or more times per day	Average amount eaten per occasion
5.1	Vegetables including potato and baked beans	☐	☐	☐	☐	☐	☐	________
5.2	Fruit	☐	☐	☐	☐	☐	☐	________
5.3	Egg	☐	☐	☐	☐	☐	☐	________
5.4	Dairy products including butter, cheeses, cream, yoghurt, custard and ice cream	☐	☐	☐	☐	☐	☐	________
5.5	Wheat containing products including breads, biscuits, rusks, cakes, breakfast cereals, pastries, pasta, couscous and dim sims	☐	☐	☐	☐	☐	☐	________
5.6	Rice and/or rice containing products including rice cereals (e.g. Farax), rice cakes, rice noodles and rice flour	☐	☐	☐	☐	☐	☐	________
5.7	Soy containing products including tofu, miso, soy sauce, soy flour and soy-based dairy substitutes	☐	☐	☐	☐	☐	☐	________
5.8	Corn and/or corn containing products including popcorn, polenta, corn flakes, corn tortillas and corn flour (including products thickened with e.g. gravy)	☐	☐	☐	☐	☐	☐	________

[B] Refer to "Footnote B: Instructions for Question 4" on Page 3 for further clarification about answer this question
[C] Refer to "Footnote C: Instructions for Question 5" on Page 3 for further clarification about answer this question

		Not at all	1-3 times per week	4-6 times per week	1-2 times per day	3-4 times per day	5 or more times per day	Average amount eaten per occasion
5.9	Oats or oat containing products including porridge, oatmeal, muesli, ANZAC biscuits, bran and oat flour	☐	☐	☐	☐	☐	☐	________
5.10	Barley or rye containing products including barley water, many minestrone-style soups and rye bread	☐	☐	☐	☐	☐	☐	________
5.11	Nuts including peanut butter	☐	☐	☐	☐	☐	☐	________
5.12	Foods containing pork, lamb, chicken, turkey, or other poultry	☐	☐	☐	☐	☐	☐	________
5.13	Foods containing beef and veal	☐	☐	☐	☐	☐	☐	________
5.14	Foods containing fish or shellfish	☐	☐	☐	☐	☐	☐	________

If your baby consumed any of the solid foods outlined above, please provide some additional information about what those foods were with respect to each category. This may include the **name of the food**, the **brand of the product** (where applicable) and whether the food was **modified** (for example: low-fat, added calcium, wholemeal etc.).[D] Please also list any foods your baby consumed that do not fall in the above categories.

5.15

[D] Refer to "Footnote D: Instructions for Question 5" on Page 5 for further clarification about answer this question

What type of methods we used to cook your baby's solid foods (tick all that applies)?

5.16	What type of methods we used to cook your baby's solid foods (tick all that applies)?<br>☐ Did not cook ☐ Baking ☐ Steaming<br>☐ Grilling ☐ Roasting ☐ Boiling<br>☐ Stewing ☐ Frying ☐ BBQ<br>☐ Microwaving ☐ Other___________________________________

6. The following questions relates to the use of dummies/pacifiers.

6.1	Do you regularly offer your baby a dummy/pacifier?<br>☐ Yes ☐ No
6.2	Did you use any sweeteners on the dummy/pacifier?<br>☐ No ☐ Yes, honey<br>☐ Yes, other ____

7. The following questions relate to occasion when your baby was given **supplements and medications**.[E] Please state the brand, what the volume of a "dose" was (e.g. 5 mL, 1 tablet, etc.), how many doses were given each day and on how many days it was given **this week**.

		Dose volume	Doses per day	Days
7.1	Supplements			
	☐ Multivitamin (brand:____________________)	_____	_____	_____
	☐ Omega-3/fish oil (brand:_________________)	_____	_____	_____
	☐ Iron (brand:___________________________)	_____	_____	_____
	☐ Other:________________________________	_____	_____	_____
7.2	Antibiotics			
	Name:___________________________________	_____	_____	_____
	Name:___________________________________	_____	_____	_____
	Name:___________________________________	_____	_____	_____
7.3	Other as outlined in *Footnote E* (please specify)			
	Name:___________________________________	_____	_____	_____
	Name:___________________________________	_____	_____	_____
	Name:___________________________________	_____	_____	_____

[E] Refer to "Footnote E: Instructions for Question 7" on Page 6 for further clarification about answer this question

8. Please describe your child's health this week, specifying any **fevers** and/or illnesses that required you to visit a healthcare profession (e.g. child health nurse, doctor, naturopath, etc.)

9. Did your child receive any vaccinations this week, either scheduled or optional? If you child was not given the vaccination as per the National Immunisation Program (NIP) guidelines (for example, one particular vaccine was not given but others were) please provide details.

	Description	Timing	As per NIP guidelines	Variation of NIP guidelines (give details)
9.1	Scheduled	Birth	☐	
		2 months	☐	
		4 months	☐	
		6 months	☐	
		12 months	☐	
9.2	Optional	Influenza	☐	
		Pneumococcal 13vPCV	☐	
		Pneumococcal 23vPPV	☐	
		Hepatitis A	☐	
		Other:_________________	☐	

Your baby is 22 weeks old

Completion date: ____/____/____

1. In the past 7 days, how often was your baby feed each item listed below on average? Consider everyone that has feed the baby. Include snacks and night-time feeds.[A]

		Number feeds per day	Number of days given	Not given
1.1	Breast milk via breast feeding	_____	_____	☐
1.2	Expressed breast milk	_____	_____	☐
1.3	Formula	_____	_____	☐
1.4	Water	_____	_____	☐
1.5	Sugar water	_____	_____	☐
1.6	Cow's milk*	_____	_____	☐
1.7	Other milk**	_____	_____	☐
1.8	Fruit juices	_____	_____	☐
1.9	Other drinks‡	_____	_____	☐

* Includes plain, low-fat, evaporated and flavoured varieties of cow's milk. Include milk added to other foods such as cereal.

** Such as soy, rice, almond or goat milk. Include milk added to other foods such as cereal.

‡ Includes vegetable juice, soft drinks, cordial, sports drinks, yoghurt drinks, tea, coffee, etc.

2. The following questions relate to occasions when your baby consumed **breast milk**.

☐ *My baby did not consume breast milk this week (proceed to Question 3).*

2.1 Average feed duration when being breast feed:

_____________minutes

2.2 Average volume *consumed* when provided expressed breast milk:

_____________mL

☐ Never provided expressed breast milk *(proceed to Question 2.5)*

2.3 How long was expressed breast milk stored before it was fed to your baby (tick all that applies)?

☐ Kept at room temperature ☐ 1 day or less in refrigerator
☐ 2-3 days in refrigerator ☐ 3-4 days in refrigerator
☐ 5-7 days in refrigerator ☐ Frozen (for any length of time)

2.4 On the majority of occasions, how were bottles containing expressed breast milk warmed (tick only one)?

☐ Did not warm ☐ Hot water[Ψ]\bottle warmer
☐ Microwave ☐ Other____________________________

[A] Refer to "Footnote A: Instructions for Question 1" on Page 2 for further clarification about answer this question

2.5	Did you (the baby's mother) take any antibiotics this week? Please provide details about the type of antibiotic and dosage as described in Question 7.<br>☐ No ☐ Unsure<br>☐ Yes Type: ____________________________<br>Dose volume: ________________________<br>Doses per day: _______________________<br>Number of days: ______________________

$^{\Psi}$ When bottle containing the milk is placed into a container of hot water

3. The following questions relate to occasions when your baby consumed **formula**.
   ☐ *My baby did not consume formula this week (proceed to Question 4).*

3.1	Average volume consumed with provided formula:<br>____________mL
3.2	What were the brand(s) and product name(s) of the formula(s) your baby been given. For example, "Wyeth S-26 GOLD Newborn" or "Karicare Aptamil® Delact"<br>1.____<br>2.____<br>3.____<br>☐ Unknown
3.3	Was the formula prepared according to the manufacturer's instructions?<br>☐ Yes<br>☐ No (please provide details of how the formula was prepared)<br>____<br>____<br>____
3.5	On the majority of occasions, how were bottles containing formula warmed (tick only one)?<br>☐ Did not warm ☐ Prepared with hot water<br>☐ Hot water$^{\Psi}$\bottle warmer ☐ Microwave<br>☐ Other___________________________

$^{\Psi}$ When bottle containing the milk is placed into a container of hot water

4. The following question relates to occasions when your baby consumed **drinks other than breast milk, formula or plain water**. This includes cow's milk or milk from other non-dairy sources as well as fruit juices, vegetable juices, cordials, soft drinks, etc. Please state what the drink(s) were, the **brand of the product** (where applicable) and how much your baby consumed on occasions

these drinks were provided. Drink additives should also be included.[B] *If your baby has never received drinks other than breast milk, formula and water proceed to question 5.*

5. The following questions relate to occasions when your baby consumed **solid foods** this week. Please indicate which of the follow foods your baby received and, on each occasion your baby was offered that food, the average amount he/she consumed with respect to a "spoon" or "mound" code as indicated in the Instructions.[C]

		Not at all	1-3 times per week	4-6 times per week	1-2 times per day	3-4 times per day	5 or more times per day	Average amount eaten per occasion
5.1	Vegetables including potato and baked beans	☐	☐	☐	☐	☐	☐	________
5.2	Fruit	☐	☐	☐	☐	☐	☐	________
5.3	Egg	☐	☐	☐	☐	☐	☐	________
5.4	Dairy products including butter, cheeses, cream, yoghurt, custard and ice cream	☐	☐	☐	☐	☐	☐	________
5.5	Wheat containing products including breads, biscuits, rusks, cakes, breakfast cereals, pastries, pasta, couscous and dim sims	☐	☐	☐	☐	☐	☐	________
5.6	Rice and/or rice containing products including rice cereals (e.g. Farax), rice cakes, rice noodles and rice flour	☐	☐	☐	☐	☐	☐	________
5.7	Soy containing products including tofu, miso, soy sauce, soy flour and soy-based dairy substitutes	☐	☐	☐	☐	☐	☐	________
5.8	Corn and/or corn containing products including popcorn, polenta, corn flakes, corn tortillas and corn flour (including products thickened with e.g. gravy)	☐	☐	☐	☐	☐	☐	________

[B] Refer to "Footnote B: Instructions for Question 4" on Page 3 for further clarification about answer this question
[C] Refer to "Footnote C: Instructions for Question 5" on Page 3 for further clarification about answer this question

		Not at all	1-3 times per week	4-6 times per week	1-2 times per day	3-4 times per day	5 or more times per day	Average amount eaten per occasion
5.9	Oats or oat containing products including porridge, oatmeal, muesli, ANZAC biscuits, bran and oat flour	☐	☐	☐	☐	☐	☐	________
5.10	Barley or rye containing products including barley water, many minestrone-style soups and rye bread	☐	☐	☐	☐	☐	☐	________
5.11	Nuts including peanut butter	☐	☐	☐	☐	☐	☐	________
5.12	Foods containing pork, lamb, chicken, turkey, or other poultry	☐	☐	☐	☐	☐	☐	________
5.13	Foods containing beef and veal	☐	☐	☐	☐	☐	☐	________
5.14	Foods containing fish or shellfish	☐	☐	☐	☐	☐	☐	________

If your baby consumed any of the solid foods outlined above, please provide some additional information about what those foods were with respect to each category. This may include the **name of the food**, the **brand of the product** (where applicable) and whether the food was **modified** (for example: low-fat, added calcium, wholemeal etc.).[D] Please also list any foods your baby consumed that do not fall in the above categories.

5.15

[D] Refer to "Footnote D: Instructions for Question 5" on Page 5 for further clarification about answer this question

What type of methods we used to cook your baby's solid foods (tick all that applies)?

5.16	What type of methods we used to cook your baby's solid foods (tick all that applies)?<br>☐ Did not cook ☐ Baking ☐ Steaming<br>☐ Grilling ☐ Roasting ☐ Boiling<br>☐ Stewing ☐ Frying ☐ BBQ<br>☐ Microwaving ☐ Other___________________________________

6. The following questions relates to the use of dummies/pacifiers.

6.1	Do you regularly offer your baby a dummy/pacifier?<br>☐ Yes ☐ No
6.2	Did you use any sweeteners on the dummy/pacifier?<br>☐ No ☐ Yes, honey<br>☐ Yes, other ____

7. The following questions relate to occasion when your baby was given **supplements and medications**.[E] Please state the brand, what the volume of a "dose" was (e.g. 5 mL, 1 tablet, etc.), how many doses were given each day and on how many days it was given **this week**.

		Dose volume	Doses per day	Days
7.1	Supplements			
	☐ Multivitamin (brand:____________________)	_____	_____	_____
	☐ Omega-3/fish oil (brand:_________________)	_____	_____	_____
	☐ Iron (brand:____________________________)	_____	_____	_____
	☐ Other:_________________________________	_____	_____	_____
7.2	Antibiotics			
	Name:____________________________________	_____	_____	_____
	Name:____________________________________	_____	_____	_____
	Name:____________________________________	_____	_____	_____
7.3	Other as outlined in *Footnote E* (please specify)			
	Name:____________________________________	_____	_____	_____
	Name:____________________________________	_____	_____	_____
	Name:____________________________________	_____	_____	_____

[E] Refer to "Footnote E: Instructions for Question 7" on Page 6 for further clarification about answer this question

8. Please describe your child's health this week, specifying any **fevers** and/or illnesses that required you to visit a healthcare profession (e.g. child health nurse, doctor, naturopath, etc.)

9. Did your child receive any vaccinations this week, either scheduled or optional? If you child was not given the vaccination as per the National Immunisation Program (NIP) guidelines (for example, one particular vaccine was not given but others were) please provide details.

	Description	Timing	As per NIP guidelines	Variation of NIP guidelines (give details)
9.1	Scheduled	Birth	☐	
		2 months	☐	
		4 months	☐	
		6 months	☐	
		12 months	☐	
9.2	Optional	Influenza	☐	
		Pneumococcal 13vPCV	☐	
		Pneumococcal 23vPPV	☐	
		Hepatitis A	☐	
		Other:_________________	☐	

Your baby is 23 weeks old

Completion date: ____/____/____

1. In the past 7 days, how often was your baby feed each item listed below on average? Consider everyone that has feed the baby. Include snacks and night-time feeds.[A]

		Number feeds per day	Number of days given	Not given
1.1	Breast milk via breast feeding	_____	_____	☐
1.2	Expressed breast milk	_____	_____	☐
1.3	Formula	_____	_____	☐
1.4	Water	_____	_____	☐
1.5	Sugar water	_____	_____	☐
1.6	Cow's milk*	_____	_____	☐
1.7	Other milk**	_____	_____	☐
1.8	Fruit juices	_____	_____	☐
1.9	Other drinks[‡]	_____	_____	☐

* Includes plain, low-fat, evaporated and flavoured varieties of cow's milk. Include milk added to other foods such as cereal.

** Such as soy, rice, almond or goat milk. Include milk added to other foods such as cereal.

‡ Includes vegetable juice, soft drinks, cordial, sports drinks, yoghurt drinks, tea, coffee, etc.

2. The following questions relate to occasions when your baby consumed **breast milk**.

☐ *My baby did not consume breast milk this week (proceed to Question 3).*

2.1 Average feed duration when being breast feed:

_____________minutes

2.2 Average volume *consumed* when provided expressed breast milk:

_____________mL

☐ Never provided expressed breast milk *(proceed to Question 2.5)*

2.3 How long was expressed breast milk stored before it was fed to your baby (tick all that applies)?

☐ Kept at room temperature
☐ 1 day or less in refrigerator
☐ 2-3 days in refrigerator
☐ 3-4 days in refrigerator
☐ 5-7 days in refrigerator
☐ Frozen (for any length of time)

2.4 On the majority of occasions, how were bottles containing expressed breast milk warmed (tick only one)?

☐ Did not warm
☐ Hot water[Ψ]\bottle warmer
☐ Microwave
☐ Other____________________________

[A] Refer to "Footnote A: Instructions for Question 1" on Page 2 for further clarification about answer this question

2.5	Did you (the baby's mother) take any antibiotics this week? Please provide details about the type of antibiotic and dosage as described in Question 7.<br>☐ No ☐ Unsure<br>☐ Yes Type: ______________________________<br>Dose volume: ________________________<br>Doses per day: _______________________<br>Number of days: ______________________

Ψ When bottle containing the milk is placed into a container of hot water

3. The following questions relate to occasions when your baby consumed **formula**.

   ☐ *My baby did not consume formula this week (proceed to Question 4).*

3.1	Average volume consumed with provided formula:<br>_____________mL
3.2	What were the brand(s) and product name(s) of the formula(s) your baby been given. For example, "Wyeth S-26 GOLD Newborn" or "Karicare Aptamil® Delact"<br>1.____<br>2.____<br>3.____<br>☐ Unknown
3.3	Was the formula prepared according to the manufacturer's instructions?<br>☐ Yes<br>☐ No (please provide details of how the formula was prepared)<br>____<br>____<br>____
3.5	On the majority of occasions, how were bottles containing formula warmed (tick only one)?<br>☐ Did not warm ☐ Prepared with hot water<br>☐ Hot waterΨ\bottle warmer ☐ Microwave<br>☐ Other____________________________

Ψ When bottle containing the milk is placed into a container of hot water

4. The following question relates to occasions when your baby consumed **drinks other than breast milk, formula or plain water**. This includes cow's milk or milk from other non-dairy sources as well as fruit juices, vegetable juices, cordials, soft drinks, etc. Please state what the drink(s) were, the **brand of the product** (where applicable) and how much your baby consumed on occasions

these drinks were provided. Drink additives should also be included.[B] *If your baby has never received drinks other than breast milk, formula and water proceed to question 5.*

5. The following questions relate to occasions when your baby consumed **solid foods** this week. Please indicate which of the follow foods your baby received and, on each occasion your baby was offered that food, the average amount he/she consumed with respect to a "spoon" or "mound" code as indicated in the Instructions.[C]

		Not at all	1-3 times per week	4-6 times per week	1-2 times per day	3-4 times per day	5 or more times per day	Average amount eaten per occasion
5.1	Vegetables including potato and baked beans	☐	☐	☐	☐	☐	☐	________
5.2	Fruit	☐	☐	☐	☐	☐	☐	________
5.3	Egg	☐	☐	☐	☐	☐	☐	________
5.4	Dairy products including butter, cheeses, cream, yoghurt, custard and ice cream	☐	☐	☐	☐	☐	☐	________
5.5	Wheat containing products including breads, biscuits, rusks, cakes, breakfast cereals, pastries, pasta, couscous and dim sims	☐	☐	☐	☐	☐	☐	________
5.6	Rice and/or rice containing products including rice cereals (e.g. Farax), rice cakes, rice noodles and rice flour	☐	☐	☐	☐	☐	☐	________
5.7	Soy containing products including tofu, miso, soy sauce, soy flour and soy-based dairy substitutes	☐	☐	☐	☐	☐	☐	________
5.8	Corn and/or corn containing products including popcorn, polenta, corn flakes, corn tortillas and corn flour (including products thickened with e.g. gravy)	☐	☐	☐	☐	☐	☐	________

[B] Refer to "Footnote B: Instructions for Question 4" on Page 3 for further clarification about answer this question

[C] Refer to "Footnote C: Instructions for Question 5" on Page 3 for further clarification about answer this question

		Not at all	1-3 times per week	4-6 times per week	1-2 times per day	3-4 times per day	5 or more times per day	Average amount eaten per occasion
5.9	Oats or oat containing products including porridge, oatmeal, muesli, ANZAC biscuits, bran and oat flour	☐	☐	☐	☐	☐	☐	________
5.10	Barley or rye containing products including barley water, many minestrone-style soups and rye bread	☐	☐	☐	☐	☐	☐	________
5.11	Nuts including peanut butter	☐	☐	☐	☐	☐	☐	________
5.12	Foods containing pork, lamb, chicken, turkey, or other poultry	☐	☐	☐	☐	☐	☐	________
5.13	Foods containing beef and veal	☐	☐	☐	☐	☐	☐	________
5.14	Foods containing fish or shellfish	☐	☐	☐	☐	☐	☐	________

If your baby consumed any of the solid foods outlined above, please provide some additional information about what those foods were with respect to each category. This may include the **name of the food**, the **brand of the product** (where applicable) and whether the food was **modified** (for example: low-fat, added calcium, wholemeal etc.).[D] Please also list any foods your baby consumed that do not fall in the above categories.

5.15

[D] Refer to "Footnote D: Instructions for Question 5" on Page 5 for further clarification about answer this question

What type of methods we used to cook your baby's solid foods (tick all that applies)?

5.16	What type of methods we used to cook your baby's solid foods (tick all that applies)?			
		☐ Did not cook	☐ Baking	☐ Steaming
		☐ Grilling	☐ Roasting	☐ Boiling
		☐ Stewing	☐ Frying	☐ BBQ
		☐ Microwaving	☐ Other___________________________________	

6. The following questions relates to the use of dummies/pacifiers.

6.1	Do you regularly offer your baby a dummy/pacifier?		
		☐ Yes	☐ No
6.2	Did you use any sweeteners on the dummy/pacifier?		
		☐ No	☐ Yes, honey
		☐ Yes, other ____	

7. The following questions relate to occasion when your baby was given **supplements and medications**.[E] Please state the brand, what the volume of a "dose" was (e.g. 5 mL, 1 tablet, etc.), how many doses were given each day and on how many days it was given **this week**.

		Dose volume	Doses per day	Days
7.1	Supplements			
	☐ Multivitamin (brand:____________________)	_____	_____	_____
	☐ Omega-3/fish oil (brand:_________________)	_____	_____	_____
	☐ Iron (brand:___________________________)	_____	_____	_____
	☐ Other:_________________________________	_____	_____	_____
7.2	Antibiotics			
	Name:____________________________________	_____	_____	_____
	Name:____________________________________	_____	_____	_____
	Name:____________________________________	_____	_____	_____
7.3	Other as outlined in *Footnote E* (please specify)			
	Name:____________________________________	_____	_____	_____
	Name:____________________________________	_____	_____	_____
	Name:____________________________________	_____	_____	_____

[E] Refer to "Footnote E: Instructions for Question 7" on Page 6 for further clarification about answer this question

8. Please describe your child's health this week, specifying any **fevers** and/or illnesses that required you to visit a healthcare profession (e.g. child health nurse, doctor, naturopath, etc.)

9. Did your child receive any vaccinations this week, either scheduled or optional? If you child was not given the vaccination as per the National Immunisation Program (NIP) guidelines (for example, one particular vaccine was not given but others were) please provide details.

	Description	Timing	As per NIP guidelines	Variation of NIP guidelines (give details)
9.1	Scheduled	Birth	☐	
		2 months	☐	
		4 months	☐	
		6 months	☐	
		12 months	☐	
9.2	Optional	Influenza	☐	
		Pneumococcal 13vPCV	☐	
		Pneumococcal 23vPPV	☐	
		Hepatitis A	☐	
		Other:_________________	☐	

Your baby is 24 weeks old

Completion date: ____/____/____

1. In the past 7 days, how often was your baby feed each item listed below on average? Consider everyone that has feed the baby. Include snacks and night-time feeds.[A]

		Number feeds per day	Number of days given	Not given
1.1	Breast milk via breast feeding	_____	_____	☐
1.2	Expressed breast milk	_____	_____	☐
1.3	Formula	_____	_____	☐
1.4	Water	_____	_____	☐
1.5	Sugar water	_____	_____	☐
1.6	Cow's milk*	_____	_____	☐
1.7	Other milk**	_____	_____	☐
1.8	Fruit juices	_____	_____	☐
1.9	Other drinks‡	_____	_____	☐

* Includes plain, low-fat, evaporated and flavoured varieties of cow's milk. Include milk added to other foods such as cereal.
** Such as soy, rice, almond or goat milk. Include milk added to other foods such as cereal.
‡ Includes vegetable juice, soft drinks, cordial, sports drinks, yoghurt drinks, tea, coffee, etc.

2. The following questions relate to occasions when your baby consumed **breast milk**.

☐ *My baby did not consume breast milk this week (proceed to Question 3).*

2.1 Average feed duration when being breast feed:

_____________minutes

2.2 Average volume *consumed* when provided expressed breast milk:

_____________mL

☐ Never provided expressed breast milk *(proceed to Question 2.5)*

2.3 How long was expressed breast milk stored before it was fed to your baby (tick all that applies)?

☐ Kept at room temperature ☐ 1 day or less in refrigerator
☐ 2-3 days in refrigerator ☐ 3-4 days in refrigerator
☐ 5-7 days in refrigerator ☐ Frozen (for any length of time)

2.4 On the majority of occasions, how were bottles containing expressed breast milk warmed (tick only one)?

☐ Did not warm ☐ Hot water[Ψ]\bottle warmer
☐ Microwave ☐ Other_____________________________

[A] Refer to "Footnote A: Instructions for Question 1" on Page 2 for further clarification about answer this question

2.5	Did you (the baby's mother) take any antibiotics this week? Please provide details about the type of antibiotic and dosage as described in Question 7.<br>☐ No ☐ Unsure<br>☐ Yes Type: ______________________________<br>Dose volume: ________________________<br>Doses per day: _______________________<br>Number of days: ______________________

Ψ When bottle containing the milk is placed into a container of hot water

3. The following questions relate to occasions when your baby consumed **formula**.

   ☐ *My baby did not consume formula this week (proceed to Question 4).*

3.1	Average volume consumed with provided formula:<br>_____________mL
3.2	What were the brand(s) and product name(s) of the formula(s) your baby been given. For example, "Wyeth S-26 GOLD Newborn" or "Karicare Aptamil® Delact"<br>1.____<br>2.____<br>3.____<br>☐ Unknown
3.3	Was the formula prepared according to the manufacturer's instructions?<br>☐ Yes<br>☐ No (please provide details of how the formula was prepared)<br>____<br>____<br>____
3.5	On the majority of occasions, how were bottles containing formula warmed (tick only one)?<br>☐ Did not warm ☐ Prepared with hot water<br>☐ Hot water[Ψ]\bottle warmer ☐ Microwave<br>☐ Other_____________________________

Ψ When bottle containing the milk is placed into a container of hot water

4. The following question relates to occasions when your baby consumed **drinks other than breast milk, formula or plain water**. This includes cow's milk or milk from other non-dairy sources as well as fruit juices, vegetable juices, cordials, soft drinks, etc. Please state what the drink(s) were, the **brand of the product** (where applicable) and how much your baby consumed on occasions

these drinks were provided. Drink additives should also be included.[B] *If your baby has never received drinks other than breast milk, formula and water proceed to question 5.*

5. The following questions relate to occasions when your baby consumed **solid foods** this week. Please indicate which of the follow foods your baby received and, on each occasion your baby was offered that food, the average amount he/she consumed with respect to a "spoon" or "mound" code as indicated in the Instructions.[C]

		Not at all	1-3 times per week	4-6 times per week	1-2 times per day	3-4 times per day	5 or more times per day	Average amount eaten per occasion
5.1	Vegetables including potato and baked beans	☐	☐	☐	☐	☐	☐	________
5.2	Fruit	☐	☐	☐	☐	☐	☐	________
5.3	Egg	☐	☐	☐	☐	☐	☐	________
5.4	Dairy products including butter, cheeses, cream, yoghurt, custard and ice cream	☐	☐	☐	☐	☐	☐	________
5.5	Wheat containing products including breads, biscuits, rusks, cakes, breakfast cereals, pastries, pasta, couscous and dim sims	☐	☐	☐	☐	☐	☐	________
5.6	Rice and/or rice containing products including rice cereals (e.g. Farax), rice cakes, rice noodles and rice flour	☐	☐	☐	☐	☐	☐	________
5.7	Soy containing products including tofu, miso, soy sauce, soy flour and soy-based dairy substitutes	☐	☐	☐	☐	☐	☐	________
5.8	Corn and/or corn containing products including popcorn, polenta, corn flakes, corn tortillas and corn flour (including products thickened with e.g. gravy)	☐	☐	☐	☐	☐	☐	________

[B] Refer to "Footnote B: Instructions for Question 4" on Page 3 for further clarification about answer this question
[C] Refer to "Footnote C: Instructions for Question 5" on Page 3 for further clarification about answer this question

		Not at all	1-3 times per week	4-6 times per week	1-2 times per day	3-4 times per day	5 or more times per day	Average amount eaten per occasion
5.9	Oats or oat containing products including porridge, oatmeal, muesli, ANZAC biscuits, bran and oat flour	☐	☐	☐	☐	☐	☐	________
5.10	Barley or rye containing products including barley water, many minestrone-style soups and rye bread	☐	☐	☐	☐	☐	☐	________
5.11	Nuts including peanut butter	☐	☐	☐	☐	☐	☐	________
5.12	Foods containing pork, lamb, chicken, turkey, or other poultry	☐	☐	☐	☐	☐	☐	________
5.13	Foods containing beef and veal	☐	☐	☐	☐	☐	☐	________
5.14	Foods containing fish or shellfish	☐	☐	☐	☐	☐	☐	________

If your baby consumed any of the solid foods outlined above, please provide some additional information about what those foods were with respect to each category. This may include the **name of the food**, the **brand of the product** (where applicable) and whether the food was **modified** (for example: low-fat, added calcium, wholemeal etc.).[D] Please also list any foods your baby consumed that do not fall in the above categories.

5.15

[D] Refer to "Footnote D: Instructions for Question 5" on Page 5 for further clarification about answer this question

What type of methods we used to cook your baby's solid foods (tick all that applies)?

5.16	What type of methods we used to cook your baby's solid foods (tick all that applies)?<br>☐ Did not cook ☐ Baking ☐ Steaming<br>☐ Grilling ☐ Roasting ☐ Boiling<br>☐ Stewing ☐ Frying ☐ BBQ<br>☐ Microwaving ☐ Other___________________________________

6. The following questions relates to the use of dummies/pacifiers.

6.1	Do you regularly offer your baby a dummy/pacifier?<br>☐ Yes ☐ No
6.2	Did you use any sweeteners on the dummy/pacifier?<br>☐ No ☐ Yes, honey<br>☐ Yes, other ____

7. The following questions relate to occasion when your baby was given **supplements and medications**.[E] Please state the brand, what the volume of a "dose" was (e.g. 5 mL, 1 tablet, etc.), how many doses were given each day and on how many days it was given **this week**.

		Dose volume	Doses per day	Days
7.1	Supplements			
	☐ Multivitamin (brand:____________________)	_____	_____	_____
	☐ Omega-3/fish oil (brand:_________________)	_____	_____	_____
	☐ Iron (brand:___________________________)	_____	_____	_____
	☐ Other:_________________________________	_____	_____	_____
7.2	Antibiotics			
	Name:____________________________________	_____	_____	_____
	Name:____________________________________	_____	_____	_____
	Name:____________________________________	_____	_____	_____
7.3	Other as outlined in *Footnote E* (please specify)			
	Name:____________________________________	_____	_____	_____
	Name:____________________________________	_____	_____	_____
	Name:____________________________________	_____	_____	_____

[E] Refer to "Footnote E: Instructions for Question 7" on Page 6 for further clarification about answer this question

8. Please describe your child's health this week, specifying any **fevers** and/or illnesses that required you to visit a healthcare profession (e.g. child health nurse, doctor, naturopath, etc.)

9. Did your child receive any vaccinations this week, either scheduled or optional? If you child was not given the vaccination as per the National Immunisation Program (NIP) guidelines (for example, one particular vaccine was not given but others were) please provide details.

	Description	Timing	As per NIP guidelines	Variation of NIP guidelines (give details)
9.1	Scheduled	Birth	☐	
		2 months	☐	
		4 months	☐	
		6 months	☐	
		12 months	☐	
9.2	Optional	Influenza	☐	
		Pneumococcal 13vPCV	☐	
		Pneumococcal 23vPPV	☐	
		Hepatitis A	☐	
		Other:_________________	☐	

Your baby is 25 weeks old

Completion date: ____/____/____

1. In the past 7 days, how often was your baby feed each item listed below on average? Consider everyone that has feed the baby. Include snacks and night-time feeds.[A]

		Number feeds per day	Number of days given	Not given
1.1	Breast milk via breast feeding	_____	_____	☐
1.2	Expressed breast milk	_____	_____	☐
1.3	Formula	_____	_____	☐
1.4	Water	_____	_____	☐
1.5	Sugar water	_____	_____	☐
1.6	Cow's milk*	_____	_____	☐
1.7	Other milk**	_____	_____	☐
1.8	Fruit juices	_____	_____	☐
1.9	Other drinks‡	_____	_____	☐

* Includes plain, low-fat, evaporated and flavoured varieties of cow's milk. Include milk added to other foods such as cereal.

** Such as soy, rice, almond or goat milk. Include milk added to other foods such as cereal.

‡ Includes vegetable juice, soft drinks, cordial, sports drinks, yoghurt drinks, tea, coffee, etc.

2. The following questions relate to occasions when your baby consumed **breast milk**.

☐ *My baby did not consume breast milk this week (proceed to Question 3).*

2.1 Average feed duration when being breast feed:

_____________minutes

2.2 Average volume *consumed* when provided expressed breast milk:

_____________mL

☐ Never provided expressed breast milk *(proceed to Question 2.5)*

2.3 How long was expressed breast milk stored before it was fed to your baby (tick all that applies)?

☐ Kept at room temperature ☐ 1 day or less in refrigerator

☐ 2-3 days in refrigerator ☐ 3-4 days in refrigerator

☐ 5-7 days in refrigerator ☐ Frozen (for any length of time)

2.4 On the majority of occasions, how were bottles containing expressed breast milk warmed (tick only one)?

☐ Did not warm ☐ Hot water[Ψ]\bottle warmer

☐ Microwave ☐ Other____________________________

[A] Refer to "Footnote A: Instructions for Question 1" on Page 2 for further clarification about answer this question

2.5	Did you (the baby's mother) take any antibiotics this week? Please provide details about the type of antibiotic and dosage as described in Question 7.<br>☐ No ☐ Unsure<br>☐ Yes Type: ______________<br>Dose volume: ______________<br>Doses per day: ______________<br>Number of days: ______________	

ᴪ When bottle containing the milk is placed into a container of hot water

3. The following questions relate to occasions when your baby consumed **formula**.

☐ *My baby did not consume formula this week (proceed to Question 4).*

3.1	Average volume consumed with provided formula:<br>______________mL	
3.2	What were the brand(s) and product name(s) of the formula(s) your baby been given. For example, "Wyeth S-26 GOLD Newborn" or "Karicare Aptamil® Delact"<br>1.______________<br>2.______________<br>3.______________<br>☐ Unknown	
3.3	Was the formula prepared according to the manufacturer's instructions?<br>☐ Yes<br>☐ No (please provide details of how the formula was prepared)<br>______________<br>______________<br>______________	
3.5	On the majority of occasions, how were bottles containing formula warmed (tick only one)?<br>☐ Did not warm ☐ Prepared with hot water<br>☐ Hot waterᴪ\bottle warmer ☐ Microwave<br>☐ Other______________	

ᴪ When bottle containing the milk is placed into a container of hot water

4. The following question relates to occasions when your baby consumed **drinks other than breast milk, formula or plain water**. This includes cow's milk or milk from other non-dairy sources as well as fruit juices, vegetable juices, cordials, soft drinks, etc. Please state what the drink(s) were, the **brand of the product** (where applicable) and how much your baby consumed on occasions

these drinks were provided. Drink additives should also be included.[B] *If your baby has never received drinks other than breast milk, formula and water proceed to question 5.*

5. The following questions relate to occasions when your baby consumed **solid foods** this week. Please indicate which of the follow foods your baby received and, on each occasion your baby was offered that food, the average amount he/she consumed with respect to a "spoon" or "mound" code as indicated in the Instructions.[C]

		Not at all	1-3 times per week	4-6 times per week	1-2 times per day	3-4 times per day	5 or more times per day	Average amount eaten per occasion
5.1	Vegetables including potato and baked beans	☐	☐	☐	☐	☐	☐	________
5.2	Fruit	☐	☐	☐	☐	☐	☐	________
5.3	Egg	☐	☐	☐	☐	☐	☐	________
5.4	Dairy products including butter, cheeses, cream, yoghurt, custard and ice cream	☐	☐	☐	☐	☐	☐	________
5.5	Wheat containing products including breads, biscuits, rusks, cakes, breakfast cereals, pastries, pasta, couscous and dim sims	☐	☐	☐	☐	☐	☐	________
5.6	Rice and/or rice containing products including rice cereals (e.g. Farax), rice cakes, rice noodles and rice flour	☐	☐	☐	☐	☐	☐	________
5.7	Soy containing products including tofu, miso, soy sauce, soy flour and soy-based dairy substitutes	☐	☐	☐	☐	☐	☐	________
5.8	Corn and/or corn containing products including popcorn, polenta, corn flakes, corn tortillas and corn flour (including products thickened with e.g. gravy)	☐	☐	☐	☐	☐	☐	________

[B] Refer to "Footnote B: Instructions for Question 4" on Page 3 for further clarification about answer this question
[C] Refer to "Footnote C: Instructions for Question 5" on Page 3 for further clarification about answer this question

		Not at all	1-3 times per week	4-6 times per week	1-2 times per day	3-4 times per day	5 or more times per day	Average amount eaten per occasion
5.9	Oats or oat containing products including porridge, oatmeal, muesli, ANZAC biscuits, bran and oat flour	☐	☐	☐	☐	☐	☐	________
5.10	Barley or rye containing products including barley water, many minestrone-style soups and rye bread	☐	☐	☐	☐	☐	☐	________
5.11	Nuts including peanut butter	☐	☐	☐	☐	☐	☐	________
5.12	Foods containing pork, lamb, chicken, turkey, or other poultry	☐	☐	☐	☐	☐	☐	________
5.13	Foods containing beef and veal	☐	☐	☐	☐	☐	☐	________
5.14	Foods containing fish or shellfish	☐	☐	☐	☐	☐	☐	________

If your baby consumed any of the solid foods outlined above, please provide some additional information about what those foods were with respect to each category. This may include the **name of the food**, the **brand of the product** (where applicable) and whether the food was **modified** (for example: low-fat, added calcium, wholemeal etc.).[D] Please also list any foods your baby consumed that do not fall in the above categories.

5.15

[D] Refer to “Footnote D: Instructions for Question 5” on Page 5 for further clarification about answer this question

What type of methods we used to cook your baby's solid foods (tick all that applies)?

5.16	What type of methods we used to cook your baby's solid foods (tick all that applies)?<br>☐ Did not cook ☐ Baking ☐ Steaming<br>☐ Grilling ☐ Roasting ☐ Boiling<br>☐ Stewing ☐ Frying ☐ BBQ<br>☐ Microwaving ☐ Other___________________________________

6. The following questions relates to the use of dummies/pacifiers.

6.1	Do you regularly offer your baby a dummy/pacifier?<br>☐ Yes ☐ No
6.2	Did you use any sweeteners on the dummy/pacifier?<br>☐ No ☐ Yes, honey<br>☐ Yes, other ____

7. The following questions relate to occasion when your baby was given **supplements and medications**.[E] Please state the brand, what the volume of a "dose" was (e.g. 5 mL, 1 tablet, etc.), how many doses were given each day and on how many days it was given **this week**.

		Dose volume	Doses per day	Days
7.1	Supplements			
	☐ Multivitamin (brand:____________________)	_____	_____	_____
	☐ Omega-3/fish oil (brand:_________________)	_____	_____	_____
	☐ Iron (brand:____________________________)	_____	_____	_____
	☐ Other:_________________________________	_____	_____	_____
7.2	Antibiotics			
	Name:____________________________________	_____	_____	_____
	Name:____________________________________	_____	_____	_____
	Name:____________________________________	_____	_____	_____
7.3	Other as outlined in *Footnote E* (please specify)			
	Name:____________________________________	_____	_____	_____
	Name:____________________________________	_____	_____	_____
	Name:____________________________________	_____	_____	_____

[E] Refer to "Footnote E: Instructions for Question 7" on Page 6 for further clarification about answer this question

8. Please describe your child's health this week, specifying any **fevers** and/or illnesses that required you to visit a healthcare profession (e.g. child health nurse, doctor, naturopath, etc.)

9. Did your child receive any vaccinations this week, either scheduled or optional? If you child was not given the vaccination as per the National Immunisation Program (NIP) guidelines (for example, one particular vaccine was not given but others were) please provide details.

	Description	Timing	As per NIP guidelines	Variation of NIP guidelines (give details)
9.1	Scheduled	Birth	☐	
		2 months	☐	
		4 months	☐	
		6 months	☐	
		12 months	☐	
9.2	Optional	Influenza	☐	
		Pneumococcal 13vPCV	☐	
		Pneumococcal 23vPPV	☐	
		Hepatitis A	☐	
		Other:_________________	☐	

Your baby is 26 weeks old

Completion date: ____/____/____

1. In the past 7 days, how often was your baby feed each item listed below on average? Consider everyone that has feed the baby. Include snacks and night-time feeds.[A]

		Number feeds per day	Number of days given	Not given
1.1	Breast milk via breast feeding	_____	_____	☐
1.2	Expressed breast milk	_____	_____	☐
1.3	Formula	_____	_____	☐
1.4	Water	_____	_____	☐
1.5	Sugar water	_____	_____	☐
1.6	Cow's milk*	_____	_____	☐
1.7	Other milk**	_____	_____	☐
1.8	Fruit juices	_____	_____	☐
1.9	Other drinks‡	_____	_____	☐

* Includes plain, low-fat, evaporated and flavoured varieties of cow's milk. Include milk added to other foods such as cereal.

** Such as soy, rice, almond or goat milk. Include milk added to other foods such as cereal.

‡ Includes vegetable juice, soft drinks, cordial, sports drinks, yoghurt drinks, tea, coffee, etc.

2. The following questions relate to occasions when your baby consumed **breast milk**.

☐ *My baby did not consume breast milk this week (proceed to Question 3).*

2.1 Average feed duration when being breast feed:

_____________minutes

2.2 Average volume *consumed* when provided expressed breast milk:

_____________mL

☐ Never provided expressed breast milk *(proceed to Question 2.5)*

2.3 How long was expressed breast milk stored before it was fed to your baby (tick all that applies)?

☐ Kept at room temperature ☐ 1 day or less in refrigerator
☐ 2-3 days in refrigerator ☐ 3-4 days in refrigerator
☐ 5-7 days in refrigerator ☐ Frozen (for any length of time)

2.4 On the majority of occasions, how were bottles containing expressed breast milk warmed (tick only one)?

☐ Did not warm ☐ Hot water[Ψ]\bottle warmer
☐ Microwave ☐ Other_____________________________

[A] Refer to "Footnote A: Instructions for Question 1" on Page 2 for further clarification about answer this question

2.5	Did you (the baby's mother) take any antibiotics this week? Please provide details about the type of antibiotic and dosage as described in Question 7.<br>☐ No ☐ Unsure<br>☐ Yes Type: ______________________________<br>Dose volume: ________________________<br>Doses per day: _______________________<br>Number of days: ______________________	

ψ When bottle containing the milk is placed into a container of hot water

3. The following questions relate to occasions when your baby consumed **formula**.
   ☐ *My baby did not consume formula this week (proceed to Question 4).*

3.1	Average volume consumed with provided formula:<br>_____________mL
3.2	What were the brand(s) and product name(s) of the formula(s) your baby been given. For example, "Wyeth S-26 GOLD Newborn" or "Karicare Aptamil® Delact"<br>1.____<br>2.____<br>3.____<br>☐ Unknown
3.3	Was the formula prepared according to the manufacturer's instructions?<br>☐ Yes<br>☐ No (please provide details of how the formula was prepared)<br>____<br>____<br>____
3.5	On the majority of occasions, how were bottles containing formula warmed (tick only one)?<br>☐ Did not warm ☐ Prepared with hot water<br>☐ Hot water[ψ]\bottle warmer ☐ Microwave<br>☐ Other_____________________________

ψ When bottle containing the milk is placed into a container of hot water

4. The following question relates to occasions when your baby consumed **drinks other than breast milk, formula or plain water**. This includes cow's milk or milk from other non-dairy sources as well as fruit juices, vegetable juices, cordials, soft drinks, etc. Please state what the drink(s) were, the **brand of the product** (where applicable) and how much your baby consumed on occasions

these drinks were provided. Drink additives should also be included.[B] *If your baby has never received drinks other than breast milk, formula and water proceed to question 5.*

5. The following questions relate to occasions when your baby consumed **solid foods** this week. Please indicate which of the follow foods your baby received and, on each occasion your baby was offered that food, the average amount he/she consumed with respect to a "spoon" or "mound" code as indicated in the Instructions.[C]

		Not at all	1-3 times per week	4-6 times per week	1-2 times per day	3-4 times per day	5 or more times per day	Average amount eaten per occasion
5.1	Vegetables including potato and baked beans	☐	☐	☐	☐	☐	☐	________
5.2	Fruit	☐	☐	☐	☐	☐	☐	________
5.3	Egg	☐	☐	☐	☐	☐	☐	________
5.4	Dairy products including butter, cheeses, cream, yoghurt, custard and ice cream	☐	☐	☐	☐	☐	☐	________
5.5	Wheat containing products including breads, biscuits, rusks, cakes, breakfast cereals, pastries, pasta, couscous and dim sims	☐	☐	☐	☐	☐	☐	________
5.6	Rice and/or rice containing products including rice cereals (e.g. Farax), rice cakes, rice noodles and rice flour	☐	☐	☐	☐	☐	☐	________
5.7	Soy containing products including tofu, miso, soy sauce, soy flour and soy-based dairy substitutes	☐	☐	☐	☐	☐	☐	________
5.8	Corn and/or corn containing products including popcorn, polenta, corn flakes, corn tortillas and corn flour (including products thickened with e.g. gravy)	☐	☐	☐	☐	☐	☐	________

[B] Refer to "Footnote B: Instructions for Question 4" on Page 3 for further clarification about answer this question
[C] Refer to "Footnote C: Instructions for Question 5" on Page 3 for further clarification about answer this question

		Not at all	1-3 times per week	4-6 times per week	1-2 times per day	3-4 times per day	5 or more times per day	Average amount eaten per occasion
5.9	Oats or oat containing products including porridge, oatmeal, muesli, ANZAC biscuits, bran and oat flour	☐	☐	☐	☐	☐	☐	________
5.10	Barley or rye containing products including barley water, many minestrone-style soups and rye bread	☐	☐	☐	☐	☐	☐	________
5.11	Nuts including peanut butter	☐	☐	☐	☐	☐	☐	________
5.12	Foods containing pork, lamb, chicken, turkey, or other poultry	☐	☐	☐	☐	☐	☐	________
5.13	Foods containing beef and veal	☐	☐	☐	☐	☐	☐	________
5.14	Foods containing fish or shellfish	☐	☐	☐	☐	☐	☐	________

If your baby consumed any of the solid foods outlined above, please provide some additional information about what those foods were with respect to each category. This may include the **name of the food**, the **brand of the product** (where applicable) and whether the food was **modified** (for example: low-fat, added calcium, wholemeal etc.).[D] Please also list any foods your baby consumed that do not fall in the above categories.

5.15

[D] Refer to "Footnote D: Instructions for Question 5" on Page 5 for further clarification about answer this question

What type of methods we used to cook your baby's solid foods (tick all that applies)?

5.16	What type of methods we used to cook your baby's solid foods (tick all that applies)?			
		☐ Did not cook	☐ Baking	☐ Steaming
		☐ Grilling	☐ Roasting	☐ Boiling
		☐ Stewing	☐ Frying	☐ BBQ
		☐ Microwaving	☐ Other________________________________	

6. The following questions relates to the use of dummies/pacifiers.

6.1	Do you regularly offer your baby a dummy/pacifier?		
		☐ Yes	☐ No
6.2	Did you use any sweeteners on the dummy/pacifier?		
		☐ No	☐ Yes, honey
		☐ Yes, other ____	

7. The following questions relate to occasion when your baby was given **supplements and medications**.[E] Please state the brand, what the volume of a "dose" was (e.g. 5 mL, 1 tablet, etc.), how many doses were given each day and on how many days it was given **this week**.

		Dose volume	Doses per day	Days
7.1	Supplements			
	☐ Multivitamin (brand:____________________)	_____	_____	_____
	☐ Omega-3/fish oil (brand:________________)	_____	_____	_____
	☐ Iron (brand:___________________________)	_____	_____	_____
	☐ Other:________________________________	_____	_____	_____
7.2	Antibiotics			
	Name:__________________________________	_____	_____	_____
	Name:__________________________________	_____	_____	_____
	Name:__________________________________	_____	_____	_____
7.3	Other as outlined in *Footnote E* (please specify)			
	Name:__________________________________	_____	_____	_____
	Name:__________________________________	_____	_____	_____
	Name:__________________________________	_____	_____	_____

[E] Refer to "Footnote E: Instructions for Question 7" on Page 6 for further clarification about answer this question

8. Please describe your child's health this week, specifying any **fevers** and/or illnesses that required you to visit a healthcare profession (e.g. child health nurse, doctor, naturopath, etc.)

9. Did your child receive any vaccinations this week, either scheduled or optional? If you child was not given the vaccination as per the National Immunisation Program (NIP) guidelines (for example, one particular vaccine was not given but others were) please provide details.

	Description	Timing	As per NIP guidelines	Variation of NIP guidelines (give details)
9.1	Scheduled	Birth	☐	
		2 months	☐	
		4 months	☐	
		6 months	☐	
		12 months	☐	
9.2	Optional	Influenza	☐	
		Pneumococcal 13vPCV	☐	
		Pneumococcal 23vPPV	☐	
		Hepatitis A	☐	
		Other:_________________	☐	

Contacting the ENDIA Study Staff

If you have any questions about how to complete the ENDIA Infant Feeding Diary, please contact your Research Nurse (Research Assistant):

Name: Meredith Krieg
Position: Research Nurse
Telephone: (08) 8161 7349
Email: Meredith.Krieg@health.sa.gov.au

If you want any further information concerning this project or if you have any medical problems which may be related to your involvement in the project, please speak to your research nurse. If you do not wish to speak with your research nurse you can also contact the following people:

Name: Megan Penno
Position: Local study coordinator
Telephone: (08) 8161 8747
Email: Megan.Penno@adelaide.edu.au

Name: Professor Jenny Couper
Position: Principal Investigator (Medical Contact)
Telephone: (08) 8161 6242
Email: Jennifer.Couper@adelaide.edu.au

If you have any complaints about any aspect of the project, the way it is being conducted or any questions about being a research participant in general, then you may contact:

Name: Ms Brenda Penny
Position: HREC Office contact
HREC: Women's and Children's Health Network HREC
Telephone: (08) 8161 6521
Email: Brenda.Penny@health.sa.gov.au

Name: Dr Tamara Zutlevics
Position: Executive Officer of HREC approving this research
HREC: Women's and Children's Health Network HREC
Telephone: (08) 8161 6149
Email: Tamara.Zutlevics@health.sa.gov.au
