# Supplementary material for: Environmental determinants of islet autoimmunity (ENDIA): a pregnancy to early life cohort study in children at-risk of type 1 diabetes
Source: BMC Pediatr. 2013 Aug 14;13:124. doi: 10.1186/1471-2431-13-124 (PMC3751791; doi:10.1186/1471-2431-13-124)
Supplement: Additional file 3 — The ENDIA Maternal Lifestyle in Pregnancy questionnaire developed for this study. [file 1471-2431-13-124-S3.pdf]

ENDIA: Environmental Determinants of Islet Autoimmunity	Version number: 121009	
ENDIA environmental determinants of islet autoimmunity	**Maternal Lifestyle in Pregnancy Questionnaire**	
	Date of completion ___/___/___	Affix Participant Clinical Label Here

1. In the past three months, on how many days did you consume any of the following drinks:

		Never	Less than one day per month	1-3 days per month	1 day per week	2-5 days per week	More than 5 days per week
1.1	Cow's milk*	☐	☐	☐	☐	☐	☐
1.2	Other milk including soy, rice, almond, goat or sheep milk*	☐	☐	☐	☐	☐	☐
1.3	Coffee containing caffeine (includes iced coffee)	☐	☐	☐	☐	☐	☐
1.4	Decaffeinated coffee (includes iced coffee)	☐	☐	☐	☐	☐	☐
1.5	Tea containing caffeine	☐	☐	☐	☐	☐	☐
1.6	Decaffeinated/herbal tea	☐	☐	☐	☐	☐	☐
1.7	Other drinks containing caffeine (e.g. cola, Red Bull, V)	☐	☐	☐	☐	☐	☐

* Include flavoured milk and milk added to tea, coffee, cereal, etc.

On the days when you did consume these drinks in the past three months, how much on average did you consume based on the suggested average serving sizes:

		Average serving size	Did not consume	Less than 1 serve	1-2 serves	2-3 serves	More than 3 serves
1.8	Cow's milk*	250 mL glass	☐	☐	☐	☐	☐
1.9	Other milk including soy, rice, almond, goat or sheep milk*	250 mL glass	☐	☐	☐	☐	☐
1.10	Coffee containing caffeine (includes iced coffee)	1 small cup	☐	☐	☐	☐	☐
1.11	Decaffeinated coffee (includes iced coffee)	1 small cup	☐	☐	☐	☐	☐
1.12	Tea containing caffeine	1 small cup	☐	☐	☐	☐	☐
1.13	Decaffeinated/herbal tea	1 small cup	☐	☐	☐	☐	☐
1.14	Other drinks containing caffeine (e.g. cola, Red Bull, V)	250 mL can	☐	☐	☐	☐	☐

* Include flavoured milk and milk added to tea, coffee, cereal, etc. For example, a small amount of cow's milk in tea may represent a ¼ cup. The milk component of white tea/coffee should be recorded in addition to the tea/coffee itself.

2. In the past three months, how often did you consumed the following foods:

		Never	Less than once per month	1-3 days per month	1 day per week	2-5 days per week	More than 5 days per week
2.1	Dairy products including butter, cheeses, cream, yoghurt, custard and ice cream	☐	☐	☐	☐	☐	☐
2.2	Soy containing products including tofu, miso, soy sauce, soy flour and soy-based dairy substitutes	☐	☐	☐	☐	☐	☐

Conducting staff member initials____
Entered into ENDIA database ____/____/____

		Never	Less than once per month	1-3 days per month	1 day per week	2-5 days per week	More than 5 days per week
2.3	Wheat containing products including breads, biscuits, cakes, breakfast cereals, pastries, pasta, couscous, dim sims and wontons	☐	☐	☐	☐	☐	☐
2.4	Barley or rye containing products including barley water, many minestrone-style soups and rye bread	☐	☐	☐	☐	☐	☐
2.5	Rice and/or rice containing products including rice milk, rice cereals, rice cakes, rice noodles and rice flour	☐	☐	☐	☐	☐	☐
2.6	Corn and/or corn containing products including popcorn, polenta, corn flakes, corn tortillas and corn flour	☐	☐	☐	☐	☐	☐
2.7	Oats or oat containing products including porridge, oatmeal, muesli, ANZAC biscuits, bran and oat flour	☐	☐	☐	☐	☐	☐

3. Please provide details of any dietary supplements you have taken in the past three months, the frequency you took them and size of the dose (for example, 1 tablet, 5 mL, etc.). The frequency is based on the estimated number of days you took the supplement. For example, if you took a multivitamin every 2nd day this would equate to 45 days in the past three months and therefore you would tick the "31-60 days" box.

		Frequency					Amount taken
		Never	0-30 days	31-60 days	61-90 days	Daily	
3.1	Pregnancy/lactation supplement brand:____________________________	☐	☐	☐	☐	☐	_____
3.2	Other Multivitamin brand:____________________________	☐	☐	☐	☐	☐	_____
3.3	Omega-3/fish oil/cod-liver oil brand:____________________________	☐	☐	☐	☐	☐	_____
3.4	Iron brand:____________________________	☐	☐	☐	☐	☐	_____
3.5	Other: brand:____________________________	☐	☐	☐	☐	☐	_____

4. Did you take any antibiotics this week? Please provide details about the type of antibiotic and dosage as described in Question 7.

☐ No ☐ Unsure

☐ Yes Type: ________________________________
Dose volume: __________________________
Doses per day: ________________________
Number of days: _______________________

5. In the past three months, how many cigarettes did you smoke on average (tick one item only):

5.1	I did not smoke and have never been a smoker	☐
	I did not smoke but have previously been a smoker	☐
	Less than 1 cigarette per week	☐
	2 - 6 cigarettes per week	☐
	1 - 5 cigarettes per day	☐
	6 - 10 cigarettes per day	☐
	More than 10 cigarettes per day	☐

Which one of the following best describes your current household (tick one item only)?

5.2	Members of my household smoke at home, inside the house	☐
	Members of my household smoke at home, only outside the house and never inside	☐
	Members of my household are smokers but they don't smoke at home, inside or outside	☐
	Nobody in my household is a smoker	☐

6. How many of the following people, including yourself, currently reside in your household:

Adults:_________________ Children:_______________

7. Which of the following describes your household now (tick all that applies):

7.1	We have a dog that comes inside	☐
7.2	We have a dog that lives outside and doesn't come inside	☐
7.3	We have a cat that comes inside	☐
7.4	We have a cat that lives outside and doesn't come inside	☐
7.5	We have a furred pet that is not a cat or dog	☐
7.6	We do not have any furred pets – cats, dogs or otherwise	☐

Please now complete the Pregnancy Physical Activity Questionnaire. Thank you.
